# Deep proteomic network analysis of Alzheimer’s disease brain reveals alterations in RNA binding proteins and RNA splicing associated with disease

**DOI:** 10.1186/s13024-018-0282-4

**Published:** 2018-10-04

**Authors:** Erik C. B. Johnson, Eric B. Dammer, Duc M. Duong, Luming Yin, Madhav Thambisetty, Juan C. Troncoso, James J. Lah, Allan I. Levey, Nicholas T. Seyfried

**Affiliations:** 1 Department of Neurology, Emory University School of Medicine, Whitehead Building—Suite 505C, 615 Michael Street, Atlanta, GA 30322 USA; 2 Department of Biochemistry, Emory University School of Medicine, Atlanta, GA 30322 USA; 3 Johns Hopkins School of Medicine, Baltimore, MD 21205 USA; 4 National Institute on Aging, National Institutes of Health, Bethesda, MD 20892 USA

**Keywords:** Alzheimer’s disease, Proteomics, Proteogenomics, RNA binding protein, RNA splicing

## Abstract

**Background:**

The complicated cellular and biochemical changes that occur in brain during Alzheimer’s disease are poorly understood. In a previous study we used an unbiased label-free quantitative mass spectrometry-based proteomic approach to analyze these changes at a systems level in post-mortem cortical tissue from patients with Alzheimer’s disease (AD), asymptomatic Alzheimer’s disease (AsymAD), and controls. We found modules of co-expressed proteins that correlated with AD phenotypes, some of which were enriched in proteins identified as risk factors for AD by genetic studies.

**Methods:**

The amount of information that can be obtained from such systems-level proteomic analyses is critically dependent upon the number of proteins that can be quantified across a cohort. We report here a new proteomic systems-level analysis of AD brain based on 6,533 proteins measured across AD, AsymAD, and controls using an analysis pipeline consisting of isobaric tandem mass tag (TMT) mass spectrometry and offline prefractionation.

**Results:**

Our new TMT pipeline allowed us to more than double the depth of brain proteome coverage. This increased depth of coverage greatly expanded the brain protein network to reveal new protein modules that correlated with disease and were unrelated to those identified in our previous network. Differential protein abundance analysis identified 350 proteins that had altered levels between AsymAD and AD not caused by changes in specific cell type abundance, potentially reflecting biochemical changes that are associated with cognitive decline in AD. RNA binding proteins emerged as a class of proteins altered between AsymAD and AD, and were enriched in network modules that correlated with AD pathology. We developed a proteogenomic approach to investigate RNA splicing events that may be altered by RNA binding protein changes in AD. The increased proteome depth afforded by our TMT pipeline allowed us to identify and quantify a large number of alternatively spliced protein isoforms in brain, including AD risk factors such as BIN1, PICALM, PTK2B, and FERMT2. Many of the new AD protein network modules were enriched in alternatively spliced proteins and correlated with molecular markers of AD pathology and cognition.

**Conclusions:**

Further analysis of the AD brain proteome will continue to yield new insights into the biological basis of AD.

**Electronic supplementary material:**

The online version of this article (10.1186/s13024-018-0282-4) contains supplementary material, which is available to authorized users.

## Background

Alzheimer’s disease (AD) is the most common age-related neurodegenerative disease, and currently affects more than 46 million people worldwide [1]. The burden of this disease is rapidly growing as the population ages, and interventions to treat or prevent the disease are urgently needed. While AD is currently defined by cognitive decline in the presence of amyloid plaque and tau tangle accumulation within the brain, the altered biochemical and cellular processes that eventually lead to changes in cognition and pathology are not well understood. A better understanding of these altered processes may yield insight into new drug targets and biomarkers for AD. Systems-based approaches such as weighted gene co-expression network analysis (WGCNA) can be used to analyze biochemical and cellular changes in brain, and are useful to help capture the complexity of perturbations in biological networks that are related to disease [2–4]. We recently described a weighted protein correlational network analysis (WPCNA) of post-mortem brains from patients with AD, asymptomatic AD (AsymAD), and controls [5]. We found protein network modules that correlated with both cognition and AD pathology. These modules were enriched for AD risk loci identified by genome-wide association studies (GWAS), and contained a large number of glial proteins. Many of the modules we identified were distinct from mRNA network modules generated from a separate AD post-mortem brain cohort, suggesting that mRNA and protein network analyses can generate both complementary and unique information.

The number of proteins that can be quantified in a sample cohort is a fundamental limiting factor in the depth and complexity of any network built from proteomic data, and consequently the amount of information that can be gleaned from such networks. In our previous analysis of AD, AsymAD, and control brains from the Baltimore Longitudinal Study of Aging (BLSA) [6] cohort, we were able to quantify only 2,736 proteins across 97 dorsolateral prefrontal cortex (DLPFC) and precuneus brain tissues using label-free quantification (LFQ) by liquid chromatography tandem mass spectrometry (LC-MS/MS), despite the fact that we were able to identify > 5000 proteins by LC-MS/MS across the set of brain samples [5]. This reduction in quantifiable proteins by LFQ LC-MS/MS is a consequence of the stochastic nature of data-dependent acquisition techniques that leads to the well-known “missing value” problem [7], where the same ions are not consistently chosen for MS/MS analysis across all runs, or the peptide precursor ions are not matched effectively across runs. One strategy to minimize the missing value problem is to measure peptide and protein levels using a multiplex tagging approach with isobaric tandem mass tags (TMTs) [8–11]. The most recent generation of TMTs can be used to report the relative levels of a given peptide from a pool consisting of up to 11 separate and independent samples [10]. Using an appropriate pooled sample study design and mass spectrometry instrumentation that can perform MS^3^ reporter quantitation, missing values can be minimized within an experimental cohort using a TMT approach while avoiding dynamic range compression effects [8]. In this study, we used a new pipeline with TMTs, coupled with offline prefractionation, to profile a much deeper proteome in the same BLSA DLPFC tissues previously analyzed by online “single-shot” LFQ. This approach allowed us to quantify 6,533 proteins across the entire cohort—over double the depth achieved in our previous study. The increased depth of proteome coverage allowed us to build a protein network that consisted of approximately threefold more protein modules, two-thirds of which shared little overlap with the modules previously identified in our LFQ network. One of the most unique modules contained strong enrichment in AD risk loci identified by the International Genetics of Alzheimer’s Project (IGAP) GWAS [12], correlated with tau tangle burden, and contained more glial than neuronal proteins. We also used differential expression analysis on the enlarged proteomic dataset to identify proteins that have altered levels among AD, AsymAD, and control brains, even after accounting for changes in cellular abundance. RNA binding proteins emerged as a family of proteins that was increased in abundance in AD, and these proteins were enriched in modules that correlated with tau tangle pathology. Based on this finding, we explored changes in RNA splicing manifested at the protein level that may occur due to potential RNA binding protein dysfunction in AD. To do so, we developed a new proteogenomic pipeline that used RNA-seq data from control and AD brain to predict alternative exon-exon junction splicing events not present in conventional protein databases. This proteogenomic approach, coupled with the increased depth of proteome coverage and superior quantitation afforded by our TMT pipeline compared to our previous LFQ approach, allowed us to identify and quantify a number of alternative exon-exon splicing events in brain at the protein level, including alternative exon-exon junctions in AD risk factor proteins such as BIN1, PICALM, PTK2B, and FERMT2. Many of the identified alternative exon-exon junction splicing events were highly enriched in modules unique to the TMT network, and correlated with disease, suggesting a potential role for aberrant RNA splicing in AD pathogenesis.

## Methods

### Tissue samples

Fresh frozen brain tissue blocks from dorsolateral prefrontal cortex (Brodmann area 9) were used for analysis, as described previously [5]. Frozen aliquots from the same brain homogenate were used for LFQ and TMT analysis. Symptomatic AD (*n* = 20), asymptomatic AD (AsymAD) (*n* = 14), and control (*n* = 13) cases were processed and analyzed. In addition to these *n* = 47 cases, mild cognitive impairment (MCI) cases (*n* = 11) were homogenized separately on a different day and included in the batched TMT-MS design, but were later excluded from the analysis due to a preparation batch effect that was refractory to post-hoc correction. Sample information is given in Additional file 1: Table S1 and Additional file 2: Table S2. The TMT-MS experimental design is shown in Additional file 3: Table S3.

### Tissue homogenization

Each tissue piece (approx. 100 mg wet weight) was homogenized in 500 μL of urea lysis buffer (8 M urea, 100 mM NaH_2_PO_4_, pH 8.5), supplemented with 5 μL (100× stock) HALT protease and phosphatase inhibitor cocktail (Pierce) using a Bullet Blender (Next Advance) and 750 mg of steel beads (Next Advance). Protein supernatants were then transferred to new 1.5 mL Eppendorf tube and sonicated (Sonic Dismembrator, Fisher Scientific) 3 times for 5 s with 15 s intervals of rest at 30% amplitude. Protein concentration was determined by the bicinchoninic acid (BCA) method, and samples were frozen in aliquots at − 80 °C. Protein integrity was checked by one-dimensional SDS-PAGE (Additional file 8: Figure S1). The MCI case samples were homogenized on a later day than the control, AsymAD, and AD cases, but digestion prior to TMT labeling was performed at the same time.

### SDS-page

Protein homogenates (100 μg) were mixed with Laemmli sample buffer and β-mercaptoethanol (3% *v*/v), and incubated for 5 min at 95 °C. After cooling, 10 μg protein was loaded into Bolt 10% Bis-Tris Plus gels (Invitrogen) and electrophoresed for 30 min at 160 V. Gels were then stained with Coomassie Blue for protein visualization.

### Protein digestion, TMT labeling, and ERLIC fractionation

Protein homogenates (100 μg) were treated with 1 mM dithiothreitol (DTT) at 25 °C for 30 min, followed by 5 mM iodoacetimide (IAA) at 25 °C for 30 min in the dark. Protein was digested with 1:100 (*w*/w) lysyl endopeptidase (Wako) at 25 °C overnight. Resulting peptides were desalted with a Sep-Pak C18 column (Waters). All samples were dried down completely using a Savant SpeedVac (ThermoFisher Scientific). In addition to the 58 case samples, a global internal standard (GIS) mixture of case sample homogenates (*n* = 60, 30 control and 30 AD) taken from multiple different patient cohorts was generated by mixing each sample equally by protein amount prior to TMT labeling on a designated reporter channel. TMT labeling was performed per the manufacturer’s protocol and as previously described [10]. Briefly, the reagents were equilibrated to room temperature. Dried peptide samples (100 μg each) were resuspended in 100 μl of 100 mM TEAB buffer (supplied with the kit). Anhydrous acetonitrile (ACN) (41 μl) was added to each labeling reagent tube and the peptide solutions were transferred into their respective channel tubes. The reaction was incubated for 1 h and quenched for 15 min afterward with 8 μl of 5% hydroxylamine. Samples were combined according to the batch design shown in Additional file 3: Table S3, and dried down to 100 μl to remove ACN. The combined samples were then desalted using a Sep-Pak C18 column (Waters) and dried down to approximately 5 μl. The labeled peptide sample batches were each further diluted with 100 μl of 90% ACN and 0.1% acetic acid (buffer A) and loaded onto an offline electrostatic repulsion–hydrophilic interaction chromatography (ERLIC) fractionation HPLC system [10, 13]. A total of 40 fractions were collected over a 40-min gradient from 0 to 28% Buffer B (30% ACN and 0.1% formic acid). The 40 fractions were combined down to 20 and dried down to completeness.

### LC-MS/MS

Dried peptide fractions were resuspended in 30 μl of peptide loading buffer (0.1% formic acid, 0.03% trifluoroacetic acid, 1% acetonitrile). Peptide mixtures (2 μl) were separated on a self-packed C18 (1.9 μm Dr. Maisch, Germany) fused silica column (25 cm × 75 μM internal diameter; New Objective) by a Dionex Ultimate 3000 RSLCNano and monitored on a Fusion mass spectrometer (ThermoFisher Scientific). Elution was performed over a 140-min gradient at a rate of 300 nl/min with buffer B ranging from 3 to 80% (buffer A: 0.1% formic acid in water, buffer B: 0.1% formic acid in acetonitrile). The mass spectrometer was programmed to collect at the top speed for 3 s cycles in synchronous precursor selection (SPS)-MS3 mode [10, 14]. The MS scans (380–1500 m/z range, 200,000 AGC, 50 ms maximum ion time) were collected at a resolution of 120,000 at m/z 200 in profile mode. CID MS/MS spectra (2 m/z isolation width, 35% collision energy, 10,000 AGC target, 35 ms maximum ion time) were detected in the ion trap. HCD MS/MS/MS spectra (2 m/z isolation width, 65% collision energy, 100,000 AGC target, 120 ms maximum ion time) of the top 5 MS/MS product ions were collected in the Orbitrap at a resolution of 60000 [10]. Dynamic exclusion was set to exclude previous sequenced precursor ions for 30 s within a 10 ppm window. Precursor ions with + 1 and + 8 or higher charge states were excluded from sequencing.

### Database search and quantification via TMT SPS-MS3 intensities

MS/MS spectra were searched against a Uniprot human database (downloaded on 04/15/2015 with 90,411 target sequences) with Proteome Discoverer 2.1 (ThermoFisher Scientific). The database included all Swiss-Prot-curated (canonical) plus TrEMBL (unreviewed) sequences, totaling 90,411 FASTA sequence entries. Methionine oxidation (+ 15.9949 Da), asparagine, and glutamine deamidation (+ 0.9840 Da) and protein N-terminal acetylation (+ 42.0106 Da) were variable modifications (up to 3 allowed per peptide); static modifications included cysteine carbamidomethyl (+ 57.0215 Da), peptide N-terminus TMT (+ 229.16293 Da), and lysine TMT (+ 229.16293 Da). Only peptides resulting from LysC digestion were considered, with up to two miscleavages, in the database search. A precursor mass tolerance of ±20 ppm and a fragment mass tolerance of 0.6 Da were applied. Spectra matches were filtered by Percolator [15] to a peptide-spectrum match false discovery rate of < 1%. Strict parsimony was observed for peptide to protein matching, and only razor and unique peptides were used for abundance calculations. Log_2_ ratio of sample over the GIS was used for comparison across all samples.

### TMT quantitative data normalization

GIS mixture (MS^3^ TMT reporter channel m/z 126) provided as Proteome Discoverer 2.1 script output was checked for extreme outlier values of log_2_(0.01) and log_2_(100), i.e. ±6.64; these values were excluded from analysis. Furthermore, proteins with more than 4 unquantifiable batches (out of a total of 8 batches) due to 0 or NA value for the GIS channel 126 reporter Proteome Discoverer 2.1-normalized value (pre-ratio calculation) were excluded from consideration. Finally, proteins with more than 23 missing log_2_(ratio) values were excluded from analysis, and then 11 MCI cases were dropped, leaving a matrix of *n* = 47 control, AsymAD, and AD cases with no more than 23 missing values (< 50%) per protein measurement, for a total of 6532 proteins. Amyloid-β log_2_(ratio) represented by TMT peptide level quantitation of the APP LVFFAEDVGSNK peptide was added to the final 6533 × 47 protein abundance matrix.

### Digital sorting algorithm for cell type weight analysis of tissue proteomes

The covariate-unregressed, normalized abundance matrix described above was collapsed to average protein abundance measurements for unique gene symbols (*n* = 5,839) using WGCNA::collapseRows() function [16]. Two thousand one hundred thirty two cell type marker gene symbols from pure cell types of mouse brain [17] (referred to as the Sharma dataset) which we previously defined via thresholding used for cell type enrichment analyses of human proteome coexpression modules [5, 17] were converted from mouse to human gene symbols using biomaRt R interface to the public Ensembl datamart as of July 2017 [18]. From this set, 895 gene symbols representing collapsed and averaged protein abundances with no missing quantification values across the 47 BLSA case tissue samples overlapped the Sharma quantitative dataset. The overlapping marker measurements from Sharma purified brain cell types and our BLSA middle frontal gyrus samples were input into the DSA v1.0 R package [19] and estimated weights were found using the DSA::EstimateWeight() function.

### Regression for covariates

A naïve first pass regression was performed by considering age, sex, post-mortem interval (PMI), and disease status group contributions to each sample-specific protein abundance measurement set (*n* = 47), explicitly modeled using 1000 iterations of ordinary nonparametric bootstrap regression. Then age, sex, and PMI covarying components of the measurement were subtracted to arrive at a regressed protein abundance measurement set. This approach was repeated for all 6,533 proteins in the abundance matrix.

A second, two-pass regression scheme was performed by first considering DSA estimated cell type weight for the four Sharma dataset brain cell types (microglia, astrocytes, neurons, and oligodendroglia) as four sets of variables for regression. Following normalization of cell type abundance variation across the samples, the prior age, sex, and PMI regression scheme was used to remove these covariate effects. Only the first pass regressed protein abundance matrix was used for WPCNA. Importantly, missing values did not require imputation for bootstrap covariate regression.

### Weighted protein correlation network analysis (WPCNA)

Threshold power Beta for reduction of false positive correlations (i.e. the beneficial effect of enforcing scale free topology) was sampled in increments of 0.5 and selected as the lowest power at which scale free topology R^2^ was approximately 0.80, or in the case of the cell type weight-regressed network, the power at which a horizontal asymptote (plateau) was nearly approached, near a scale free topology R^2^ of (0.80). Other parameters were selected as previously optimized for protein abundance networks [5]. Thus, for the signed network build on protein abundances after naïve age, sex, and PMI regression, parameters were input into the WGCNA::blockwiseModules() function as follows: Beta (power) 8.0, mergeCutHeight 0.07, pamStage TRUE, pamRespectsDendro TRUE, reassignThreshold *p* < 0.05, deepSplit 2, minModuleSize 17, replaceMissing TRUE, corType bicor, maxBlockSize greater than the total number of proteins (6,533), and TOMDenominator mean.

### Gene ontology (GO) functional analysis of WPCNA modules

GO analysis for module membership was performed using GO-Elite [20] with the background set to all 5,839 gene symbols quantified in this study. Gene lists per module were subjected to Fisher exact overlap test in the python command line version of GO-Elite v1.2.5 for species setting Hs against the current (downloaded June 2017) annotation database for Biological Process, Molecular Function, and Cellular Component terms. Cytoscape with the EnrichmentMAP app [21] was used to visualize ontology representation, overlap, and relatedness.

### Statistics

Differential expression analysis was performed as previously described [5]. Briefly, differentially expressed proteins were found using one-way ANOVA followed by Tukey’s comparison post hoc (*p* value < 0.05). Volcano plots were generated with the ggplot2 package in R. Custom R scripts were used to visualize overlap of differentially expressed targets with WPCNA modules.

MAGMA [22] for *p* value calculation of GWAS target enrichment in WPCNA modules was performed as previously described [5]. Hypergeometric overlap significance tests, namely one-tailed Fisher exact and two-tailed overrepresentation analysis, were performed as previously described [5].

### Proteogenomic RNA alternative splicing analysis based on gapped transcriptome reads

The GSNAP algorithm with novel splicing flag (-N) on [23] was used to realign raw short paired end RNA-Seq reads of 3 control and 3 AD cases from the University of Kentucky brain bank originally published in Bai, et al. [24] to the GRCh37 human genome build with contigs and the 16,569 nucleotide (nt) mitochondrial genome. Then all exon-exon junctions represented by 2 or more gapped reads across the 6 case sample cDNA libraries, with a minimum exonic overlap of 4 nt, were summarized using the R spliceSites bioconductor package. A custom R script and Excel formulas for string manipulation were used to extract LysC [K|P] peptides spanning exon-exon junctions (both with and without miscleavage at proline). All junction-spanning peptides considered were ones that had alternative events represented by other gapped reads that shared a left (5′) or right (3′) end with another set of gapped reads, and not “singleton” or brain constitutive exon-exon junctions. Peptides from different genomic sites that were 100% homologous to the junction-spanning peptides were considered duplicates and were removed from consideration. The resulting list of annotated alternative exon-exon junction-spanning peptides (*N* > 58,319) detected in brain transcriptome were concatenated as FASTA entries to the April 2015 human Uniprot database, and then Proteome Discoverer 2.1 was used to search and quantify peptide reporter channels across all 8 batches of TMT data with parameters otherwise as described above for the initial search. Peptide summary output for each of the 8 batches was opened in Excel, and all peptides annotated in the expanded human database as brain-specific alternative exon-exon junction peptides—including different modified forms of the same fully LysC digested peptides—were found and summed using the Excel sumif() function. These unified quantitations were performed over the different post-translationally modified states of the same peptide (e.g., N-terminal acetylation, or N/Q deamidation, or M oxidation) for all alternative exon-exon junction peptides in the peptide-level summary output for each of the 8 10-plex batches of ERLIC fractions. Quantitations of within-batch normalized abundances were then scaled across batches to set the average of all GIS measurements within batch to be identical across batches. The scaled, normalized, summed peptide abundances were log2-transformed; 9 negative values (< 1 before log2 transformation) were removed from the matrix. Regression for age, sex, and PMI covariation was performed in R on all log2 transformed values except for 781 that could not be regressed due to a high number of missing values. After regression, ANOVA with Tukey post hoc correction was performed on both regressed and unregressed values. The regressed alternative exon-exon junction peptide abundances were matched to the 50 WPCNA eigenproteins by calculating kME (correlation to module eigenprotein) for each peptide and assigning the peptide to the module with the highest correlation. For the purposes of avoiding spurious correlations, no more than 25 out of 47 missing values were allowed for any peptide. Venn and volcano plots were produced in R using vennDiagram, ggplot2, and/or plotly R packages.

## Results

### TMT quantification pipeline increases the depth of proteomic network analysis of human brain tissues

In our previous analysis of dorsolateral prefrontal cortex (DLPFC) brain tissue from AD, asymptomatic AD (AsymAD), and control cases from the Baltimore Longitudinal Study of Aging (BLSA) [6] cohort, we were able to identify 3,069 proteins with 10 % or less missing values across 47 DLPFC brain samples (excluding precuneus samples) using “single-shot” one-dimensional online reverse-phase HPLC fractionation and label-free quantitation (LFQ) [5]. This represented a reduction from 5138 total proteins identified across all DLPFC samples due to missing peptide quantitative values in greater than 10% of the samples. In order to address the limitation of LFQ by data-dependent LC-MS/MS when analyzing protein levels across multiple samples, we reprocessed and reanalyzed the same DLPFC homogenates using a multiplex isobaric tandem mass tag (TMT) labeling approach and synchronous precursor selection-based mass spectrometry (SPS-MS3) quantitation on a tribrid mass spectrometer, coupled with orthogonal offline prefractionation [8, 10]. As part of the new analysis approach, we also relaxed the data inclusion criteria to require missing values in < 50% rather than < 10% of the samples, given that the WGCNA algorithm for coexpression network analysis well-tolerates missing values up to 50%. We subsequently refer to this quantitation and analysis approach as our “TMT pipeline.” Using the TMT pipeline, we were able to identify and quantify 6,533 proteins, compared to 3,069 proteins using the previous single-shot LFQ strategy. The large majority of the increase in protein coverage was due to the superior quantitation provided by TMT labeling and prefractionation rather than the relaxed missing values tolerance threshold (Additional file 9: Figure S2). To validate that protein quantitation was similar using the two different quantitation approaches, we compared the relative levels of the amyloid-β (Aβ)17–28 peptide in each sample quantified by LFQ and TMT. The Aβ17–28 peptide is a proteolytic fragment of Aβ generated by both trypsin and LysC enzymatic digestion of the full-length Aβ peptide, and therefore represents a peptide with a very large change in abundance across the sample cohort due to aggregation of Aβ into amyloid plaques in AsymAD and AD cases [5]. An illustration of Aβ17–28 quantitation by TMT is shown in Additional file 10: Figure S3A, with correlation of this Aβ peptide measurement to cerebral amyloid plaque load in each case shown in Additional file 10: Figure S3C. We found a strong correlation (*r* = 0.85) between Aβ levels measured by LFQ and TMT quantitation approaches (Additional file 10: Figure S3B), suggesting that TMT with SPS-MS3 quantification was able to reliably quantify proteins over a large dynamic range, similar to the LFQ approach employed in our previous analysis.

**Fig. 1:**
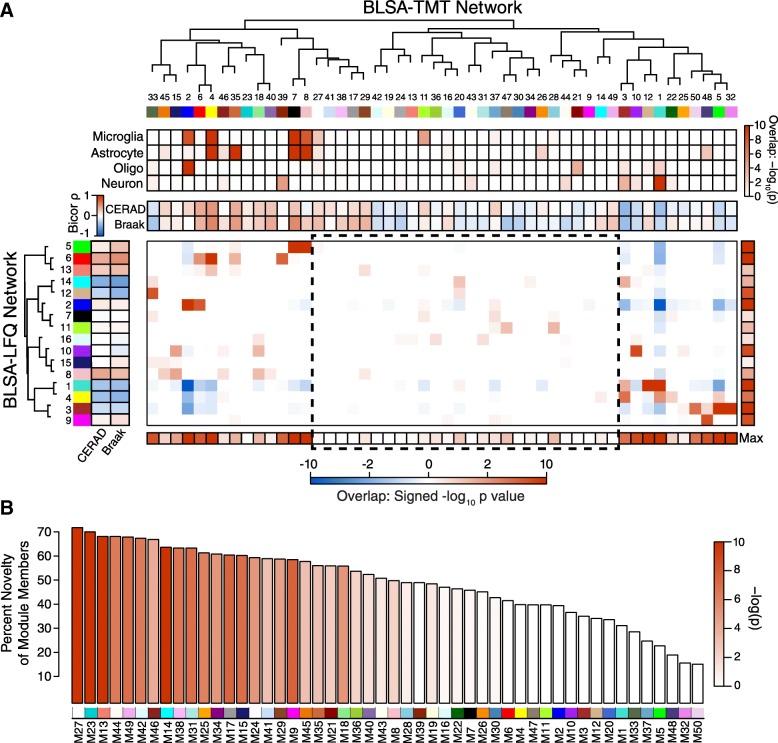
Correlational Network Analysis. **a**, **b** Proteins in frontal cortex from Alzheimer’s, asymptomatic Alzheimer’s, and control brains were analyzed by tandem mass spectrometry and quantified using either a label-free (LFQ)-based or tandem mass tag (TMT)-based quantification pipeline. The resulting data from each quantification approach were used to build separate correlational protein networks. **a** Modules in the LFQ-trypsin and TMT-LysC networks are represented by numbers (1–16 in LFQ and 1–50 in TMT) and a cognate color, and the correlational relationship among the different modules within a network is represented by dendrogram. The overlap of proteins within each TMT-LysC module with cell type specific protein markers from microglia, astrocytes, oligodendrocytes (oligo), and neurons is shown by single color heat map (increased red represents increased overlap). Correlation between modules and neuritic amyloid plaque burden (CERAD score) and tau tangle burden (Braak stage) is shown by two-color heat map for both TMT-LysC and LFQ-trypsin networks (red represents positive correlation, blue represents negative correlation). The CERAD score captures the type of amyloid plaque burden most closely associated with cognitive decline [53]. Module protein membership overlap between TMT-LysC and LFQ-trypsin modules is shown by two-color heat map in the large box (red indicates more overlap than expected, blue indicates less overlap than expected), with a summary of maximal overlap for each module with all other modules in the other network shown by single color heatmap in boxes labeled “Max”. All modules in the LFQ-trypsin network were preserved in the TMT-LysC network prior to correction for multiple comparisons. Preservation of LFQ-trypsin modules 7 and 15 in the TMT-LysC network was no longer significant after correction for multiple comparisons. The area highlighted by the dotted line box represents TMT-LysC modules that have little to no overlap in protein membership with LFQ-trypsin modules, representing protein modules unique to the TMT-LysC network. **b** Percent novelty of protein members within each module of the TMT-LysC network compared to the LFQ-trypsin network. Bars are color coded by heatmap for degree of significance by *P* value. *P* values shown in (**a**) and (**b**) are corrected by Benjamini-Hochberg FDR

We used the same correlational network analysis approach previously applied to the LFQ data to construct a protein correlational network from the TMT data (Fig. 1a). Whereas we were able to identify 16 modules of coexpressed proteins in the LFQ protein network, the increased proteomic depth afforded by the TMT pipeline increased the number of modules identified in the TMT network to 50. When comparing protein membership overlap between the modules in the two networks, most of the modules that were previously identified in the LFQ network were largely recapitulated in the TMT network, including LFQ modules M1, M4, M6, and others previously identified as strongly correlated with AD pathology [5]. These modules were renumbered in the TMT network due to enlargement of the network, and were occasionally split among several TMT modules, such as TMT modules M7 and M8 that corresponded to the LFQ module M5. TMT modules with cognate modules in the LFQ network that were strongly associated with AD traits included M1 and M3, which were negatively correlated with AD pathology, and M4 and M7, which were positively correlated with AD pathology, among others. However, in addition to the previously identified modules, the increased depth of the TMT network allowed us to identify a number of modules that shared little to no overlap with modules in the LFQ network. For instance, the most “unique” module, module 27 (M27), contained > 70% new protein members that were not identified in the LFQ network (Fig. 1b). The cell type “character” of each module can be assessed by examining the overlap of module protein membership with cell type specific protein expression data [5, 17]. While most of the new modules did not display a strong association with any of the four brain cell types we analyzed (microglia, astrocytes, oligodendrocytes, or neurons), M27 was predominantly glial in nature and correlated positively with tau tangle burden (Braak stage). Other modules unique to the TMT network that were significantly correlated with AD neuropathology included M17 and M29, which were associated with increased tau tangle burden, and M47, which was associated with decreased tau tangle burden. Additional TMT network modules associated with disease are illustrated in Additional file 11: Figure S4 and Supplementary Data. Therefore, the increased depth of proteome coverage afforded by the TMT pipeline allowed us to identify new modules that correlated with disease.

### AD genetic risk factors cluster in glial modules

**Fig. 2:**
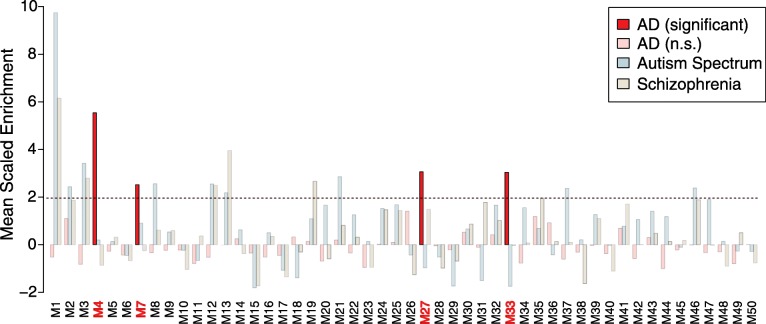
Enrichment of AD Genetic Risk Factors within TMT-LysC Network Modules. Enrichment of proteins contained within genetic regions identified by genome wide association studies (GWAS) as risk factors for AD, autism spectrum disorder, and schizophrenia was calculated for each module in the TMT-LysC network. Modules highlighted in dark red were significantly enriched for AD risk factors, and not for risk factors associated with autism or schizophrenia. The horizontal dotted line indicates a z-score level of enrichment of 1.96, or FDR < 0.05, above which enrichment was considered significant

Genetic variants that impart increased or decreased risk for AD have been mapped by genome wide association studies (GWAS), which collectively have identified over 20 genetic loci associated with AD at genome-wide significance, and many other loci that fall below genome-wide significance [12, 25]. We assessed whether the proteins encoded within these AD risk loci preferentially cluster within any of the modules in the TMT network [5, 22]. We found four modules that were uniquely enriched in gene products significantly associated with AD GWAS risk loci: M4, M7, M27, and M33 (Fig. 2). Three out of four of these modules (M4, M7, and M27) were predominantly glial in nature and correlated positively with amyloid plaque and tau tangle burden, whereas one module, M33, contained more proteins associated with oligodendrocytes and neurons, and correlated negatively with amyloid plaques and tau tangles (Fig. 1a). The proteins identified by GWAS that were enriched in each of these modules are highlighted in Additional file 12: Figure S5. Modules M4 and M7 showed strong overlap with two similar modules identified in the LFQ network, M6 and M5, respectively. M5 was also enriched in AD GWAS loci [5]. Module M27, however, was the most unique module in the TMT network compared to the previous LFQ network (Fig. 1b), and showed enrichment in GWAS protein candidates including PICALM, FERMT2, and TMEM106B, among others (Additional file 12: Figure S5). Therefore, the increased depth of the TMT network allowed us to identify unique protein modules that were both glial in nature and strongly enriched for AD genetic risk factors.

### Brain cell type changes and protein abundance differences between asymptomatic and symptomatic AD

**Fig. 3:**
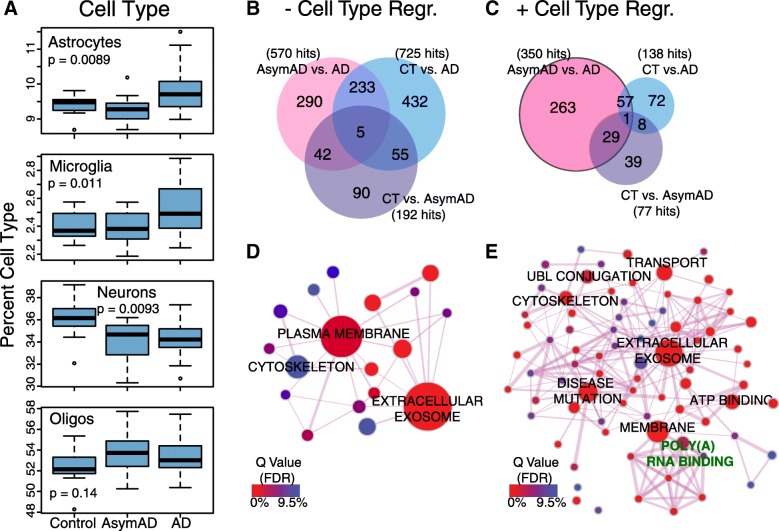
Protein Abundance Changes in AD Adjusted for Cell Type Changes. **a**–**e** The abundance of cell type-specific protein markers of astrocytes, microglia, neurons, and oligodendrocytes (oligos) was used to calculate the percentage of each cell type in control, asymptomatic AD (AsymAD), and AD brain tissue (**a**). **b** The number of proteins with significantly different levels among control (CT), AsymAD, and AD prior to adjustment for cell type populations changes observed in AsymAD and AD. The number of total proteins with differential abundance for AD, AsymAD, and CT is given in parentheses. **c** The number of proteins with differential abundance after adjustment for changes in cell type population by cell type deconvolution (regression). The circles in (**b**, **c**) are not drawn to scale. **d** Gene ontology (GO) network analysis of the AsymAD vs. AD proteins shown in (**b**), prior to cell type regression. **e** GO network analysis of the AsymAD vs. AD proteins in (**c**), after cell type regression. The nonspecific GO terms “cytoplasm” and “cytosol” were removed from the network. The RNA binding protein subnetwork is highlighted in green. An enlarged version of the network is given in Additional file 16: Figure S9, with a complete list of GO terms provided in Additional file 4: Table S4. *P* values in (**a**) were calculated after one-way ANOVA. Significance of each GO term in (**d**, **e**) is indicated by false discovery rate (FDR, or Q value). GO network analysis of AsymAD vs. control is provided in Additional file 15: Figure S8

The neuropathological changes that are quintessential for AD diagnosis—namely, the development of amyloid plaques and tau tangles—are considered to develop years before onset of the cognitive changes that characterize AD dementia [26]. This asymptomatic phase of AD (AsymAD) is currently considered a preclinical stage of the disease [27, 28]. Because our cohort contained brains from control (little to no AD pathology), AsymAD (AD pathology without cognitive symptoms), and AD (AD pathology with dementia) cases, we were able to examine the changes in brain cell type abundance for four different classes of cells across controls, AsymAD, and AD using a digital sorting algorithm [19, 29], and correlate these changes with amyloid plaque and tau tangle burden (Fig. 3a and Additional file 13: Figure S6). We found that astrocytes and microglia were increased in AD compared to AsymAD and control, and showed a strong correlation with tau tangle burden (Braak stage). The percentage of astrocytes and microglia also correlated with amyloid plaque burden, but less strongly than with tau tangle burden. The neuronal cell population decreased in both AsymAD and AD and correlated negatively with amyloid plaque burden. The oligodendrocyte population increased in AsymAD, and then decreased slightly in AD compared to AsymAD. While there was a weak positive correlation with amyloid plaque burden that approached statistical significance, the fraction of oligodendrocytes did not correlate with tau tangle burden. Therefore, the changes associated with progression to AsymAD are a decrease in neurons and an increase in oligodendrocytes mostly associated with amyloid burden. Progression from AsymAD to AD—that is, symptom onset—is associated with an increase in astrocytes and microglia, and is associated with neurofibrillary tangle burden.

We next asked whether these changes in cell type abundance are the primary drivers of changes in protein abundance among control, AsymAD, and AD, or whether there are changes in protein abundance by disease state that are independent of changes in cell type. TMT proteomic analysis allowed us to identify 1147 proteins that showed changes in abundance among control, AsymAD, and AD cases (Fig. 3b). Most of the proteins with altered abundance were observed when comparing control with AD cases, or AsymAD with AD cases, with relatively fewer proteins that differed between control and AsymAD. To account for changes in cell type on changes in protein abundance between groups, we used our estimates of cell type changes to deconvolute this effect from changes in protein abundance [19, 29], and then reanalyzed our pairwise group comparisons of differentially abundant proteins after deconvolution. This approach has previously been applied to transcriptomic data to remove the confounding effects of cell type changes on gene expression [30], but to our knowledge has not previously been applied to proteomic data. Deconvolution of cell type changes reduced the number of proteins with significantly different abundance levels between disease states (Fig. 3c). The number of proteins with different abundance levels between control and AD was reduced after deconvolution by approximately a factor of six, suggesting that most of the changes in protein abundance observed between control and AD are driven by changes in brain cell type. A similar reduction in abundance changes was observed between control and AsymAD after deconvolution. Notably, however, the number of proteins with unique changes in abundance between AsymAD and AD showed only a small reduction—from 290 to 263 proteins—after deconvolution for cell type, suggesting that most of the changes in protein abundance between AsymAD and AD are *not* driven primarily by changes in brain cell type. Instead, these changes may reflect a “biochemical phase” of AD [31]. There were slightly more proteins that were significantly lower in abundance compared to those that were higher in abundance in AD compared to AsymAD after cell type deconvolution (Additional file 14: Figure S7). Proteins that were elevated in AD compared to AsymAD included FABP7, SMOC1, and LTF, and tended to cluster in modules M4, M7, and M8. Those that were lower in AD compared to AsymAD included NPTX2, VGF, and GSTM1, and tended to cluster in modules M1, M2, and M3. Most of the modules in which the differentially abundant proteins between AsymAD and AD tended to cluster correlated with case status or AD pathology (Supplementary Data). GO network analysis of differentially abundant proteins between AsymAD and AD showed that many more protein ontologies became significant after cell type deconvolution, and existing ontologies identified in the unregressed analysis such as “cytoskeleton” became more significant (Fig. 3d and e). A GO network analysis of differentially abundant proteins between control and AsymAD cases before and after cell type deconvolution, representing protein changes early in the AD process, is provided in Additional file 15: Figure S8. In summary, these findings suggest that a majority of the differences in protein abundance between AsymAD and AD appear to be independent of simple brain cell type abundance changes, in contrast to the protein abundance differences between control and AsymAD and control and AD. Furthermore, proteins that change in abundance between AD and AsymAD are contained within modules that correlate with AD traits.

### RNA binding protein enrichment in the AD brain TMT proteomic network

After cell type deconvolution of protein abundance changes, we noted with keen interest the preservation of RNA binding proteins as hubs of differentially abundant proteins between control and AsymAD (Additional file 15: Figure S8), and between AsymAD and AD (Fig. 3e and Additional file 16: Figure S9). We have previously reported that aggregation of RNA binding proteins that are a part of the cellular pre-mRNA splicing machinery, especially the U1 small nuclear ribonucleoproteins (snRNPs) such as U1-70K, is an early event in AD pathogenesis [32]. The observation that RNA binding proteins emerged as hubs of differentially abundant proteins after cell type deconvolution prompted us to investigate whether certain TMT network modules were enriched in RNA binding proteins, and if so, whether these modules were associated with AD pathology. Upon examination of a number of different classes of RNA binding proteins, we found that modules 10, 15, 17, 18, 29, and 40 were significantly enriched with RNA binding proteins (Additional file 17: Figure S10A). Most of the RNA binding protein-enriched modules correlated with tau tangle burden as measured by Braak stage (Additional file 17: Figure S10B). Interestingly, our previous studies demonstrated that many of the U1 snRNPs colocalize with neurofibrillary tangles and paired helical filaments in AD brain [24, 32–34], and that accumulation of insoluble snRNPs correlates strongly with both amyloid and Tau pathology [32–37]. Collectively, these data support the relevance of this class of proteins to AD pathogenesis. The finding of strong RNA binding protein enrichment in certain modules within the AD TMT network led us to question whether these same modules contained more alternatively spliced proteins whose abundances may change as a consequence of AD pathophysiology. Changes in RNA splicing leading to the expression of different protein isoforms may be a useful indicator of AD pathology and cause downstream cellular and network dysfunction leading to cognitive decline.

### A Proteogenomic approach for the identification and quantification of alternative RNA splicing events in AD brain

**Fig. 4:**
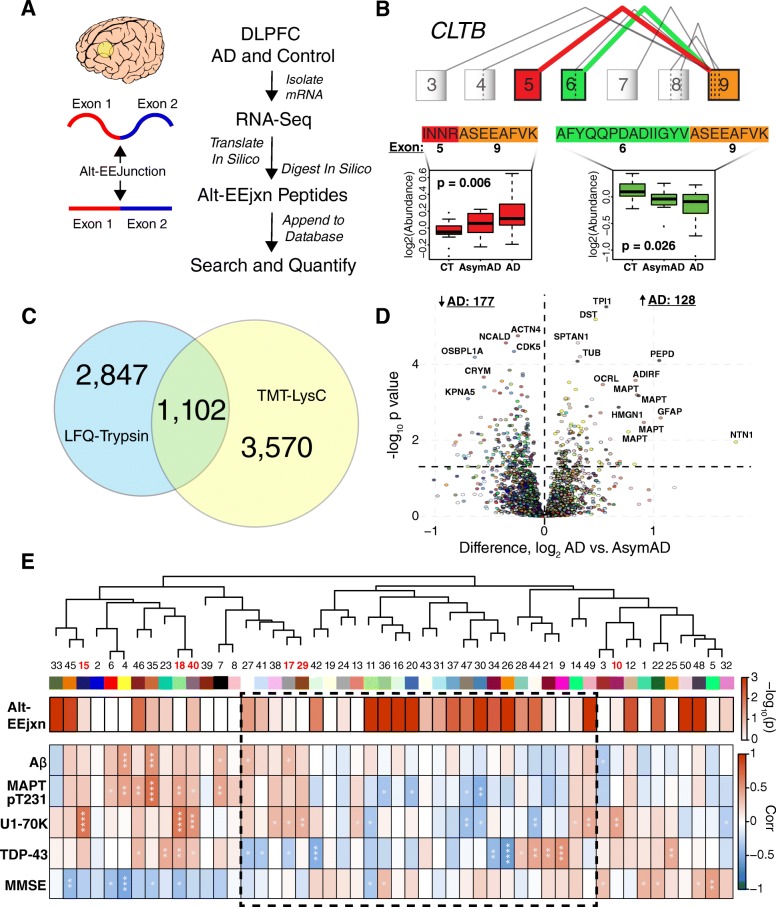
Alternative Splicing in AD. **a**–**e** Workflow for proteogenomic analysis of alternative splicing (**a**). RNA-Seq is performed on mRNA isolated from dorsolateral prefrontal cortex (DLPFC) control and AD brain. The mRNA sequences are then translated and digested with a given enzyme in silico to obtain peptide sequences. Peptide sequences that contain non-canonical exon-exon junctions (alt-EEjxns) are appended to the search database for peptide identification and subsequent quantification. **b** Illustration of alt-EEjxn peptide quantification for the clathrin light chain B protein (CLTB). An alternative splicing event between exons 5, 6, and 9 leads to the generation two alt-EEjxn peptides after enzymatic digestion. The levels of these alt-EEjxn peptides, each of which reflects a particular splicing “decision,” can be quantified across case groups. The exon numbering shown in panel (**b**) is based on the Gencode v19 exon annotation database, and includes RNA-derived junctions. **c** Venn diagram representing the number of alt-EEjxn peptides quantifiable by LFQ-trypsin or TMT-LysC pipelines in the BLSA cohort. A peptide was considered quantifiable if it had at least two measurements in at least two different case groups. The overlap between TMT-LysC and LFQ-trypsin represents quantifiable exon-exon junctions that were identified by both methods, even though the peptides that contain the junction may be different between the two methods. **d** Differential abundance of alt-EEjxns between AsymAD and AD, color-coded by the module to which each junction peptide is most highly correlated. The amino acid sequence of each alt-EEjxn peptide, and the module to which each alt-EEjxn peptide is most highly correlated, is provided in interactive HTML files for each case group comparison in Supplementary Data. **e** Enrichment of TMT-LysC alt-EEjxn peptides in TMT-LysC network modules. Each network module eigenprotein was correlated with molecular phenotypes obtained from the same tissue sample as the alt-EEjxn peptides, as well as with cognition as measured by the last MMSE score proximate to death. Modules within the dashed box are unique to the TMT network, similar to the depiction shown in Fig. 1a. Module numbers highlighted in red indicate modules enriched in RNA binding proteins as shown in Additional file 17: Figure S10. Significance of enrichment for alt-EEjxn peptides was calculated by Fisher exact test, and is shown by single color heat map of -log_10_ *p* value (increased red represents smaller *p* value). *P* values are corrected by Benjamini-Hochberg FDR. Module eigenprotein correlation with molecular species and cognition is shown by two-color heat map (red represents positive correlation, blue represents negative correlation). MMSE correlation was calculated by Spearman test. All other correlations are bicorrelation *rho*. Aβ, amyloid-β(17–28); MAPT pT231, microtubule associated protein tau peptide (VAVVR**pT**PPKSPSSAK) phosphorylated at threonine 231; U1-70K, U1 small nuclear ribonucleoprotein 70 kDa; TDP-43, TAR DNA-binding protein 43; MMSE, mini-mental state examination. *p < 0.05, **p < 0.01, ***p < 0.001, ****p < 0.0001

Before proceeding with a proteomic analysis of alternative RNA splicing in brain, we compared which mass spectrometry proteomic quantification pipeline—LFQ or TMT—would be most suitable for such an analysis. One aspect of the TMT quantification approach as implemented in our pipeline is that it uses LysC rather than trypsin enzymatic digestion prior to LC-MS/MS. LysC cleaves peptides only at lysine residues, whereas trypsin cleaves at both lysine and arginine residues. LysC therefore generates peptides that are, on average, of greater length than peptides generated after trypsin digestion [13]. We hypothesized that this increased peptide length may better capture exon-exon junction (EEjxn) sites generated through alternative splicing, and we therefore assessed whether the TMT-LysC approach allowed us to identify and quantify more alternatively spliced proteins than our previous LFQ-trypsin approach. A schematic of our approach for splicing analysis is shown in Fig. 4a, with details provided in Methods. We used RNA-seq data from control and AD DLPFC to generate a library of potentially translated polypeptides in silico, which we then digested in silico with LysC to generate proteolytic peptides that could be appended to standard databases for identification and quantification of alternative splicing events by mass spectrometry. An example of an alternative mRNA splicing decision quantified at the peptide level by TMT-MS shown in Fig. 4b. Using this proteogenomic approach, we were able to identify 5746 alternative exon-exon junction (alt-EEjxn) peptides in the LFQ-trypsin analysis, compared to 4830 alt-EEjxn peptides in the TMT-LysC analysis (Additional file 18: Figure S11). However, in the LFQ analysis over 1000 of the alt-EEjxn peptides were identified in only 1 out of 47 samples. When comparing the number of *quantifiable* alt-EEjxn peptides, the TMT approach was slightly better than LFQ over a range of data missingness thresholds (Additional file 18: Figure S11). However, because our TMT analysis was performed in batch with cases and controls present within the same batch, whereas LFQ is performed on individual cases, the difference in quantifiable alt-EEjxn peptides by *case status* between TMT and LFQ is larger than simply the difference in total quantifiable alt-EEjxn peptides. This difference is illustrated in Fig. 4c, and demonstrates the advantage of the TMT approach when quantifying proteins across multiple experimental groups. A summary comparison between the TMT-LysC and LFQ-trypsin approaches used here for alt-EEjxn peptide analysis is given in Additional file 5: Table S5, along with the custom databases from which the alt-EEjxn peptides were identified. As shown in Fig. 4c, LFQ-trypsin and TMT-LysC analyses identified and quantified largely separate subsets of alt-EEjxn peptides. Of those alt-EEjxns that were identified and quantified by both LFQ-trypsin and TMT-LysC approaches, the relative quantitative values correlated between the two approaches (Additional file 19: Figure S12), lending validity to the alt-EEjxn quantifications. From both of these analyses we were able to validate the occurrence of a number of alt-EEjxn splicing events in brain that have yet to be annotated in Swiss-Prot, and which heretofore have been predicted to exist only in the Trembl database, in our RNAseq data, or in both. For those alt-EEjxns that were identified in only the RNAseq data, trypsin and LysC also identified different subsets of junctions as reflected by the different GO ontologies for these alternatively spliced proteins, similar to the total alt-EEjxn quantifications (Additional file 20: Figure S13). A complete list and description of each alt-EEjxn peptide identified by LFQ-trypsin and TMT-LysC pipelines is given in Supplementary Data. In summary, we found that our TMT-LysC pipeline was superior to our previous LFQ-trypsin pipeline for quantitative analysis of alt-EEjxn splicing decisions in brain, and we therefore focused our subsequent analyses on alt-EEjxn peptides generated by LysC cleavage and quantified by TMT.

### Alternative splicing events associated with AD pathology and cognitive function

In order to examine which alt-EEjxn splicing events may be associated with progression of cognitive dysfunction from AsymAD to AD given the RNA binding protein abundance differences after cell type deconvolution between these two disease states, we performed a differential abundance analysis of alt-EEjxn peptides between AsymAD and AD. As shown in Fig. 4d, we found there were more alt-EEjxn peptides that were reduced in AD compared to AsymAD, similar to the total protein abundance differences between AD and AsymAD. Alt-EEjxns that were increased in AD were enriched in modules M4, M7, and M35, with M35 containing alt-EEjxns with the largest average change from AsymAD (Additional file 21: Figure S14). All of these modules were strongly glial in nature, with M35 a strongly astrocytic module. Tau had a number of alt-EEjxn peptides that were significantly increased in AD, and these mapped to the 3- and 4- microtubule binding domain repeat isoforms of the protein in this analysis because either isoform can be considered constitutively expressed in humans. Alt-EEjxns that were decreased in AD were most abundant in module M36—a module unique to the TMT network and without strong cell type character. We also analyzed specifically alt-EEjxns derived from the top twenty most significant common variant AD risk factor proteins identified from GWAS [12]. We observed alt-EEjxn peptides from a total of five of these proteins (Additional file 6: Table S6). Three of the five GWAS proteins had alt-EEjxns that were different in abundance by case status, and included BIN1, PTK2B, and FERMT2 (Additional file 7: Table S7). In summary, we identified a number of alternative splicing decisions at the protein level in brain that significantly change in AD, including in AD risk factor proteins identified from GWAS. Those that were increased in AD tended to cluster in astroglial modules.

**Fig. 5:**
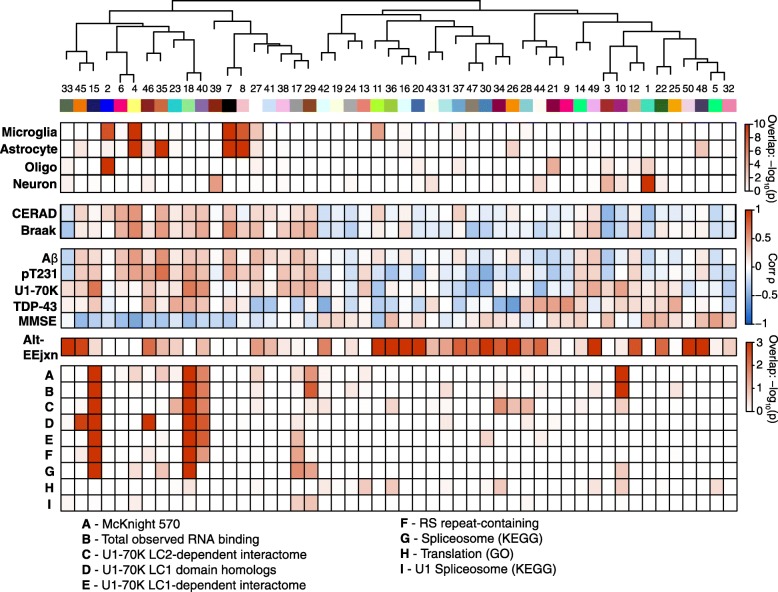
TMT-LysC AD Protein Network Summary. The overlap of proteins within each TMT-LysC module with cell type specific protein markers from microglia, astrocytes, oligodendrocytes (oligo), and neurons is shown by single color heat map (increased red represents increased overlap). Correlation between module eigenprotein and neuritic amyloid plaque burden (CERAD score), tau tangle burden (Braak stage), Aβ17–28, phosphorylated tau pT231 peptide (VAVVR**pT**PPKSPSSAK), U1-70K small nuclear ribonucleoprotein, TAR DNA-binding protein 43 (TDP-43), and last mini-mental state examination (MMSE) score prior to death, is shown by two-color heat map (red represents positive correlation, blue represents negative correlation). The overlap of alt-EEjxns and different classes of RNA binding proteins is shown by single color heat map. A, McKnight 570 refers to RNA binding proteins that are often found within RNA granules as described in [48]; B, Total Observed RNA binding refers to all RNA binding proteins commonly observed in our proteomic experiments; C, proteins that interact with the low complexity 2 (LC2) domain of the U1-70K small nuclear ribonucleoprotein 70 kDa (snRNP70) [54]; D, proteins that are homologous to U1-70K; E, proteins that interact with the LC1 or basic-acidic dipeptide (BAD) repeat domain of U1-70K [54]; F, low complexity arginine-serine (RS) repeat-containing proteins; G, proteins annotated as comprising the spliceosome complex in the Kyoto Encyclopedia of Genes and Genomes (KEGG); H, proteins annotated as involved in RNA translation by Gene Ontology (GO); I, proteins annotated in KEGG as belonging to the U1 spliceosome complex. Overlap was calculated by Fisher exact test. *P* values are corrected by Benjamini-Hochberg FDR. Bicorrelation was performed for CERAD, Braak, and molecular species. Spearman correlation was performed for MMSE. Detailed data for all correlations are provided in Supplementary Data

To extend our analysis of alt-EEjxns to the network level, we next assessed whether certain network modules were enriched for alt-EEjxns beyond those enriched only for differentially abundant alt-EEjxns, and if so, whether these modules were the same modules that were enriched in RNA binding proteins. To do so, we added the alt-EEjxn peptides identified by TMT-LysC to the TMT protein network modules with which they most highly correlated and tested whether a module contained more alt-EEjxn peptides than would be expected by chance. Interestingly, we found that there was little overlap between modules enriched in RNA binding proteins and those enriched in alt-EEjxn peptides (Fig. 4e), suggesting that pre-mRNA splicing changes in AD are not highly correlated with changes in the levels of RNA binding proteins, per se. Modules 4 and 7—glial modules enriched in AD risk factors from GWAS—did not contain more alt-EEjxn peptides than expected, but two other GWAS modules, M27 and M33, were enriched, with M33 being highly enriched. M33 contained snRNPC, a U1 snRNP involved in the spliceosome complex. Another highly enriched module, M15, also contained snRNPs snRNPB and snRNP70 (also known as U1-70K). We have previously shown that U1 snRNPs are a major component of the AD insoluble proteome [24, 32–34]. Modules that were most highly enriched with alt-EEjxn peptides clustered in the unique (i.e., protein module specific) region of the TMT network, with an especially enriched cluster in related modules 11, 36, 16, and 20. Modules 11 and 20 had corrected *p* values for enrichment of 4.1e^− 15^ and 1.7e^− 13^, respectively, and by GO analysis were most likely to be involved in regulation of immune system processes (data not shown). This region of the TMT network was not strongly associated with a particular cell type or strongly correlated with standard histopathological measures of AD. However, given that alternative splicing is a molecular event, we tested whether these modules might better correlate with molecular markers of AD pathology present within the same tissue sample rather than with the general pathological measures represented by CERAD score and Braak stage. For molecular correlation with tau, we correlated each module with the tau pT231 peptide (VAVVR**pT**PPKSPSSAK), which lies within the proline-rich region of tau and is separate from the microtubule-binding region that aggregates into neurofibrillary tangles. Phosphorylation of tau at T231 has been associated with AD [38], and levels of this peptide are moderately correlated with tau tangle burden (Additional file 10: Figure S3D). We also tested for correlation of each network module with Aβ17–28, U1-70K, and TDP-43, as well as with cognitive function as measured by last MMSE score prior to death. As shown in Fig. 4e, modules 11, 36, and 20 showed a significant correlation with either tau pT231, U1-70K, or cognition. Interestingly, the highly related modules 11 and 36 showed opposite correlation with cognition, with increases in M11 associated with worsened cognitive function and increases in M36 associated with improved cognitive function, suggesting that protein splicing changes may be highly specific in their relationships to different types of AD pathology and cognitive function. Module 35, which contained a number of differentially abundant alt-EEjxns, showed strong correlation to Aβ as well as to tau pT231, and correlated with worsened cognitive function. From the molecular analyses we also identified modules such as M18 that, although not enriched in alt-EEjxn peptides, were observed to be strongly enriched in RNA binding proteins (Additional file 22: Figure S15), were strongly correlated in a positive direction with tau pT231, U1-70K, and TDP-43, and were correlated in a negative direction with cognitive function. A summary of the TMT network findings that includes cell type character, correlation with histological and molecular markers of AD and cognition, and enrichment of RNA binding proteins and protein alt-EEjxns, is given in Fig. 5. Overall, we found that RNA binding proteins are enriched within specific network modules, and that these modules are generally positively correlated with AD pathology and negatively correlated with cognitive function. Modules that are enriched in alt-EEjxns do not overlap significantly with RNA binding protein modules, but many are located within the unique region of the TMT network and correlate with molecular markers of AD pathology. Some of these modules are strongly enriched with alt-EEjxns and correlate with cognitive function.

### Alternative splicing events associated with modules enriched in AD risk factor proteins

As a final separate approach to investigate alt-EEjxn splicing events that may be relevant to AD, we correlated each alt-EEjxn peptide with the module eigenprotein for those modules that were enriched in AD GWAS hits in the BLSA-TMT network (M4, M7, M27, and M33), and assessed whether the alt-EEjxn peptide was present in differential abundance among control, AsymAD, and AD brains. A list of the top ten most highly correlated alt-EEjxn peptides with these modules and their differential abundance by case status is given in Table 1. Most of the alt-EEjxn peptides that were highly correlated with the M4 and M7 modules were also present in differential abundance by case status, whereas few of the alt-EEjxn peptides that correlated highly to the M27 and M33 modules had different levels between control, AsymAD, and AD. In module M27, nearly all of the top ten most-highly correlated alt-EEjxn peptides were not present as full proteins within the module, with the exception of FERMT2, which has been identified as an AD risk factor protein. Proteins from which an alt-EEjxn peptide was identified that correlated to glial modules M27 and M33 are annotated as being involved in translation initiation, nucleic acid metabolism, protein folding chaperoning, and cytoskeleton organization, among other cellular functions. Interestingly, the most significant differential abundance in this list of highly correlated alt-EEjxn peptides was between AsymAD and AD, and was for an alt-EEjxn peptide derived from TPI1 (triosephosphate isomerase 1). TPI1 is involved in gluconeogenesis, but has also been identified as interacting directly with the Parkin protein, a ubiquitin protein ligase, and potentially affecting mitochondrial function [39]. Most of the alt-EEjxn peptides that highly correlated to the M27 module were from proteins that are involved in membrane scaffolding, endosomal transport and autophagy, as well as protein translation. We therefore identified a number of alternative splicing events that strongly correlated with network modules enriched in AD GWAS risk factor proteins, and some of these isoforms differed in abundance in AsymAD and AD.

**Table 1:**
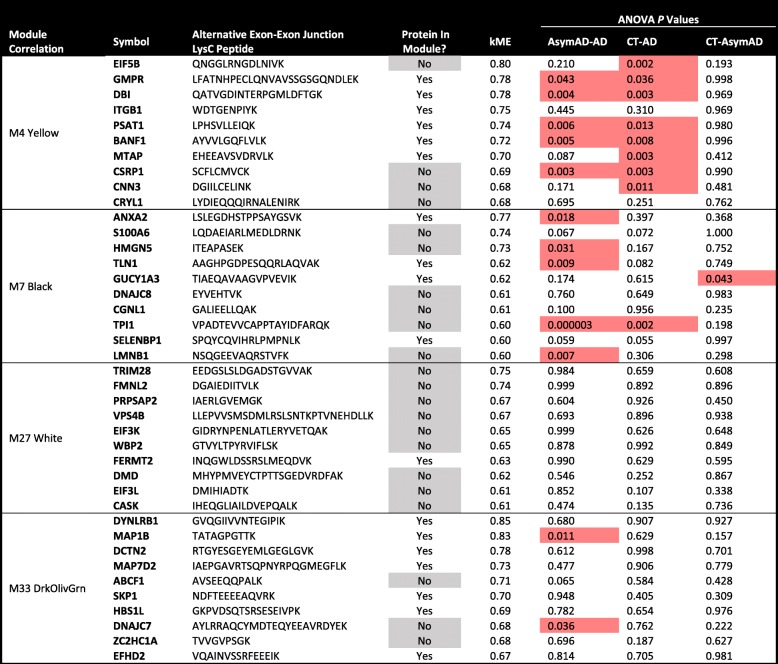
Correlation of alternatively spliced proteins with TMT protein network modules enriched in AD risk factors

The ten alt-EEjxn peptides with the strongest correlation to each protein network module enriched in AD risk factors are shown, including their differential abundance by case status. Proteins/peptides not present in the module to which they correlate are highlighted in gray. Proteins/peptides that are significantly different in abundance by case status are highlighted in red. ANOVA *p* values were adjusted for multiple comparisons by Tukey test. Detailed information for each alt-EEjxn peptide listed in the table, as well as for all identified alt-EEjxn peptides, is provided in Supplementary Data. AsymAD, asymptomatic AD; CT, control; kME, module eigenprotein correlation value

## Discussion

In this study we extended the depth of our proteomic network analysis of AD brain by approximately a factor of three using a new TMT-based analysis pipeline. The deeper protein coexpression network analysis revealed new protein modules that correlated with pathological measures of AD and were enriched in AD risk factors identified by GWAS. With this improved proteome coverage we were able to estimate the percentage of four different cell types within the brain and analyze how the abundance of these cell types changes in asymptomatic and symptomatic AD. We were also able to use these estimations of cell type changes to remove this potential confound from analysis of differential protein abundance changes in AsymAD and AD, and observed that most protein abundance changes between AsymAD and AD are not due to cell type changes. From this differential protein abundance analysis between AsymAD and AD we observed that RNA binding proteins were differentially altered between these two disease states, which led us to further analyze RNA binding proteins and alternatively spliced proteins within the TMT protein network. We found that RNA binding proteins clustered within specific network modules, and that some of these modules strongly correlated with molecular markers of AD and cognitive decline. Alternative exon-exon splicing events also tended to cluster within certain network modules, and some of these modules correlated with molecular markers of AD and cognitive decline. We identified a number of alt-EEjxn splicing events in AD GWAS risk factor proteins that were significantly altered in AD, as well as splicing events in other proteins that correlated with network modules enriched in AD risk factor proteins and were altered in AD.

The use of TMTs allowed us to perform an orthogonal offline prefractionation step prior to LC-MS/MS analysis while keeping MS analysis time within reasonable parameters through the ability to pool up to 11 tagged samples into a single batch prior to LC-MS/MS analysis. This approach has distinct advantages over standard “single-shot” LFQ analysis. Prefractionation significantly increases the depth of proteome coverage achievable by LC-MS/MS of complex tissues such as brain. TMTs also allow for relative protein measurements across multiple case groups within a single batch, minimizing the missing value problem for quantification across case groups. However, missing values are not eliminated in the TMT approach as it still relies on data-dependent acquisition techniques within each batch, and therefore not all batches contain the protein measurement of interest. Alternative approaches to protein quantification by mass spectrometry, such as data-independent acquisition [7, 40], may soon help to address the limitations on protein quantitation posed by data-dependent approaches. Nevertheless, we anticipate that further increases in the depth of proteome coverage in brain will be possible using a data-dependent TMT approach through advances in chromatography techniques and mass spectrometry instrumentation.

The increased depth of proteome coverage allowed us to build a protein coexpression network of AD brain that was significantly larger than our previous LFQ-based network [5]. It is notable that the TMT-LysC protein coexpression network nearly completely recapitulated the LFQ network we previously published, despite the fact that the TMT-LysC network was generated using an entirely different analysis pipeline with a new quantification approach and different mass spectrometry instrumentation. This finding lends validity to the previous LFQ network generated from the BLSA cohort, and by extension to LFQ-based networks of other cohorts we have analyzed ([41], unpublished data). Many of the new modules in the TMT network were not strongly associated with a particular cell type, indicating that most cell type specific modules were captured in the previous LFQ network. However, a few unique modules did show significant cell type character, including M27, which was largely microglial in cell type character, and by protein membership was the most unique module in the TMT-LysC network compared to the previous LFQ-trypsin network. This module was also enriched for AD GWAS risk factors and correlated with AD pathology, demonstrating that further increases in the depth of brain proteome coverage have the potential to reveal additional protein coexpression modules that are relevant to AD pathophysiology. In the TMT network we also observed a number of new modules that appeared to be anti-correlated with disease, potentially reflecting AD “resilience” modules. One such area of the network was the related cluster of modules M47 to M26. This cluster tended to be associated with improved cognition and lower levels of tau tangles, p-tau, U1-70K, and TDP-43. Further mechanistic investigation into the drivers of these protein coexpression changes may provide insights into factors that protect against AD.

From the cell type analysis, we found that astrocytes and microglia increase in relative proportion between AsymAD and AD, suggesting that immune system activation or dysfunction may be a primary driver of cognitive decline in the setting of AD pathology. Astrocytes and microglia also correlated more strongly with tau tangle burden than with amyloid-β plaque load, illustrating the connection between inflammation and tangle formation. The correlation between inflammation and tangle formation has also been noted in other tauopathies, such as frontotemporal dementia and chronic traumatic encephalopathy [42–45]. Interestingly, the neuron population decreased between control and AsymAD, with a further decrease between AsymAD and AD. It is not clear if synaptodendritic rarefaction may be driving this decreased measurement in cell population, or if it is actual neuron loss. Frank neuronal loss is often associated with late stages of the disease, and synapse loss in AD is thought to correlate with cognitive dysfunction. We expected the neuron population to correlated more strongly with tau tangle burden than with amyloid-β plaque burden given that tangle burden is more closely correlated with cortical atrophy and cognitive decline [46], but we observed that neurons correlated more strongly with amyloid plaques. Therefore, a discrepancy remains between the neuronal cell type data and disease state that warrants future investigation in a separate study cohort. We also noted an increase in oligodendrocytes in AsymAD, which is consistent with recent transcriptomic data suggesting alterations in oligodendrocyte and myelination biology in AD brain [30, 47]. Protein abundance differences between AsymAD and AD were largely preserved after adjustment for cell type changes, suggesting that perhaps these changes reflect a more “biochemical” phase of AD associated with cognitive dysfunction rather than a “cellular” phase of AD [31]. One cause of such biochemical changes may be changes in RNA binding proteins as identified in our GO network analysis, and as previously described by our group [32]. One of the most interesting RNA binding protein-enriched modules was M18. This module contained proteins often found in RNA granules [48], as well as proteins with low complexity domains such as U1-70K that bind to RNA and that have been associated with other neurodegenerative conditions such as frontotemporal dementia [49]. M18 was significantly correlated with phosphorylated tau, U1-70K, TDP-43, and cognitive decline, but did not contain an overabundance of proteins from any of the four cell types we tested, unlike modules such as M4 and M7 which we have previously found to be astroglial and strongly associated with AD [5]. One caveat regarding module correlation with cognitive decline in this analysis is that MMSE scores were skewed towards 30, suggesting that the cognitive time points captured from these individuals in the BLSA study were significantly removed from later disease stages. Future analyses using cohorts with more evenly distributed cognitive performance will be important to verify the cognitive associations reported here. Nevertheless, the MMSE-based cognitive correlations are likely correct in direction given their internal consistency with our previously published finding—validated here—that M4 and M7 correlate with progression from AsymAD to AD.

We developed an analytical pipeline to identify and quantify alternative exon-exon junctions at the protein level in brain. The databases we generated to identify alt-EEjxn peptides from brain were based on RNAseq data from relatively few control and AD brains from the University of Kentucky Brain Bank. However, most of the common alternatively spliced transcripts present in DLPFC control and AD brain were likely represented in this database. Adding RNAseq data from additional brains would perhaps uncover more rare local splicing variations, and will be a focus of future work. In our analysis of alt-EEjxns we observed a number of local splice variants that have not yet been documented to exist at the protein level in any human tissue. Because brain contains a large number of alternatively spliced proteins [50], we consider it likely that deeper characterization of protein splice variants in brain will uncover even more local splice variations that are translated into protein, with some being potentially relevant to disease. Our comparison between LFQ-trypsin and TMT-LysC analytical pipelines found that the TMT-LysC approach was slightly superior to LFQ-trypsin for quantification of alt-EEjxns, and even better when quantifying alt-EEjxns across case groups. However, for simply validating the existence of a particular alt-EEjxn at the protein level, LFQ-trypsin was superior to TMT-LysC. This is likely the case because trypsin digestion is more efficient than LysC and provides deeper coverage of the proteome, despite the fact that the number of peptides containing exon-exon splice junctions are reduced with trypsin digestion due to the overabundance of basic amino acid residues at splice junctions [51]. A significantly deeper “bottom-up” analysis of local splicing variation at the proteomic level will likely require multiple and orthogonal enzymatic digestion approaches. As a case-in-point, we observed only 4 alt-EEjxns at the protein level out of a possible 74 alt-EEjxns at the mRNA transcript level in PICALM. It is unclear how many of these local splice variants are translated into protein rather than undergo nonsense-mediated decay, but it seems likely based on abundant steady state transcript levels that a majority of these splice isoforms are translated into protein. The use of an orthogonal digestion approach for better splice junction coverage is also supported by the observation that trypsin and LysC identified largely separate subsets of alt-EEjxns, both for junctions currently annotated in protein databases and for those observed only in RNAseq or expressed sequence tag data. It should be noted that our quantitative analysis of alt-EEjxns was necessarily limited to the peptide level, and therefore the analysis is best considered to represent quantification of alternative splicing “decisions” in brain, whereby many separate splicing decisions may contribute in a combinatorial fashion to the generation of different protein isoforms.

From the alt-EEjxns we identified, those that were elevated in AD compared to AsymAD tended to cluster into modules that were microglial or astrocytic in nature. It is possible that the increase in these cell types in AD lead to a relative increase in alt-EEjxns that are otherwise translated at a low baseline level, and development of an algorithm to potentially exclude this effect, similar to cell type deconvolution for total protein levels, would be a welcome advance for alt-EEjxn analysis. Alternatively, splicing decisions may change systematically and may also underlie phenotype changes among the astroglial population of cells in brain. A future analysis to probe the extent of splice decision “switching” in AD, whereby an alt-EEjxn is favored at the expense of the canonical junction, would also be informative. A number of alt-EEjxns in AD GWAS risk factor proteins were elevated in AD, including in BIN1 and PTK2B. The functional relevance of these alternative exon-exon splicing decisions in these and other AD risk factor proteins remains to be determined. We found that alt-EEjxns at a global level tended to cluster into the unique area of the TMT-LysC network, but did not significantly overlap with modules enriched in RNA binding proteins. Although we have previously observed that snRNP alterations are associated with deficits in RNA splicing in AD brain [24, 32]—a finding recently confirmed by others [52]—the fact that there was little overlap with RNA binding proteins at the network level suggests that abundance levels of RNA binding proteins do not correlate directly with levels of alternative splicing. Rather, it is likely that only certain types of RNA binding proteins directly affect alternative splicing decisions. We assessed for protein components of the U1 spliceosome complex in our enrichment analysis, but we did not find strong enrichment of these proteins in the network. This may be due to the fact that U1 spliceosome proteins undergo a dramatic shift in solubility in AD brain, and aggregate in close proximity to neurofibrillary tangles [24, 32–34]. Module 29 contained five snRNPs and was annotated as being involved in mRNA splicing by GO analysis, but did not show enrichment of alt-EEjxn peptides. The relationship between RNA protein abundance and alternative splicing remains an area for future investigation.

## Conclusions

We developed a TMT-based quantification pipeline for proteomic analysis of brain tissue that significantly increased our depth of proteome coverage of control and AD brain and led to additional insights into the protein changes that characterize AD pathophysiology, including changes in RNA splicing. Future advances in alternative protein isoform analysis by mass spectrometry will undoubtedly shed further light on this “dark matter” of the proteome and its role in AD.

## Additional files

Additional file 1: **Table S1.** Sample List. AD, Alzheimer’s disease; AS, asymptomatic Alzheimer’s disease; CT, control; MCI, mild cognitive impairment; CERAD, Consortium to Establish a Registry for Alzheimer’s Disease amyloid-β plaque load score; Braak, Braak stage for tau tangle burden; PMI, post-mortem interval; ApoE, apolipoprotein E isoform genotype; MMSE, Mini-Mental State Examination; NA, not available. MCI cases were not used in the final analysis. (DOCX 45 kb)

Additional file 2: **Table S2.** Case Characteristics. Values shown are means ± SD. AD, Alzheimer’s disease; AsymAD, asymptomatic Alzheimer’s disease; MCI, mild cognitive impairment; CERAD, Consortium to Establish a Registry for Alzheimer’s Disease amyloid-β plaque load score; Braak, Braak stage for tau tangle burden; PMI, post-mortem interval; ApoE, apolipoprotein E isoform genotype. (DOCX 30 kb)

Additional file 3: **Table S3.** TMT Experimental Design. MCI cases (*n* = 11) were removed after batch correction. The final cohort used for quantification was *n* = 47 DLPFC non-MCI samples. TMT, tandem mass tag; GIS, global internal standard; BLSA, Baltimore Longitudinal Study of Aging; AD, Alzheimer’s disease; AsymAD, asymptomatic Alzheimer’s disease; MCI, mild cognitive impairment; DLPFC, dorsolateral prefrontal cortex. (PDF 888 kb)

Additional file 4: **Table S4.** List of Biological Terms for GO Network in **Figure S8**. GO, gene ontology; UP, UniProt; KEGG, Kyoto Encyclopedia of Genes and Genomes; SMART, Simple Modular Architecture Research Tool; FDR, false discovery rate. (DOCX 35 kb)

Additional file 5: **Table S5.** Number of Alternative Exon-Exon Junction Peptides Identified by TMT-LysC and LFQ-trypsin Approaches. The number of alt-EEjxn peptides identified by matching to the listed databases (Swiss-Prot, Trembl, or RNAseq) is shown, along with the number of quantifiable alt-EEjxn peptides. A peptide was considered quantifiable in this analysis if it had a minimum of 2 measurements in at least 2 different case groups. RNAseq data from control and AD patient brains (*n* = 6) were used to generate the RNAseq alt-EEjxn peptide database, as described in Methods. (DOCX 28 kb)

Additional file 6: **Table S6.** Number of Alternative Exon-Exon Junctions in AD Risk Factor Proteins. From the twenty proteins identified as risk factors for AD by GWAS at genome-wide significance [12], five had alt-EEjxn peptides that were observed and quantifiable in the BLSA-TMT analysis (observed). The number of observed and quantifiable alt-EEjxn peptides for each of these five proteins was a subset of the total number of alt-EEjxn peptides predicted to exist after LysC digestion (peptide database). This number was a further subset of the total number of alt-EEjxns observed for each of the five proteins from RNAseq data (transcript level). For details on generation of the peptide database and transcript level numbers, see Methods. (DOCX 28 kb)

Additional file 7: **Table S7.** AD Risk Factor Protein Alternative Exon-Exon Junction Peptides Significantly Altered by Case Status. Of the five AD risk factor proteins that had quantifiable alt-EEjxn peptides in the BLSA-TMT analysis, three had alt-EEjxn peptides that were significantly, or nearly significantly, different in abundance by case status. For PTK2B 20038, module eigenprotein correlation was not performed due to the number of missing values for quantitation (≥25). A complete description of each alt-EEjxn peptide is provided in Supplementary Data. ME bicor, module eigenprotein bicorrelation; kME, correlation value to the module eigenprotein; AD, Alzheimer’s disease; AsymAD, asymptomatic Alzheimer’s disease. (DOCX 29 kb)

Additional file 8: **Figure S1.** SDS-PAGE of Brain Homogenates. Dorsolateral prefrontal cortex (DLPFC) brain tissue homogenates from cases shown in **Table S1** were analyzed by SDS-PAGE to assess sample integrity prior to TMT labeling and mass spectrometry analysis. Gels were stained with Coomassie Blue to visualize protein. AD, Alzheimer’s disease; AS, asymptomatic Alzheimer’s disease; CT, control; MCI, mild cognitive impairment. (PDF 22000 kb)

Additional file 9: **Figure S2.** Protein Quantitation in TMT-LysC and LFQ-trypsin Analyses. The relationship between the number of quantifiable proteins at a given threshold of missing values in the 47 brain samples from the BLSA cohort for TMT-LysC and LFQ-trypsin analyses is shown. The point at 23 samples and 6533 proteins represents the threshold used for the TMT-LysC analysis pipeline in this study. This point falls slightly below the TMT curve because 11 MCI samples were included in the TMT analysis workflow, for a total of 58 samples, but were later dropped from the analysis (see Methods). The increased number of samples when including the 11 MCI cases slightly reduced the number of quantifiable proteins at the ~ 50% missing value threshold. (PDF 79 kb)

Additional file 10: **Figure S3.** Measurement of Aβ and Tau. (A-D) Total amyloid-β levels were measured using the Aβ17–28 peptide fragment (A, left). Aβ levels were quantified in the TMT-LysC analysis across case groups using SPS-MS3 reporter ions to Aβ17–28 (A, right). (B) The Aβ17–28 peptide was quantified by LFQ extracted ion current intensity [5], and compared to quantification using TMTs with SPS-MS3 reporter ion relative intensities normalized to the global internal standard (GIS). (C) The Aβ17–28 peptide was quantified by TMT across the 47 cases and correlated to CERAD score, a histopathological measure of neuritic amyloid plaque burden. (D) The tau pT231 peptide (VAVVR**pT**PPKSPSSAK), which is derived from the proline-rich domain, was quantified by TMT and correlated with Braak stage, a histological staging system for tau neurofibrillary tangle burden. (PDF 299 kb)

Additional file 11: **Figure S4.** TMT Network Modules Associated with Disease State or AD Pathology. (A-F) TMT network modules that were enriched in astrocyte or microglial proteins (A), neuronal proteins (B), ‘de nove’ post-translational protein folding machinery (C), mitochondrial proteins (D), nucleosomal proteins (E), or RNA-associated proteins (F), and which also changed with disease state or were correlated to AD pathology are shown, along with the top six hub proteins for each module. The full list of modules and pathological correlations for each module is provided in Supplementary Data. Eigenprotein differences by disease state were assessed by one-way ANOVA. (PDF 180 kb)

Additional file 12: **Figure S5.** TMT Protein Network Modules Enriched for AD Risk Factors. Graphical representation of the correlation relationships among TMT network module proteins for the four modules identified to contain enrichment of AD risk factors from GWAS, along with the relationship of each module to case status, neuritic amyloid plaque load (CERAD score), and tau tangle burden (Braak stage). Proteins identified by GWAS as AD risk factors are highlighted in red. Only the top 100 proteins by kME value are shown for the M4 yellow (257 total proteins) and M7 black (162 total proteins) modules. (PDF 462 kb)

Additional file 13: **Figure S6.** Cell Type Population Changes Associated with AD and Correlation with Amyloid and Tau Pathology. The abundance of cell type-specific protein markers of astrocytes, microglia, neurons, and oligodendrocytes (oligos) was used to calculate the percentage of each cell type in control, asymptomatic AD (AsymAD), and AD brain tissue (see Methods). Percentage cell type was then correlated with the degree of neuritic amyloid plaque pathology (CERAD Amyloid Score) and tau tangle burden (Braak Stage) across all brains. (PDF 141 kb)

Additional file 14: **Figure S7.** Differential Protein Abundance between AD and AsymAD. Proteins that were significantly increased (160) or decreased (190) in AD compared to AsymAD, color-coded by TMT network module membership, are shown. The horizontal dotted line represents *p* = 0.05. Interactive plots for AD vs. AsymAD, AD vs. control, and AsymAD vs. control differential protein abundance are provided in Supplementary Data. (PDF 10400 kb)

Additional file 15: **Figure S8.** GO Network Analysis for Differential Protein Abundance Between AsymAD and Control. (A, B) Proteins with significant differences in abundance between asymptomatic AD and control before (A) after (B) cell type deconvolution were analyzed by gene ontology (GO) network analysis. Only two nodes were significant before cell type deconvolution, and no nodes were significant after cell type deconvolution, by false discovery rate (FDR) Q value statistic. Therefore, significance values are represented by the less stringent uncorrected *p* value. RNA binding protein nodes are highlighted in green. (PDF 228 kb)

Additional file 16: **Figure S9.** GO Network Analysis for Differential Protein Abundance Between AD and AsymAD. Proteins with significant differences in abundance between asymptomatic AD and AD after cell type deconvolution were analyzed by gene ontology (GO) network analysis. A complete list of biological terms that correspond to each node in the network, along with the source for the term and the false discovery rate (FDR) Q value statistic, is given in **Table S4**. (PDF 244 kb)

Additional file 17: **Figure S10.** RNA Binding Protein Enrichment in TMT Network Modules and Correlation with AD Pathology. (A, B) Overlap of different groups of RNA binding proteins within TMT network modules (A). Significance of overlap was calculated by Fisher exact test, and is shown by single color heat map of -log_10_ *p* value (increased red represents smaller *p* value and increased overlap). *P* values are corrected by Benjamini-Hochberg FDR. A, McKnight 570 refers to RNA binding proteins that are often found within RNA granules as described in [48]; B, Total Observed RNA binding refers to all RNA binding proteins commonly observed in our proteomic experiments; C, proteins that interact with the low complexity 2 (LC2) domain of the U1-70K small nuclear ribonucleoprotein 70 kDa (snRNP70) [54]; D, proteins that are homologous to U1-70K; E, proteins that interact with the LC1 or basic-acidic dipeptide (BAD) repeat domain of U1-70K [54]; F, low complexity arginine-serine (RS) repeat-containing proteins; G, proteins annotated as comprising the spliceosome complex in the Kyoto Encyclopedia of Genes and Genomes (KEGG); H, proteins annotated as involved in RNA translation by Gene Ontology (GO); I, proteins annotated in KEGG as belonging to the U1 spliceosome complex. (B) The six modules most enriched in RNA binding proteins (M15, M18, M40, M17, M29, and M10) were assessed for change by case group and correlation with tau tangle burden (Braak stage). Four out of the six modules significantly correlated with Braak stage. Correlation was performed by the bicorrelation function as implemented in R. CT, control; AsymAD, asymptomatic Alzheimer’s disease; AD, Alzheimer’s disease. (PDF 254 kb)

Additional file 18: **Figure S11.** Alternative Exon-Exon Junction Peptide Quantitation in TMT-LysC and LFQ-trypsin Analyses. The relationship between the number of quantifiable alternative exon-exon junction (alt-EEjxn) peptides at a given threshold of missing values in the 47 brain samples from the BLSA cohort for TMT-LysC and LFQ-trypsin analyses is shown, without regard to case group. Also shown is the number of alt-EEjxn peptides quantified by TMT-LysC that had a LFQ-trypsin cognate peptide, as well as the number of alt-EEjxn peptides quantified by LFQ-trypsin that had a cognate TMT-LysC peptide. The point at 23 samples represents the 50% missingness threshold. (PDF 89 kb)

Additional file 19: **Figure S12.** Correlation Between Alternative Exon-Exon Junctions Quantified by TMT-LysC and LFQ-trypsin Analyses. Alternative exon-exon junctions (alt-EEjxns) that were identified and quantified in both TMT-LysC and LFQ-trypsin analyses (*n* = 1202 alt-EEjxns) and which had no missing values across the 47 BLSA cases were matched case-to-case, and the log(2) normalized intensity measurements for each alt-EEjxn were correlated between the two quantification approaches. Note that the peptide containing the alt-EEjxn is not necessarily identical between TMT-LysC and LFQ-trypsin analyses. When the correlation is restricted to identical alt-EEjxn peptides (*n* = 728), the strength of correlation increases only slightly (*r* = 0.6) (data not shown). (PDF 15000 kb)

Additional file 20: **Figure S13.** GO Analysis of Alternative Exon-Exon Junction Peptides Unique to the RNAseq Database. Alternative exon-exon junction (alt-EEjxn) peptides that were identified by LFQ-trypsin or TMT-LysC approaches from the RNAseq data only were analyzed by gene ontology (GO), which showed that the alternatively spliced proteins identified by the two approaches in the RNAseq data were largely unique. (PDF 660 kb)

Additional file 21: **Figure S14.** Differential Abundance of Alternative Exon-Exon Junction Peptides by TMT Network Module. For case group comparisons AD vs. AsymAD (top), AD vs. control (middle), and AsymAD vs. control (bottom), the fraction of alternative exon-exon junction (alt-EEjxn) peptides within each network module that were significantly different between the two case groups was plotted by bar graph, with each bar color coded according to the average log2 difference of the alt-EEjxn peptides in each direction (increased or decreased). The arrows in the AD vs. AsymAD comparison highlight modules that showed an increase in the fraction of alt-EEjxns or an increase in the magnitude of differential abundance, or both, compared to AD vs. control. (PDF 718 kb)

Additional file 22: **Figure S15.** Enrichment of RNA Binding Proteins in TMT Network Module 18. Graphical representation of the correlation relationships among proteins for lightgreen module M18, with proteins centrally located representing those most highly correlated with other proteins in the module. Proteins annotated as RNA binding proteins in geneontology.org are highlighted in yellow. (PDF 177 kb)

## Abbreviations

AD: Alzheimer’s disease
alt-EEjxn: alternative exon-exon junction
AsymAD: Asymptomatic AD
Aβ: Amyloid-β
BIN1: Myc box-dependent-interacting protein 1
BLSA: Baltimore Longitudinal Study of Aging
CERAD: Consortium to Establish a Registry for Alzheimer’s Disease
DLPFC: Dorsolateral prefrontal cortex
ERLIC: Electrostatic repulsion—hydrophilic interaction chromatography
FABP7: Fatty acid-binding protein, brain
FERMT2: Fermitin family homolog 2
GIS: Global internal standard
GO: Gene ontology
GSTM1: Glutathione S-transferase mu 1
GWAS: Genome-wide association study
HPLC: High pressure liquid chromatography
IGAP: International Genetics of Alzheimer’s Project
LC-MS/MS: Liquid chromatography tandem mass spectrometry
LFQ: Label-free quantification
LTF: Lactotransferrin
MCI: Mild cognitive impairment
MMSE: Mini-mental status examination
NPTX2: Neuronal pentraxin-2
PICALM: Phosphatidylinositol-binding clathrin assembly protein
p-tau: phospho-tau
PTK2B: Protein-tyrosine kinase 2-beta
SMOC1: SPARC-related modular calcium-binding protein 1
snRNP: small nuclear ribonucleoprotein
SPS-MS3: Synchronous precursor selection-MS3
TDP-43: TAR DNA-binding protein 43
TMEM106B: Transmembrane protein 106B
TMT: Tandem mass tag
TMT-MS: Tandem mass tag mass spectrometry
TPI1: Triosephosphate isomerase 1
U1-70K: U1 small nuclear ribonuclearprotein 70 kDa
VGF: Neurosecretory protein VGF
WGCNA: Weighted gene co-expression network analysis
WPCNA: Weighted protein co-expression network analysis
