# Supplementary material for: Deep proteomic network analysis of Alzheimer’s disease brain reveals alterations in RNA binding proteins and RNA splicing associated with disease
Source: Mol Neurodegener. 2018 Oct 4;13:52. doi: 10.1186/s13024-018-0282-4 (PMC6172707; doi:10.1186/s13024-018-0282-4)
Supplement: Supplementary file 1 — Table S1. Sample List. AD, Alzheimer’s disease; AS, asymptomatic Alzheimer’s disease; CT, control; MCI, mild cognitive impairment; CERAD, Consortium to Establish a Registry for Alzheimer’s Disease amyloid-β plaque load score; Braak, Braak stage for tau tangle burden; PMI, post-mortem interval; ApoE, apolipoprotein E isoform genotype; MMSE, Mini-Mental State Examination; NA, not available. MCI cases were not used in the final analysis. (DOCX 45 kb) [file 13024_2018_282_MOESM1_ESM.docx]

**Additional file 1**

| Case ID | CERAD | Braak | Age | Sex | PMI (h) | ApoE | MMSE |
| --- | --- | --- | --- | --- | --- | --- | --- |
| AD_1284 | 3 | 4 | 83 | F | 18 | 3/3 | 9 |
| AD_1407 | 3 | 5 | 81 | M | 7 | 3/3 | 19 |
| AD_1430 | 2 | 5 | 96 | M | 14 | 3/3 | 11 |
| AD_1556 | 3 | 6 | 86 | M | 8 | 4/4 | 27 |
| AD_1649 | 3 | 4 | 98 | M | 20 | 3/3 | NA |
| AD_1712 | 3 | 4 | 92 | M | 7 | 3/3 | 24 |
| AD_1735 | 3 | 6 | 86 | M | 15 | 3/4 | 26 |
| AD_1839 | 3 | 5 | 88 | M | 14 | 3/3 | 28 |
| AD_1875 | 3 | 6 | 82 | F | 6 | 2/3 | 22 |
| AD_1921 | 3 | 6 | 72 | F | 10 | 3/3 | 8 |
| AD_1973 | 3 | 6 | 92 | M | 12 | 3/4 | 22 |
| AD_1984 | 3 | 6 | 92 | F | 19 | 3/4 | 11 |
| AD_2004 | 3 | 6 | 82 | M | 23 | 2/3 | 23 |
| AD_2023 | 2 | 4 | 96 | M | 19 | 3/3 | 25 |
| AD_2028 | 3 | 6 | 83 | F | 11 | 3/4 | 28 |
| AD_2032 | 3 | 6 | 94 | F | 17.5 | 2/3 | 29 |
| AD_2157 | 3 | 6 | 91 | F | 18 | 3/4 | 22 |
| AD_2184 | 3 | 6 | 62 | F | 14.5 | 3/3 | NA |
| AD_2226 | 3 | 6 | 90 | F | 13 | 3/3 | 30 |
| AD_2274 | 3 | 5 | 80 | F | 19 | 3/4 | 27 |
| AS_0803 | 2 | 2 | 83 | M | 5 | 3/3 | NA |
| AS_1479 | 2 | 3 | 83 | F | 23 | 3/3 | 29 |
| AS_1591 | 1 | 3 | 94 | M | 16 | 3/4 | 30 |
| AS_1720 | 2 | 4 | 95 | F | 2 | 3/3 | 30 |
| AS_1734 | 2 | 4 | 92 | F | 12 | 3/3 | 29 |
| AS_1843 | 2 | 4 | 92 | M | 18 | 2/3 | 30 |
| AS_1867 | 2 | 4 | 94 | M | 5 | 3/3 | 28 |
| AS_1924 | 2 | 4 | 83 | M | 6 | 3/3 | 30 |
| AS_2011 | 3 | 4 | 71 | M | 15.5 | 3/4 | 30 |
| AS_2037 | 3 | 4 | 75 | M | 24 | 3/4 | 29 |
| AS_2069 | 2 | 4 | 92 | F | 18 | 3/3 | 28 |
| AS_2190 | 2 | 6 | 92 | M | 8.5 | 3/3 | 28 |
| AS_2316 | 2 | 3 | 96 | M | 5.5 | 3/3 | 29 |
| AS_2342 | 2 | 3 | 96 | M | 10.5 | 3/3 | 25 |
| CT_0827 | 0 | 1 | 79 | M | 10 | 3/3 | NA |
| CT_1036 | 0 | 2 | 81 | M | 20 | 3/3 | 30 |
| CT_1312 | 1 | 2 | 80 | M | 22 | 3/3 | 29 |
| CT_1313 | 0 | 2 | 92 | F | 14 | 3/3 | 21 |
| CT_1471 | 0 | 2 | 87 | M | 14 | 2/3 | 29 |
| CT_1517 | 1 | 2 | 71 | F | 16 | 4/4 | 28 |
| CT_1672 | 0 | 2 | 84 | M | 17 | 2/3 | 28 |
| CT_2020 | 0 | 3 | 82 | M | 14.5 | 2/3 | 30 |
| CT_2021 | 0 | 4 | 99 | M | 24 | 3/3 | 28 |
| CT_2027 | 0 | 4 | 86 | M | 7 | 2/3 | 27 |
| CT_2066 | 0 | 3 | 95 | M | 17 | 2/3 | 26 |
| CT_2151 | 0 | 2 | 72 | M | 10 | 3/3 | 29 |
| CT_2228 | 0 | 1 | 64 | M | 28 | 3/3 | NA |
| CT_2317 | 0 | 1 | 65 | F | 23 | 3/3 | 29 |
| MCI_1273 | 0 | 2 | 68 | M | 10 | 3/3 |  |
| MCI_1341 | 2 | 3 | 89 | F | 20 | 3/3 |  |
| MCI_1372 | 1 | 1 | 96 | M | 35 | 3/3 |  |
| MCI_1543 | 2 | 3 | 87 | M | 20 | 3/3 |  |
| MCI_1548 | 1 | 3 | 90 | M | 14 | 2/3 |  |
| MCI_1603 | 1 | 4 | 89 | M | 16 | 3/3 |  |
| MCI_1669 | 1 | 2 | 90 | M | 14 | 3/3 |  |
| MCI_1790 | 1 | 3 | 88 | F | 14 | 3/3 |  |
| MCI_1805 | 2 | 4 | 82 | M | 5.5 | 3/3 |  |
| MCI_1969 | 2 | 4 | 78 | M | 4 | 3/4 |  |
| MCI_2000 | 2 | 4 | 101 | F | 25 | 3/3 |  |

**Table S1**
