# Supplementary material for: Deep proteomic network analysis of Alzheimer’s disease brain reveals alterations in RNA binding proteins and RNA splicing associated with disease
Source: Mol Neurodegener. 2018 Oct 4;13:52. doi: 10.1186/s13024-018-0282-4 (PMC6172707; doi:10.1186/s13024-018-0282-4)
Supplement: Supplementary file 2 — Table S2. Case Characteristics. Values shown are means ± SD. AD, Alzheimer’s disease; AsymAD, asymptomatic Alzheimer’s disease; MCI, mild cognitive impairment; CERAD, Consortium to Establish a Registry for Alzheimer’s Disease amyloid-β plaque load score; Braak, Braak stage for tau tangle burden; PMI, post-mortem interval; ApoE, apolipoprotein E isoform genotype. (DOCX 30 kb) [file 13024_2018_282_MOESM2_ESM.docx]

| Case Group | CERAD | Braak | Age | Sex (%M) | PMI (h) | ApoE (ε/ε) | | | |
| --- | --- | --- | --- | --- | --- | --- | --- | --- | --- |
|  |  |  |  |  |  | 2/3 | 3/3 | 3/4 | 4/4 |
| Control | 0.1±0.4 | 2.2±1.0 | 81.2±10.5 | 79 | 16.9±6.0 | 5 | 8 | 0 | 1 |
| AsymAD | 2.1±0.5 | 3.7±0.9 | 88.4±8.1 | 71 | 12.1±7.1 | 1 | 10 | 3 | 0 |
| AD | 2.9±0.3 | 5.5±0.8 | 86.5±8.9 | 53 | 14.1±5.0 | 3 | 10 | 6 | 1 |
| MCI | 1.4±0.7 | 3.0±1.0 | 87.1±8.8 | 73 | 16.1±8.8 | 1 | 9 | 1 | 0 |

**Table S2**
