# Supplementary material for: Deep proteomic network analysis of Alzheimer’s disease brain reveals alterations in RNA binding proteins and RNA splicing associated with disease
Source: Mol Neurodegener. 2018 Oct 4;13:52. doi: 10.1186/s13024-018-0282-4 (PMC6172707; doi:10.1186/s13024-018-0282-4)
Supplement: Supplementary file 3 — Table S3. TMT Experimental Design. MCI cases (n = 11) were removed after batch correction. The final cohort used for quantification was n = 47 DLPFC non-MCI samples. TMT, tandem mass tag; GIS, global internal standard; BLSA, Baltimore Longitudinal Study of Aging; AD, Alzheimer’s disease; AsymAD, asymptomatic Alzheimer’s disease; MCI, mild cognitive impairment; DLPFC, dorsolateral prefrontal cortex. (PDF 888 kb) [file 13024_2018_282_MOESM3_ESM.pdf]

Batch 1

Case#	Case Type	TMT10-
	BLSA GIS	126
1407	AD	127N
1430	AD	127C
1273	MCI	128N
1341	MCI	128C
0803	AsymAD	129N
2342	AsymAD	129C
0827	CONTROL	130N
1036	CONTROL	130C
	EMORY GIS	131

Batch 2

Case#	Case Type	TMT10-
	BLSA GIS	126
1649	AD	127N
1712	AD	127C
1372	MCI	128N
1543	MCI	128C
1479	AsymAD	129N
1591	AsymAD	129C
1312	CONTROL	130N
1313	CONTROL	130C
	EMORY GIS	131

Batch 3

Case#	Case Type	TMT10-
	BLSA GIS	126
1735	AD	127N
1839	AD	127C
1875	AD	128N
1548	MCI	128C
1720	AsymAD	129N
1734	AsymAD	129C
1471	CONTROL	130N
1517	CONTROL	130C
	EMORY GIS	131

Batch 4

Case#	Case Type	TMT10-
	BLSA GIS	126
1973	AD	127N
1984	AD	127C
1603	MCI	128N
1669	MCI	128C
1843	AsymAD	129N
1867	AsymAD	129C
1672	CONTROL	130N
2020	CONTROL	130C
	EMORY GIS	131

Batch 5

Case#	Case Type	TMT10-
	BLSA	126
2004	AD	127N
2023	AD	127C
2028	AD	128N
1790	MCI	128C
1924	AsymAD	129N
2011	AsymAD	129C
2021	CONTROL	130N
2027	CONTROL	130C
	EMORY GIS	131

Batch 6

Case#	Case Type	TMT10-
	BLSA GIS	126
2032	AD	128N
2157	AD	128C
1805	MCI	130N
1969	MCI	130C
2037	AsymAD	129N
2069	AsymAD	129C
2066	CONTROL	127N
2151	CONTROL	127C
	EMORY GIS	131

Batch 7

Case#	Case Type	TMT10-
	BLSA GIS	126
2184	AD	127N
2226	AD	127C
2274	AD	128N
2000	MCI	128C
2190	AsymAD	129N
2316	AsymAD	129C
2228	CONTROL	130N
2317	CONTROL	130C
	EMORY GIS	131

Batch 8

Case#	Case Type	TMT10-
	BLSA GIS	126
	BLSA GIS	127N
1284	AD	127C
1556	AD	128N
1921	AD	128C
0827	CONTROL	129N
1672	CONTROL	129C
2317	CONTROL	130N
	EMORY GIS	130C
	EMORY GIS	131

Precuneus, not DLPFC

2 x 2 DLPFC technical replicates
