# Supplementary material for: Deep proteomic network analysis of Alzheimer’s disease brain reveals alterations in RNA binding proteins and RNA splicing associated with disease
Source: Mol Neurodegener. 2018 Oct 4;13:52. doi: 10.1186/s13024-018-0282-4 (PMC6172707; doi:10.1186/s13024-018-0282-4)
Supplement: Supplementary file 4 — Table S4. List of Biological Terms for GO Network in Figure S8. GO, gene ontology; UP, UniProt; KEGG, Kyoto Encyclopedia of Genes and Genomes; SMART, Simple Modular Architecture Research Tool; FDR, false discovery rate. (DOCX 35 kb) [file 13024_2018_282_MOESM4_ESM.docx]

| **Node** | **Description** | **Source** | **FDR Q Value** |
| --- | --- | --- | --- |
| 1 | EXTRACELLULAR EXOSOME | GO TERM (GO:0070062) | 5.66E-15 |
| 2 | FOCAL ADHESION | GO TERM (GO:0005925) | 4.60E-10 |
| 3 | MEMBRANE | GO TERM (GO:0016020) | 7.81E-09 |
| 4 | CYTOPLASMIC VESICLE | UP KEYWORD | 2.01E-08 |
| 5 | CYTOSKELETON | UP KEYWORD | 3.09E-07 |
| 6 | LIPOPROTEIN | UP KEYWORD | 1.57E-06 |
| 7 | PRENYLATION | UP KEYWORD | 2.89E-06 |
| 8 | GTP BINDING | GO TERM (GO:0005525) | 1.27E-05 |
| 9 | SUBSTANTIA NIGRA DEVELOPMENT | GO TERM (GO:0021762) | 3.00E-05 |
| 10 | MYELIN SHEATH | GO TERM (GO:0043209) | 3.69E-05 |
| 11 | ACTIN BINDING | GO TERM (GO:0003779) | 3.97E-05 |
| 12 | DISEASE MUTATION | UP KEYWORD | 1.75E-04 |
| 13 | CADHERIN BINDING INVOLVED IN CELL-CELL ADHESION | GO TERM (GO:0098641) | 2.46E-04 |
| 14 | CELL-CELL ADHERENS JUNCTION | GO TERM (GO:0005913) | 2.76E-04 |
| 15 | HEREDITARY HEMOLYTIC ANEMIA | UP KEYWORD | 2.89E-04 |
| 16 | POSTSYNAPTIC DENSITY | GO TERM (GO:0014069) | 3.08E-04 |
| 17 | GTPASE ACTIVITY | GO TERM (GO:0003924) | 6.29E-04 |
| 18 | GDP BINDING | GO TERM (GO:0019003) | 6.30E-04 |
| 19 | TRANSPORT | UP KEYWORD | 7.46E-04 |
| 20 | LAMELLIPODIUM | GO TERM (GO:0030027) | 1.29E-03 |
| 21 | ADP-RIBOSYLATION | UP KEYWORD | 1.32E-03 |
| 22 | CARBON METABOLISM (HSA01200) | KEGG PATHWAY | 1.59E-03 |
| 23 | UBIQUITIN PROTEIN LIGASE BINDING | GO TERM (GO:0031625) | 1.83E-03 |
| 24 | REMOVED IN MATURE FORM (PROPEPTIDE) | UP SEQUENCE FEATURE | 1.90E-03 |
| 25 | ACTIN FILAMENT BINDING | GO TERM (GO:0051015) | 2.15E-03 |
| 26 | AXON GUIDANCE (HSA04360) | KEGG PATHWAY | 3.45E-03 |
| 27 | NEURON PROJECTION | GO TERM (GO:0043005) | 3.59E-03 |
| 28 | NAD BINDING | GO TERM (GO:0051287) | 3.64E-03 |
| 29 | SRP-DEPENDENT COTRANSLATIONAL PROTEIN TARGETING TO MEMBRANE | GO TERM (GO:0006614) | 3.96E-03 |
| 30 | CELL-CELL ADHESION | GO TERM (GO:0098609) | 4.22E-03 |
| 31 | PROTEOGLYCANS IN CANCER (HSA05205) | KEGG PATHWAY | 4.43E-03 |
| 32 | ACTIN CYTOSKELETON ORGANIZATION | GO TERM (GO:0030036) | 4.51E-03 |
| 33 | MAGNESIUM ION BINDING | GO TERM (GO:0000287) | 5.08E-03 |
| 34 | ATP BINDING | GO TERM (GO:0005524) | 5.09E-03 |
| 35 | TRANSLATIONAL INITIATION | GO TERM (GO:0006413) | 5.17E-03 |
| 36 | RIBOSOME | GO TERM (GO:0005840) | 6.79E-03 |
| 37 | MITOCHONDRION | GO TERM (GO:0005739) | 1.04E-02 |
| 38 | KINASE | UP KEYWORD | 1.09E-02 |
| 39 | RIBONUCLEOPROTEIN | UP KEYWORD | 1.27E-02 |
| 40 | AXON | GO TERM (GO:0030424) | 1.47E-02 |
| 41 | RAP1 SIGNALING PATHWAY (HSA04015) | KEGG PATHWAY | 1.49E-02 |
| 42 | ENDOCYTOSIS (HSA04144) | KEGG PATHWAY | 1.54E-02 |
| 43 | NEUROTROPHIN SIGNALING PATHWAY (HSA04722) | KEGG PATHWAY | 1.56E-02 |
| 44 | SMALL GTPASE MEDIATED SIGNAL TRANSDUCTION | GO TERM (GO:0007264) | 1.58E-02 |
| 45 | FOCAL ADHESION (HSA04510) | KEGG PATHWAY | 1.72E-02 |
| 46 | NUCLEAR-TRANSCRIBED MRNA CATABOLIC PROCESS, NONSENSE-MEDIATED DECAY | GO TERM (GO:0000184) | 1.77E-02 |
| 47 | UBL CONJUGATION | UP KEYWORD | 2.17E-02 |
| 48 | MICROTUBULE | UP KEYWORD | 2.22E-02 |
| 49 | NEURODEGENERATION | UP KEYWORD | 2.66E-02 |
| 50 | MENTAL RETARDATION | UP KEYWORD | 2.99E-02 |
| 51 | POLY(A) RNA BINDING | GO TERM (GO:0044822) | 3.28E-02 |
| 52 | REGULATION OF ACTIN CYTOSKELETON (HSA04810) | KEGG PATHWAY | 3.42E-02 |
| 53 | CELL DIVISION | UP KEYWORD | 3.54E-02 |
| 54 | SECRETORY GRANULE | GO TERM (GO:0030141) | 3.86E-02 |
| 55 | DIFFERENTIATION | UP KEYWORD | 4.47E-02 |
| 56 | PYRUVATE METABOLISM (HSA00620) | KEGG PATHWAY | 4.91E-02 |
| 57 | NEUROFIBRILLARY TANGLE | GO TERM (GO:0097418) | 5.01E-02 |
| 58 | SYNAPTIC VESICLE MEMBRANE | GO TERM (GO:0030672) | 5.22E-02 |
| 59 | NEUROGENESIS | UP KEYWORD | 5.58E-02 |
| 60 | NON-ALCOHOLIC FATTY LIVER DISEASE (NAFLD) (HSA04932) | KEGG PATHWAY | 5.83E-02 |
| 61 | CELL-CELL JUNCTION | GO TERM (GO:0005911) | 5.85E-02 |
| 62 | CHEMOKINE SIGNALING PATHWAY (HSA04062) | KEGG PATHWAY | 5.86E-02 |
| 63 | RIBOSOME (HSA03010) | KEGG PATHWAY | 5.89E-02 |
| 64 | MICROTUBULE | GO TERM (GO:0005874) | 5.93E-02 |
| 65 | LONG-TERM POTENTIATION (HSA04720) | KEGG PATHWAY | 6.08E-02 |
| 66 | FOXO SIGNALING PATHWAY (HSA04068) | KEGG PATHWAY | 6.34E-02 |
| 67 | ERBB SIGNALING PATHWAY (HSA04012) | KEGG PATHWAY | 6.69E-02 |
| 68 | ALZHEIMER'S DISEASE (HSA05010) | KEGG PATHWAY | 6.73E-02 |
| 69 | EFH (SM00054) | SMART | 6.78E-02 |
| 70 | GROWTH CONE | GO TERM (GO:0030426) | 6.80E-02 |
| 71 | PARKINSON'S DISEASE (HSA05012) | KEGG PATHWAY | 7.01E-02 |
| 72 | METABOLIC PATHWAYS (HSA01100) | KEGG PATHWAY | 7.42E-02 |
| 73 | MICROTUBULE ORGANIZING CENTER | GO TERM (GO:0005815) | 7.89E-02 |
| 74 | SYNAPTIC VESICLE | GO TERM (GO:0008021) | 7.98E-02 |
| 75 | CELL JUNCTION | GO TERM (GO:0030054) | 8.86E-02 |
| 76 | ENDOCYTOSIS | UP KEYWORD | 9.11E-02 |
| 77 | GTPASE ACTIVATION | UP KEYWORD | 9.46E-02 |
| 78 | BACTERIAL INVASION OF EPITHELIAL CELLS (HSA05100) | KEGG PATHWAY | 9.50E-02 |

**Table S4**
