# Supplementary material for: Deep proteomic network analysis of Alzheimer’s disease brain reveals alterations in RNA binding proteins and RNA splicing associated with disease
Source: Mol Neurodegener. 2018 Oct 4;13:52. doi: 10.1186/s13024-018-0282-4 (PMC6172707; doi:10.1186/s13024-018-0282-4)
Supplement: Supplementary file 5 — Table S5. Number of Alternative Exon-Exon Junction Peptides Identified by TMT-LysC and LFQ-trypsin Approaches. The number of alt-EEjxn peptides identified by matching to the listed databases (Swiss-Prot, Trembl, or RNAseq) is shown, along with the number of quantifiable alt-EEjxn peptides. A peptide was considered quantifiable in this analysis if it had a minimum of 2 measurements in at least 2 different case groups. RNAseq data from control and AD patient brains (n = 6) were used to generate the RNAseq alt-EEjxn peptide database, as described in Methods. (DOCX 28 kb) [file 13024_2018_282_MOESM5_ESM.docx]

|  | **Alternative Exon-Exon Junction Peptide Database** | | |  |
| --- | --- | --- | --- | --- |
|  | Swiss-Prot + Trembl + RNAseq | Trembl + RNAseq | RNAseq Only | # Quantifiable |
| LFQ-trypsin | 5746 | 207 | 163 | 3949 |
| TMT-LysC | 4830 | 52 | 161 | 4672 |

**Table S5**
