# Supplementary material for: Deep proteomic network analysis of Alzheimer’s disease brain reveals alterations in RNA binding proteins and RNA splicing associated with disease
Source: Mol Neurodegener. 2018 Oct 4;13:52. doi: 10.1186/s13024-018-0282-4 (PMC6172707; doi:10.1186/s13024-018-0282-4)
Supplement: Supplementary file 6 — Table S6. Number of Alternative Exon-Exon Junctions in AD Risk Factor Proteins. From the twenty proteins identified as risk factors for AD by GWAS at genome-wide significance [12], five had alt-EEjxn peptides that were observed and quantifiable in the BLSA-TMT analysis (observed). The number of observed and quantifiable alt-EEjxn peptides for each of these five proteins was a subset of the total number of alt-EEjxn peptides predicted to exist after LysC digestion (peptide database). This number was a further subset of the total number of alt-EEjxns observed for each of the five proteins from RNAseq data (transcript level). For details on generation of the peptide database and transcript level numbers, see Methods. (DOCX 28 kb) [file 13024_2018_282_MOESM6_ESM.docx]

|  | Number of Alternative Exon-Exon Junctions | | |
| --- | --- | --- | --- |
| Gene | Observed | Peptide Database | Transcript Level |
| BIN1 | 9 | 16 | 23 |
| PICALM | 4 | 11 | 74 |
| PTK2B | 6 | 13 | 73 |
| CELF1 | 1 | 2 | 49 |
| FERMT2 | 3 | 9 | 29 |

**Table S6**
