# Supplementary material for: Deep proteomic network analysis of Alzheimer’s disease brain reveals alterations in RNA binding proteins and RNA splicing associated with disease
Source: Mol Neurodegener. 2018 Oct 4;13:52. doi: 10.1186/s13024-018-0282-4 (PMC6172707; doi:10.1186/s13024-018-0282-4)
Supplement: Supplementary file 7 — Table S7. AD Risk Factor Protein Alternative Exon-Exon Junction Peptides Significantly Altered by Case Status. Of the five AD risk factor proteins that had quantifiable alt-EEjxn peptides in the BLSA-TMT analysis, three had alt-EEjxn peptides that were significantly, or nearly significantly, different in abundance by case status. For PTK2B 20038, module eigenprotein correlation was not performed due to the number of missing values for quantitation (≥25). A complete description of each alt-EEjxn peptide is provided in Supplementary Data. ME bicor, module eigenprotein bicorrelation; kME, correlation value to the module eigenprotein; AD, Alzheimer’s disease; AsymAD, asymptomatic Alzheimer’s disease. (DOCX 29 kb) [file 13024_2018_282_MOESM7_ESM.docx]

| Protein | EEJxn  Pep ID | Exon-Exon Junction Peptide Sequence | Difference, Log2(avg)-Log2(avg),  *P* Value | Highest ME Bicor, kME |
| --- | --- | --- | --- | --- |
| BIN1 | 51576 | VQAQHDYTATDTDELQLK | AsymAD-AD, 0.25, 0.045 | M2 (blue), 0.71 |
| BIN1 | 51573 | AGDVVLVIPFQNPEEQDEGWLMGVK | AsymAD-AD, 0.34, 0.035 | M2 (blue), 0.70 |
| PTK2B | 20040 | LIGSVDDLLPSLPSSSRTEIEGTQK | AsymAD-AD, 0.14, 0.063 | M30 (steelblue), 0.56 |
| PTK2B | 20038 | NVGLTLRK | AsymAD-AD, 0.53, 0.028  Control-AD, 0.49, 0.021 | NA |
| FERMT2 | 76255 | LLIPVAEGMNEIWLRCDNEK | Control-AsymAD, -0.42, 0.008 | M23 (darkturqoise), 0.35 |

**Table S7**
