# Supplementary figures and images for: Deep proteomic network analysis of Alzheimer’s disease brain reveals alterations in RNA binding proteins and RNA splicing associated with disease
Source: Mol Neurodegener. 2018 Oct 4;13:52. doi: 10.1186/s13024-018-0282-4 (PMC6172707; doi:10.1186/s13024-018-0282-4)

CT  
1036 1312 1313 1471 1517 1672 2020 2021 2027 2066 2151 2228

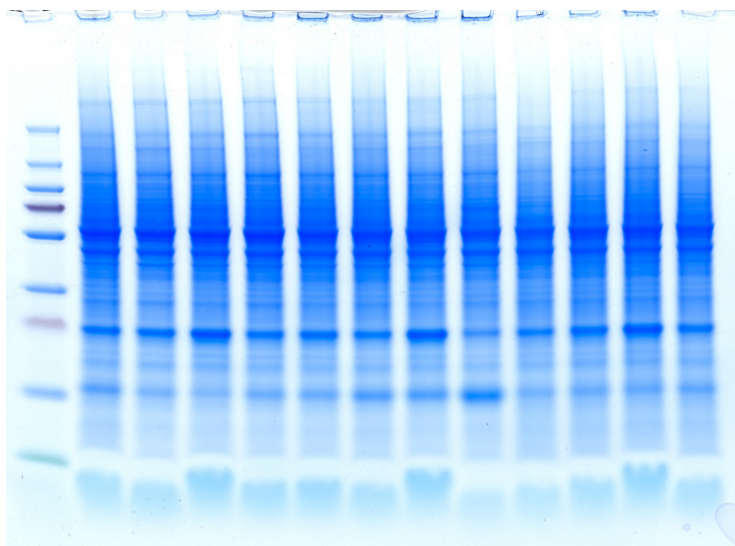

CT AD  
2317 1284 1407 1430 1556 1649 1712 1735 1839 1875 1921 1973

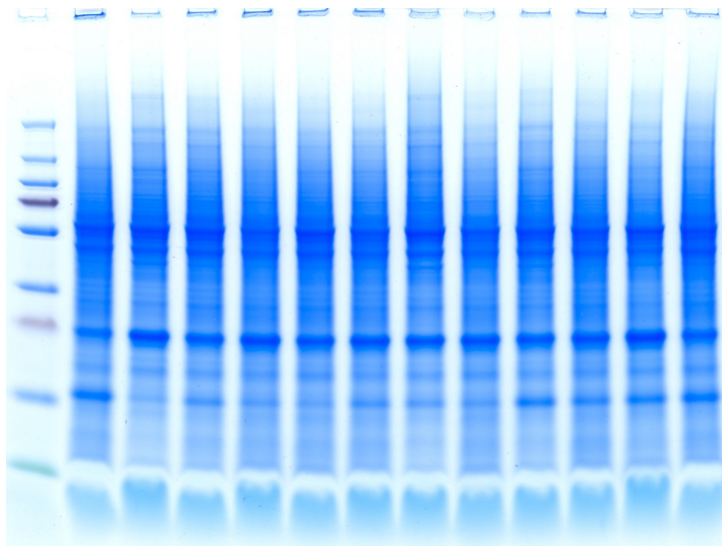

AD AD AD AD AD AD AD AD AD AS AS AS  
1984 2004 2023 2028 2032 2157 2184 2226 2274 0803 1479 1591

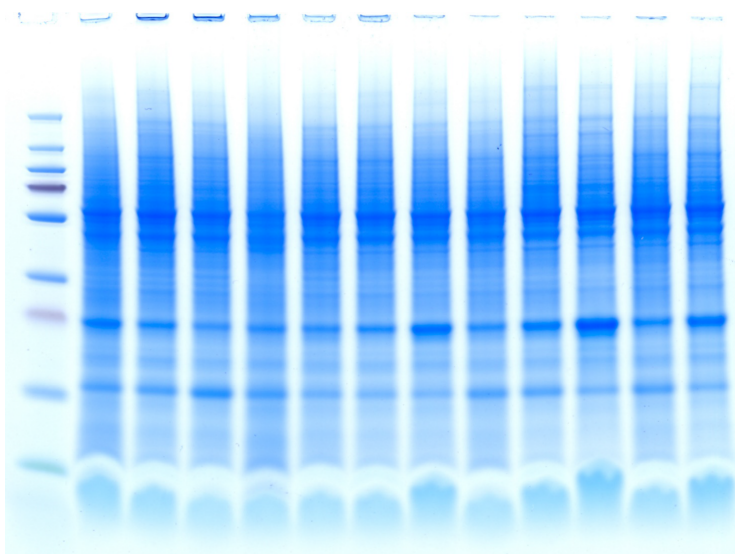

AS  
1720 1734 1843 1867 1924 2011 2037 2069 2190 2316 2342

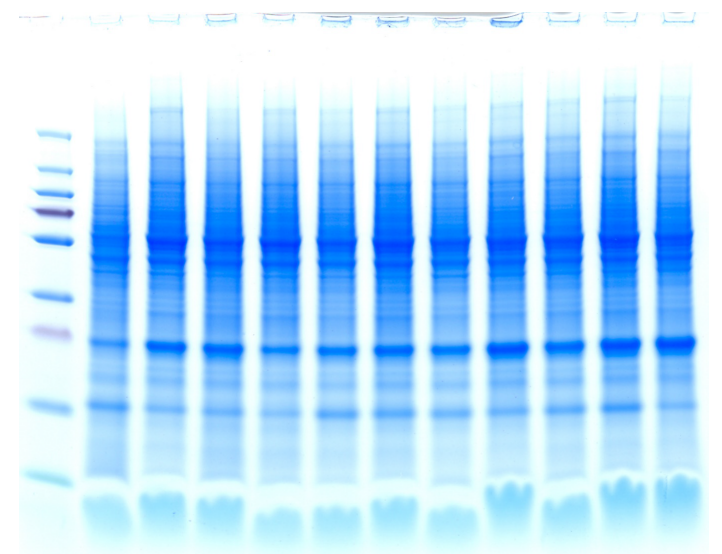

MCI  
1273 1341 1372 1543 1548 1603 1669 1790 1805 1969 2000

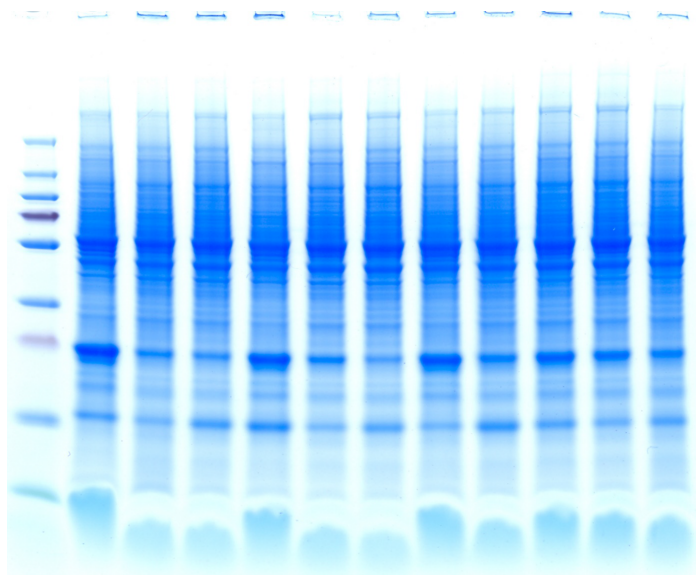

Supplement: Supplementary file 8 — Figure S1. SDS-PAGE of Brain Homogenates. Dorsolateral prefrontal cortex (DLPFC) brain tissue homogenates from cases shown in Table S1 were analyzed by SDS-PAGE to assess sample integrity prior to TMT labeling and mass spectrometry analysis. Gels were stained with Coomassie Blue to visualize protein. AD, Alzheimer’s disease; AS, asymptomatic Alzheimer’s disease; CT, control; MCI, mild cognitive impairment. (PDF 22000 kb) [file 13024_2018_282_MOESM8_ESM.pdf]

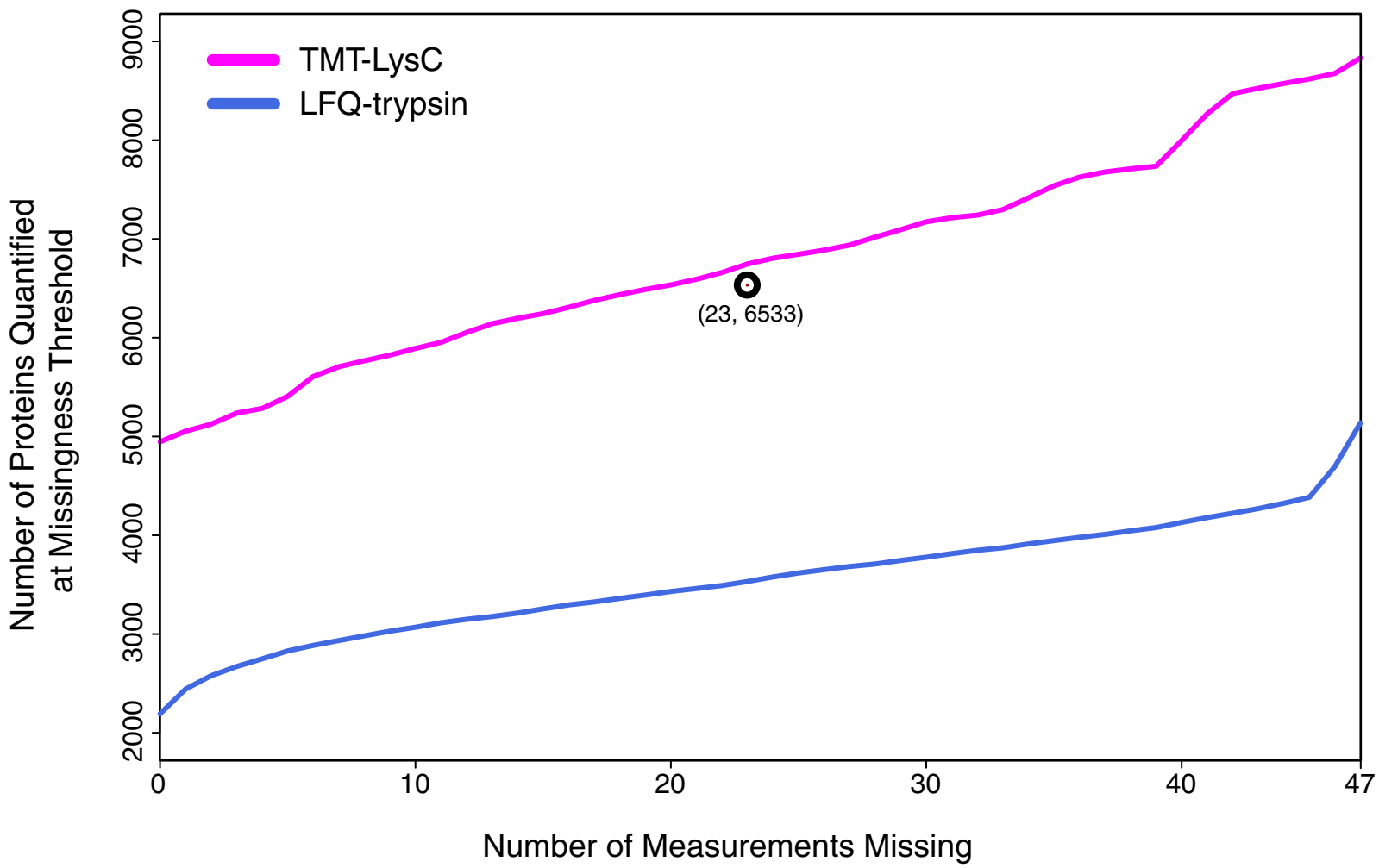

Supplement: Supplementary file 9 — Figure S2. Protein Quantitation in TMT-LysC and LFQ-trypsin Analyses. The relationship between the number of quantifiable proteins at a given threshold of missing values in the 47 brain samples from the BLSA cohort for TMT-LysC and LFQ-trypsin analyses is shown. The point at 23 samples and 6533 proteins represents the threshold used for the TMT-LysC analysis pipeline in this study. This point falls slightly below the TMT curve because 11 MCI samples were included in the TMT analysis workflow, for a total of 58 samples, but were later dropped from the analysis (see Methods). The increased number of samples when including the 11 MCI cases slightly reduced the number of quantifiable proteins at the ~ 50% missing value threshold. (PDF 79 kb) [file 13024_2018_282_MOESM9_ESM.pdf]

**A**Amyloid- $\beta$ 

D<sup>1</sup>AEFRHDSGYEVHHQK **LVFFAEDVGSNK**GAIIGLMVGGVVIA<sup>42</sup>  
(residues 17-28)

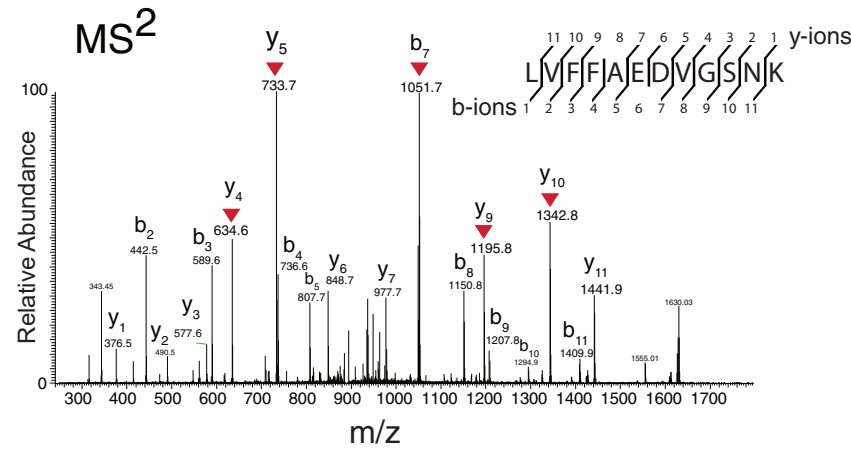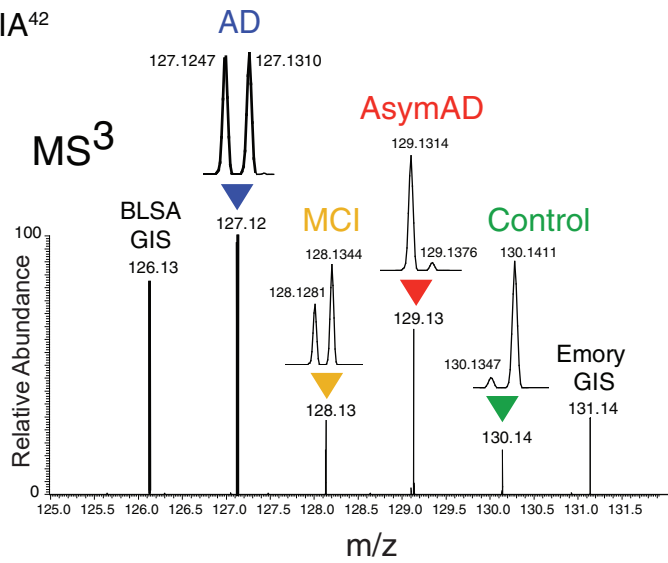**B**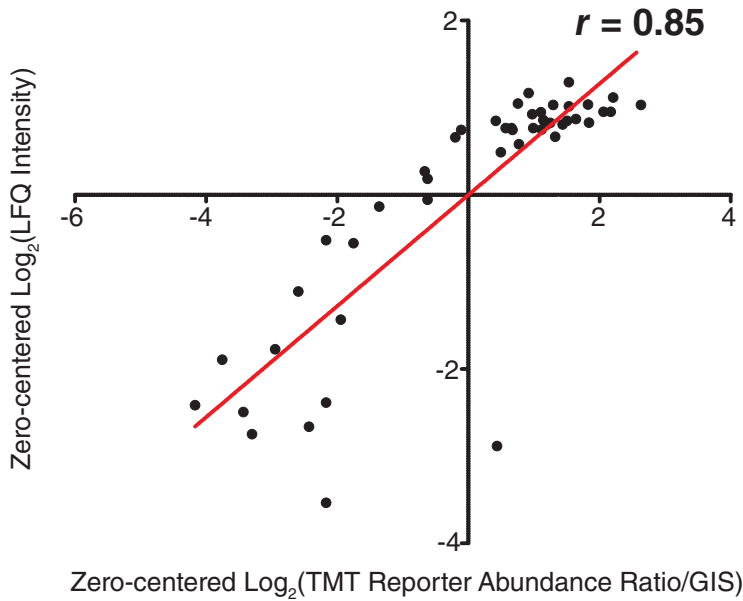**C**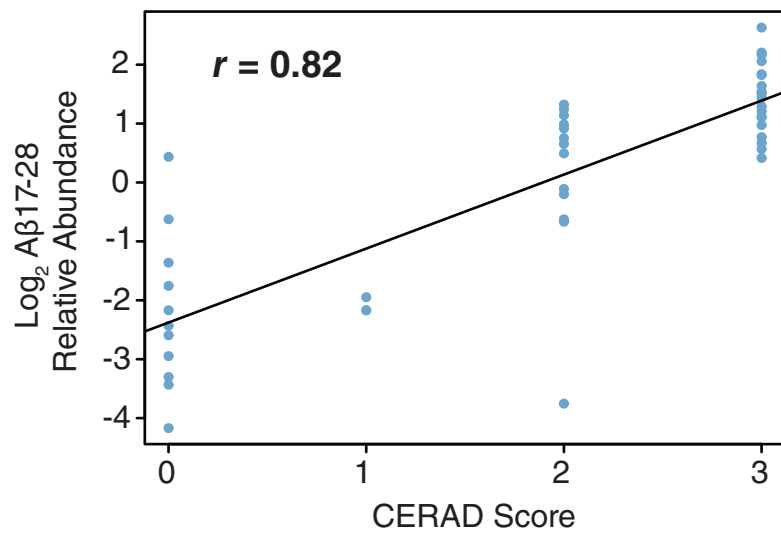**D**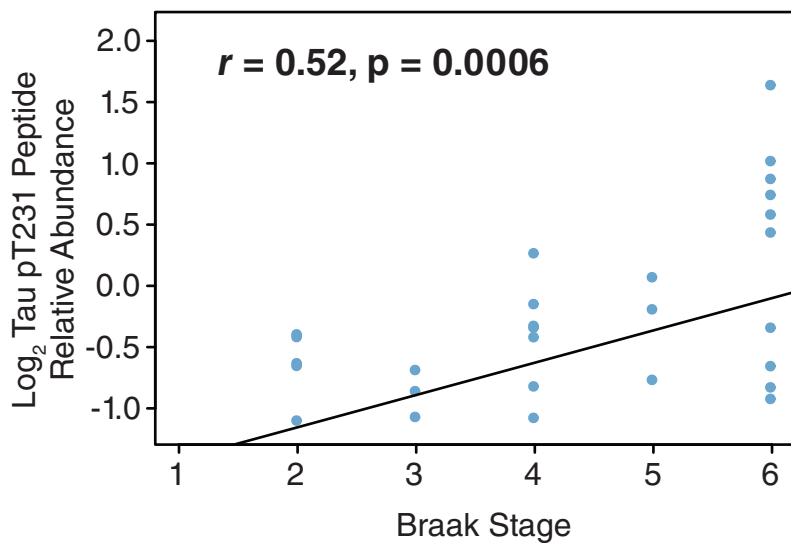

Supplement: Supplementary file 10 — Figure S3. Measurement of Aβ and Tau. (A-D) Total amyloid-β levels were measured using the Aβ17–28 peptide fragment (A, left). Aβ levels were quantified in the TMT-LysC analysis across case groups using SPS-MS3 reporter ions to Aβ17–28 (A, right). (B) The Aβ17–28 peptide was quantified by LFQ extracted ion current intensity [5], and compared to quantification using TMTs with SPS-MS3 reporter ion relative intensities normalized to the global internal standard (GIS). (C) The Aβ17–28 peptide was quantified by TMT across the 47 cases and correlated to CERAD score, a histopathological measure of neuritic amyloid plaque burden. (D) The tau pT231 peptide (VAVVRpTPPKSPSSAK), which is derived from the proline-rich domain, was quantified by TMT and correlated with Braak stage, a histological staging system for tau neurofibrillary tangle burden. (PDF 299 kb) [file 13024_2018_282_MOESM10_ESM.pdf]

# M7 Black Module

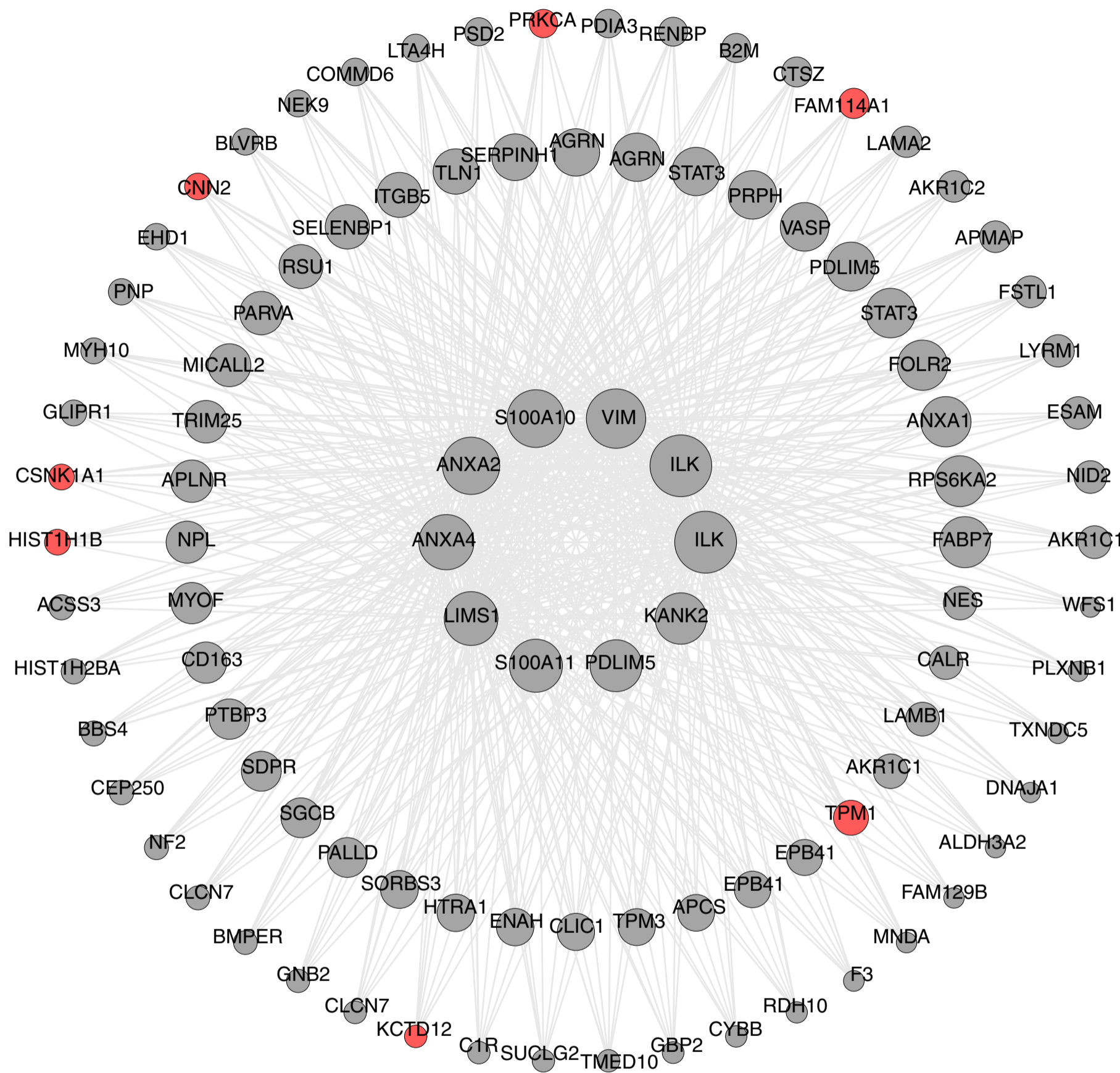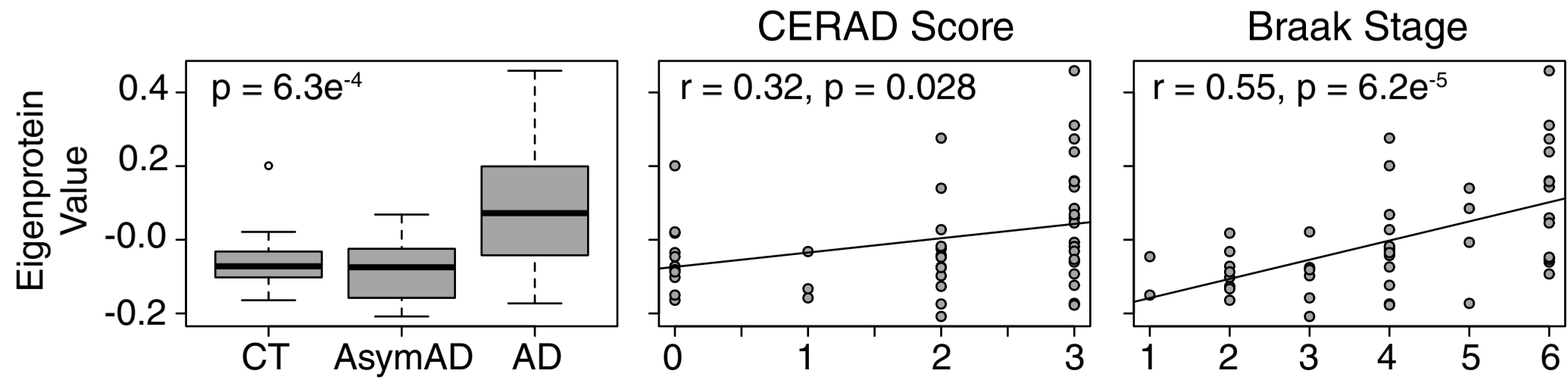

# M27 White Module

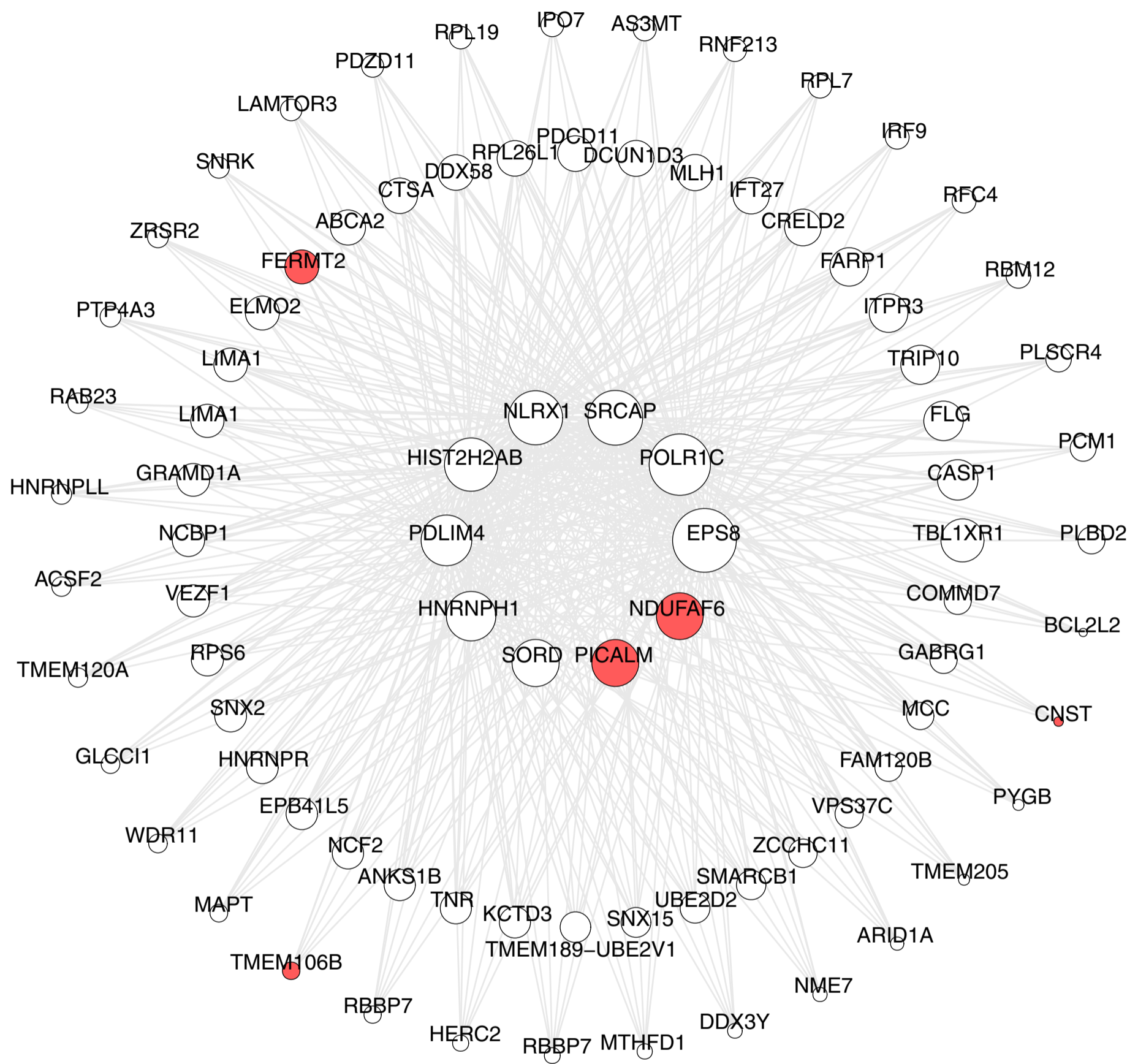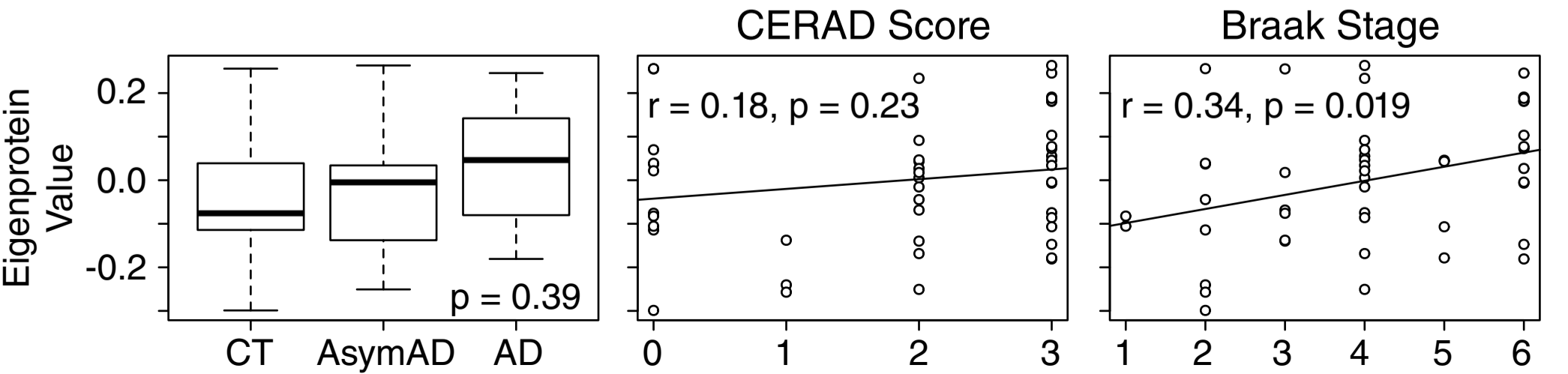

# M33 Darkolivegreen Module

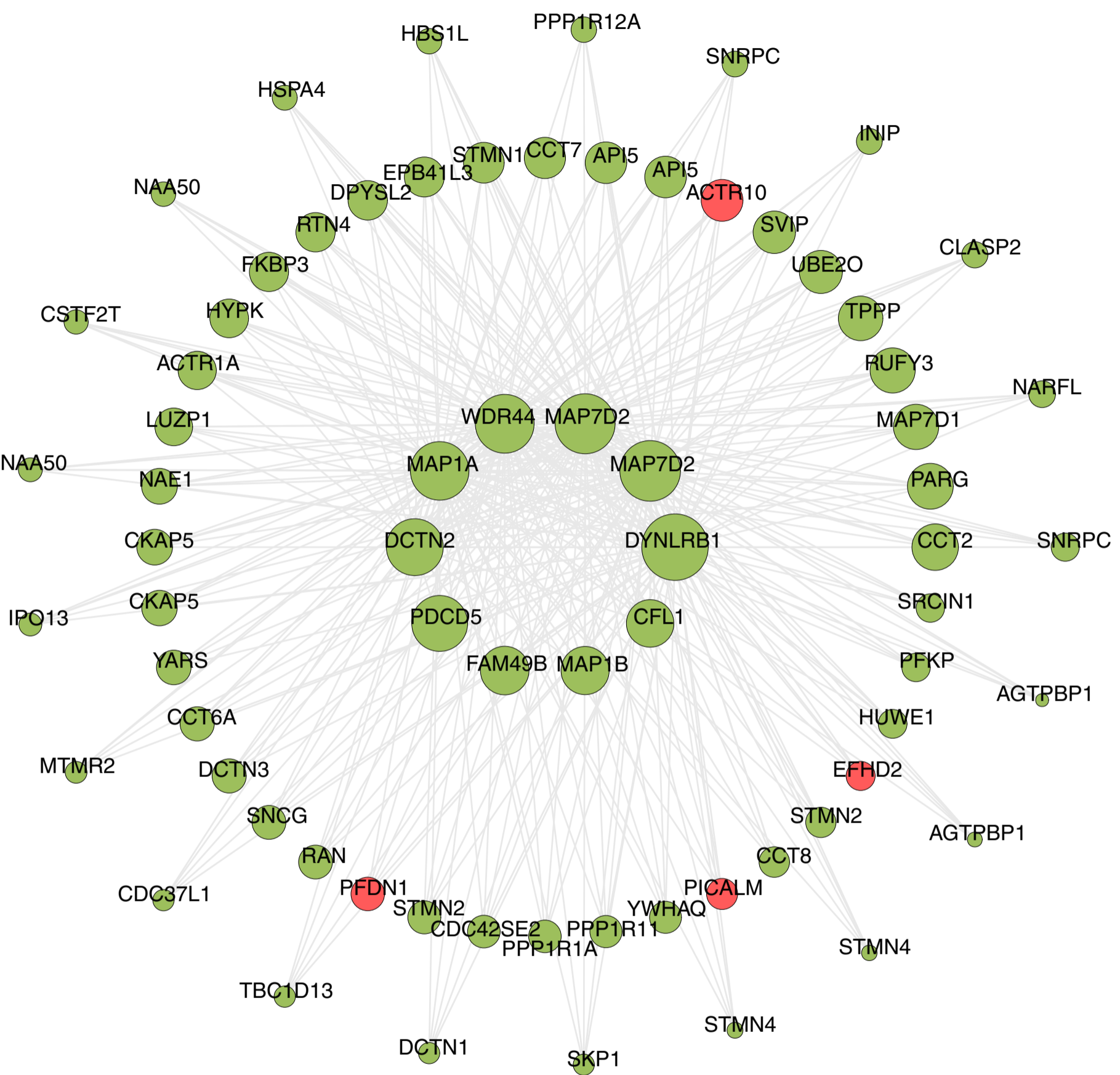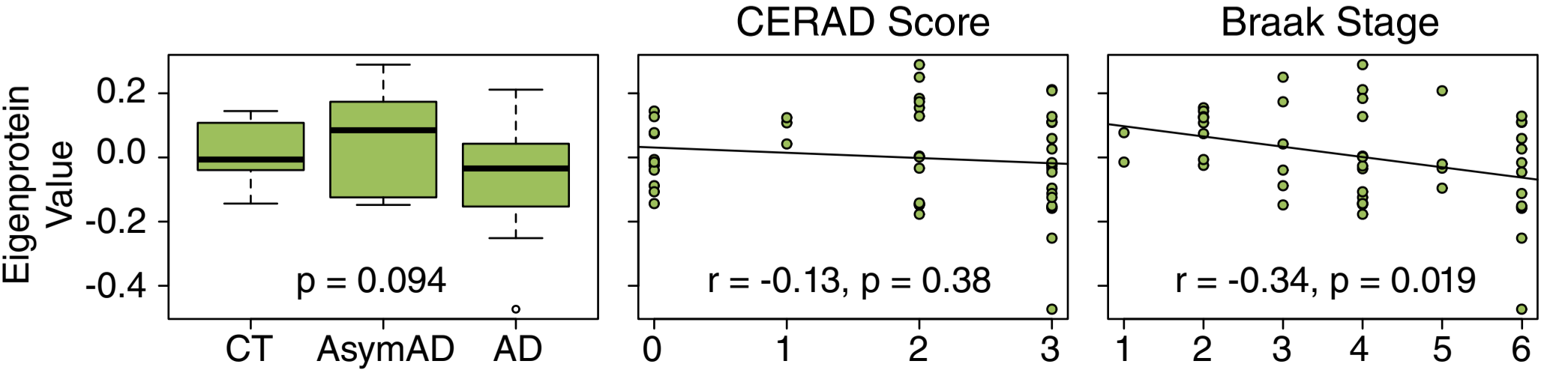

Supplement: Supplementary file 12 — Figure S5. TMT Protein Network Modules Enriched for AD Risk Factors. Graphical representation of the correlation relationships among TMT network module proteins for the four modules identified to contain enrichment of AD risk factors from GWAS, along with the relationship of each module to case status, neuritic amyloid plaque load (CERAD score), and tau tangle burden (Braak stage). Proteins identified by GWAS as AD risk factors are highlighted in red. Only the top 100 proteins by kME value are shown for the M4 yellow (257 total proteins) and M7 black (162 total proteins) modules. (PDF 462 kb) [file 13024_2018_282_MOESM12_ESM.pdf]

Cell Type

CERAD Amyloid Score

Braak Stage

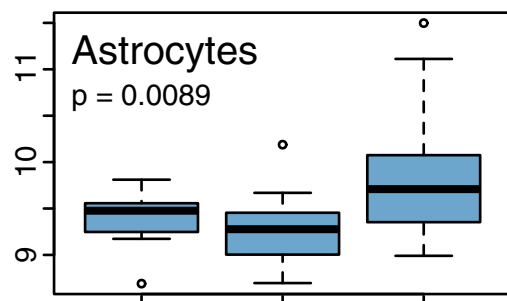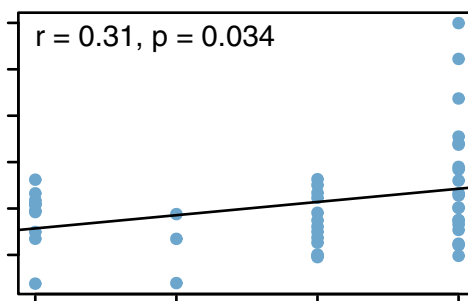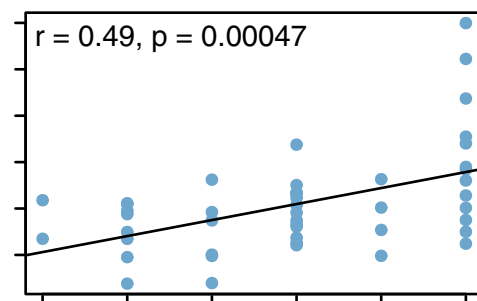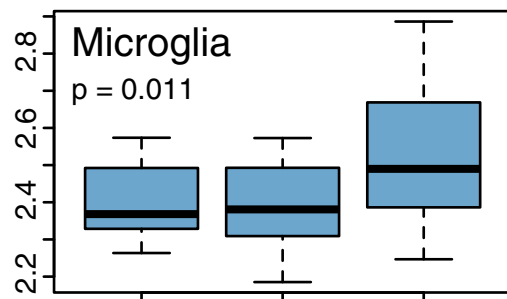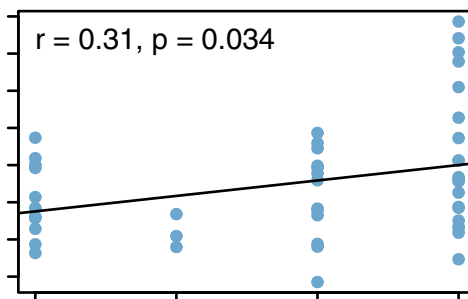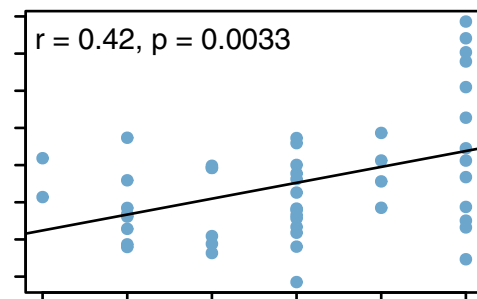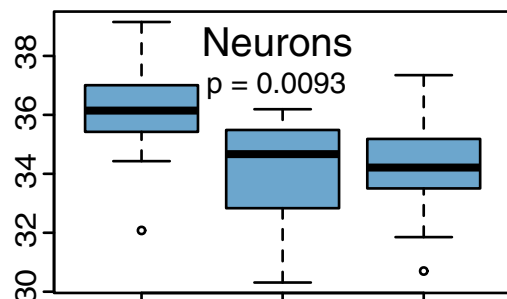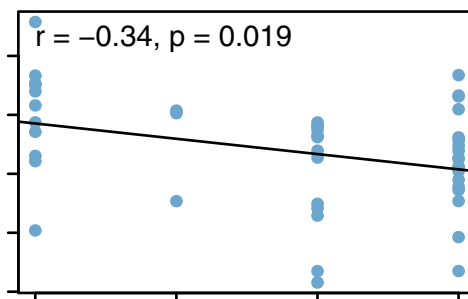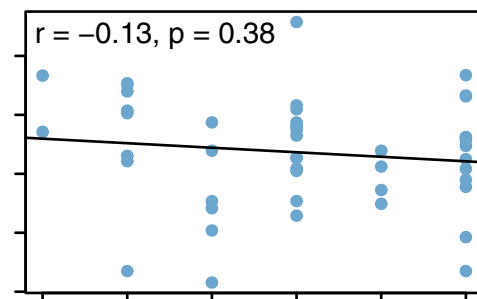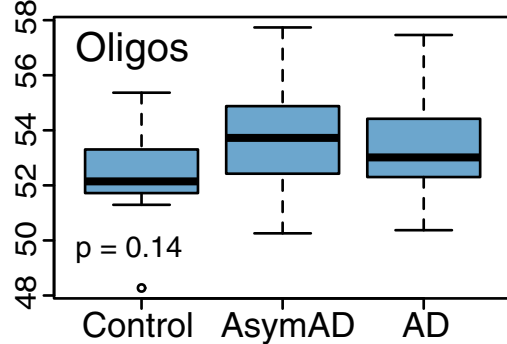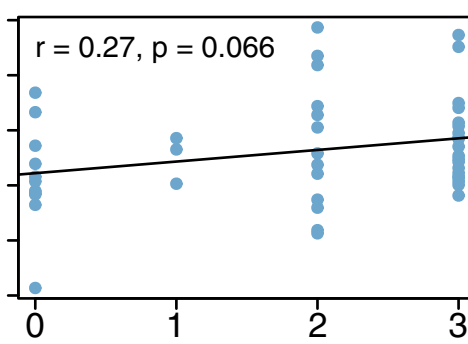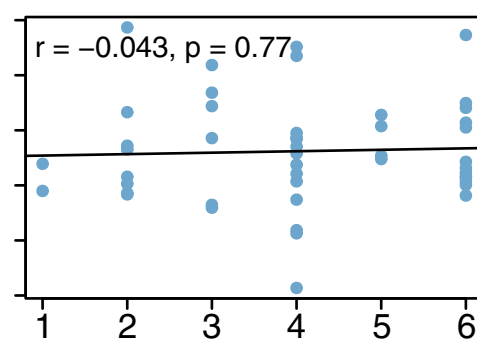

Supplement: Supplementary file 13 — Figure S6. Cell Type Population Changes Associated with AD and Correlation with Amyloid and Tau Pathology. The abundance of cell type-specific protein markers of astrocytes, microglia, neurons, and oligodendrocytes (oligos) was used to calculate the percentage of each cell type in control, asymptomatic AD (AsymAD), and AD brain tissue (see Methods). Percentage cell type was then correlated with the degree of neuritic amyloid plaque pathology (CERAD Amyloid Score) and tau tangle burden (Braak Stage) across all brains. (PDF 141 kb) [file 13024_2018_282_MOESM13_ESM.pdf]

**Down AD: 190**

**Up AD: 160**

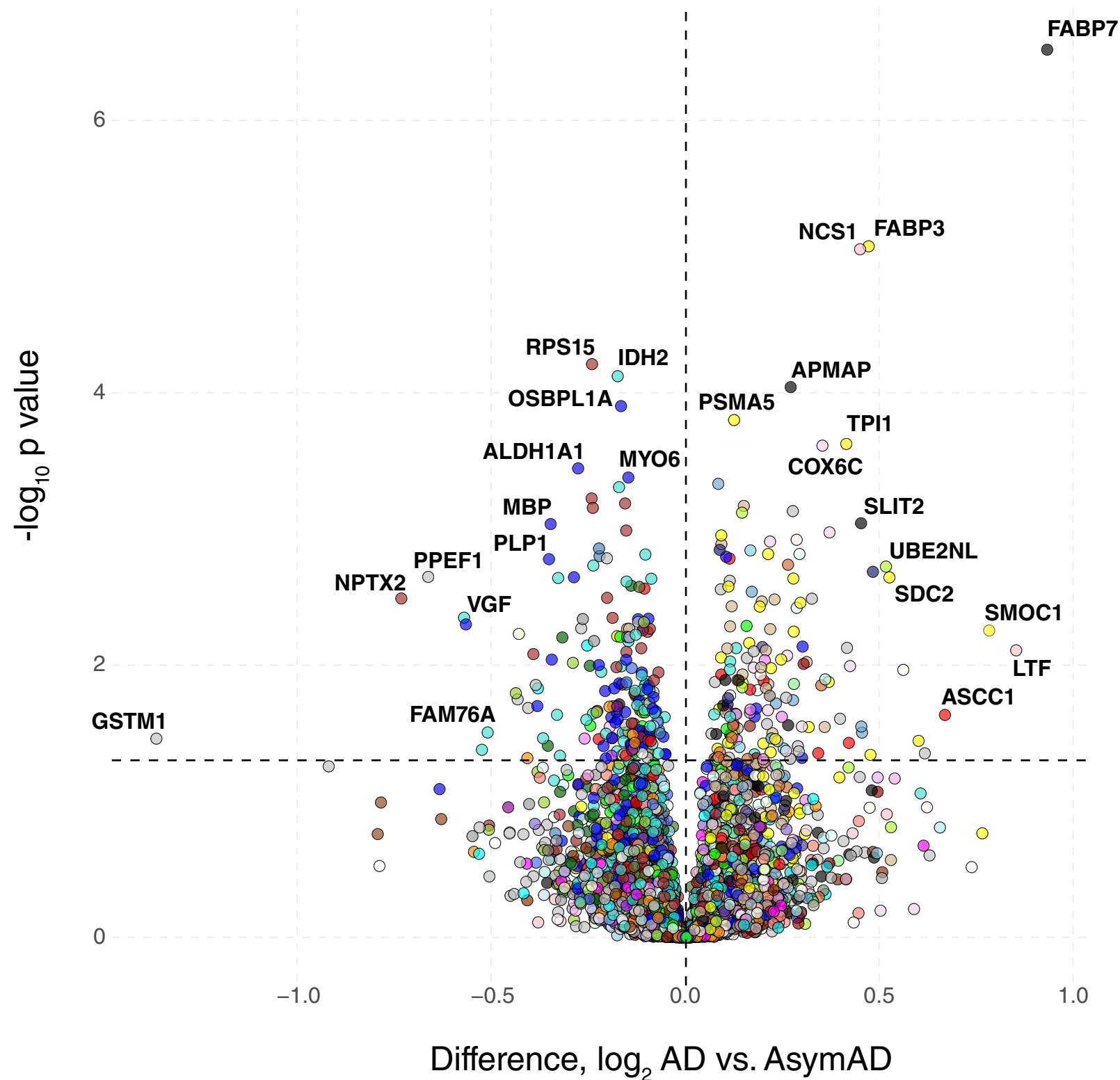

Supplement: Supplementary file 14 — Figure S7. Differential Protein Abundance between AD and AsymAD. Proteins that were significantly increased (160) or decreased (190) in AD compared to AsymAD, color-coded by TMT network module membership, are shown. The horizontal dotted line represents p = 0.05. Interactive plots for AD vs. AsymAD, AD vs. control, and AsymAD vs. control differential protein abundance are provided in Supplementary Data. (PDF 10400 kb) [file 13024_2018_282_MOESM14_ESM.pdf]

A

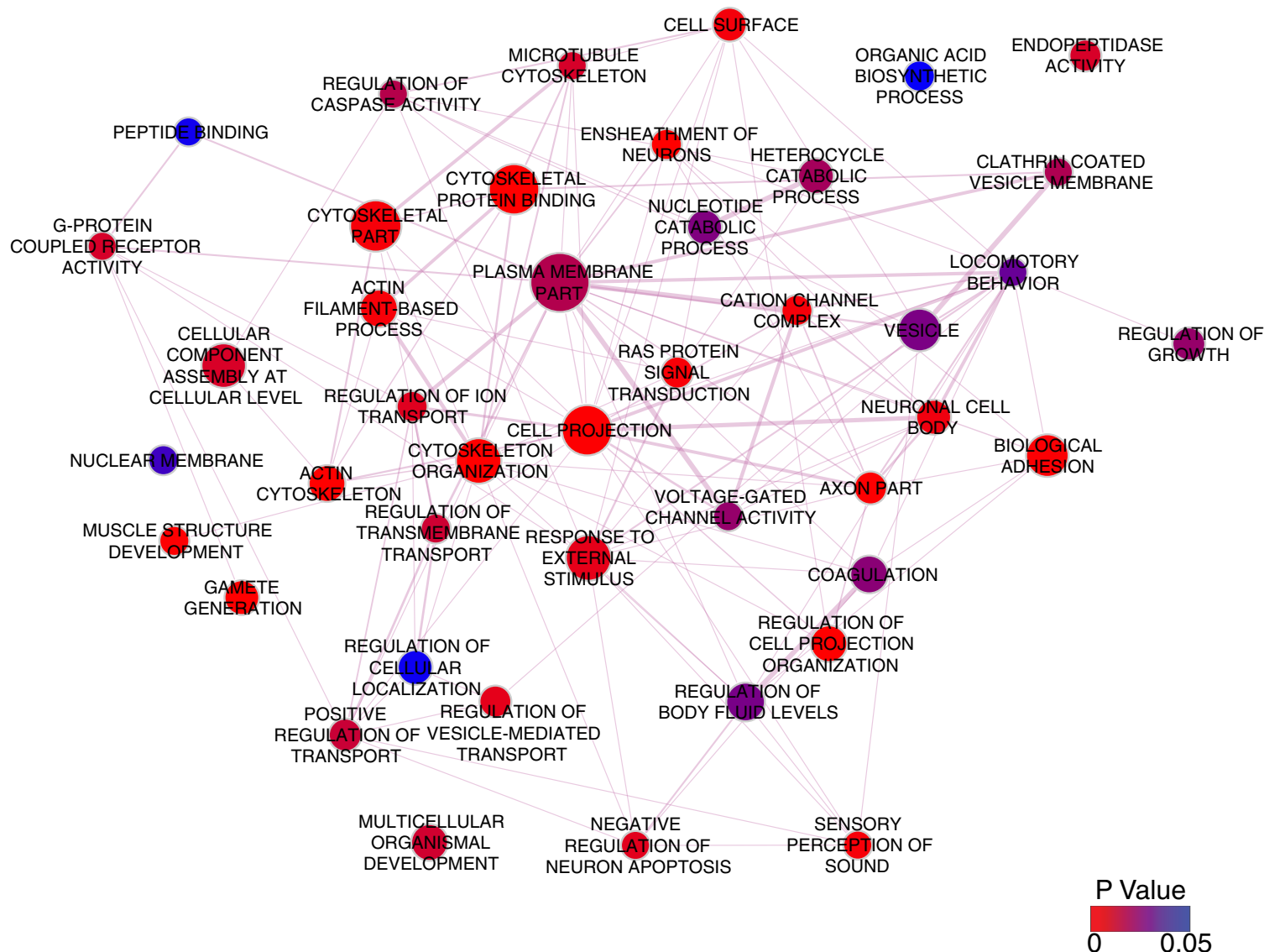

B

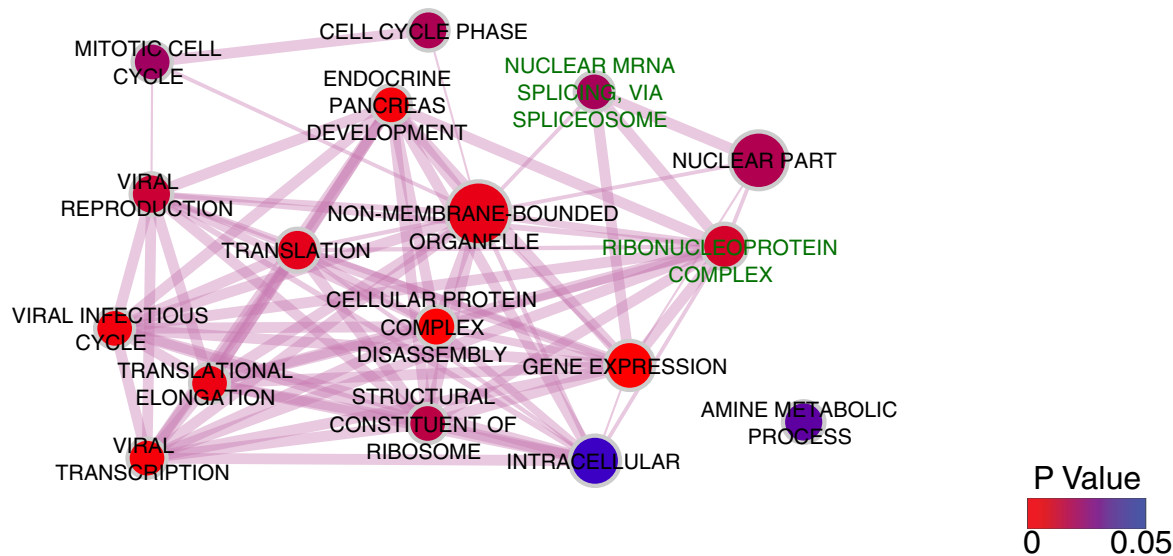

Supplement: Supplementary file 15 — Figure S8. GO Network Analysis for Differential Protein Abundance Between AsymAD and Control. (A, B) Proteins with significant differences in abundance between asymptomatic AD and control before (A) after (B) cell type deconvolution were analyzed by gene ontology (GO) network analysis. Only two nodes were significant before cell type deconvolution, and no nodes were significant after cell type deconvolution, by false discovery rate (FDR) Q value statistic. Therefore, significance values are represented by the less stringent uncorrected p value. RNA binding protein nodes are highlighted in green. (PDF 228 kb) [file 13024_2018_282_MOESM15_ESM.pdf]

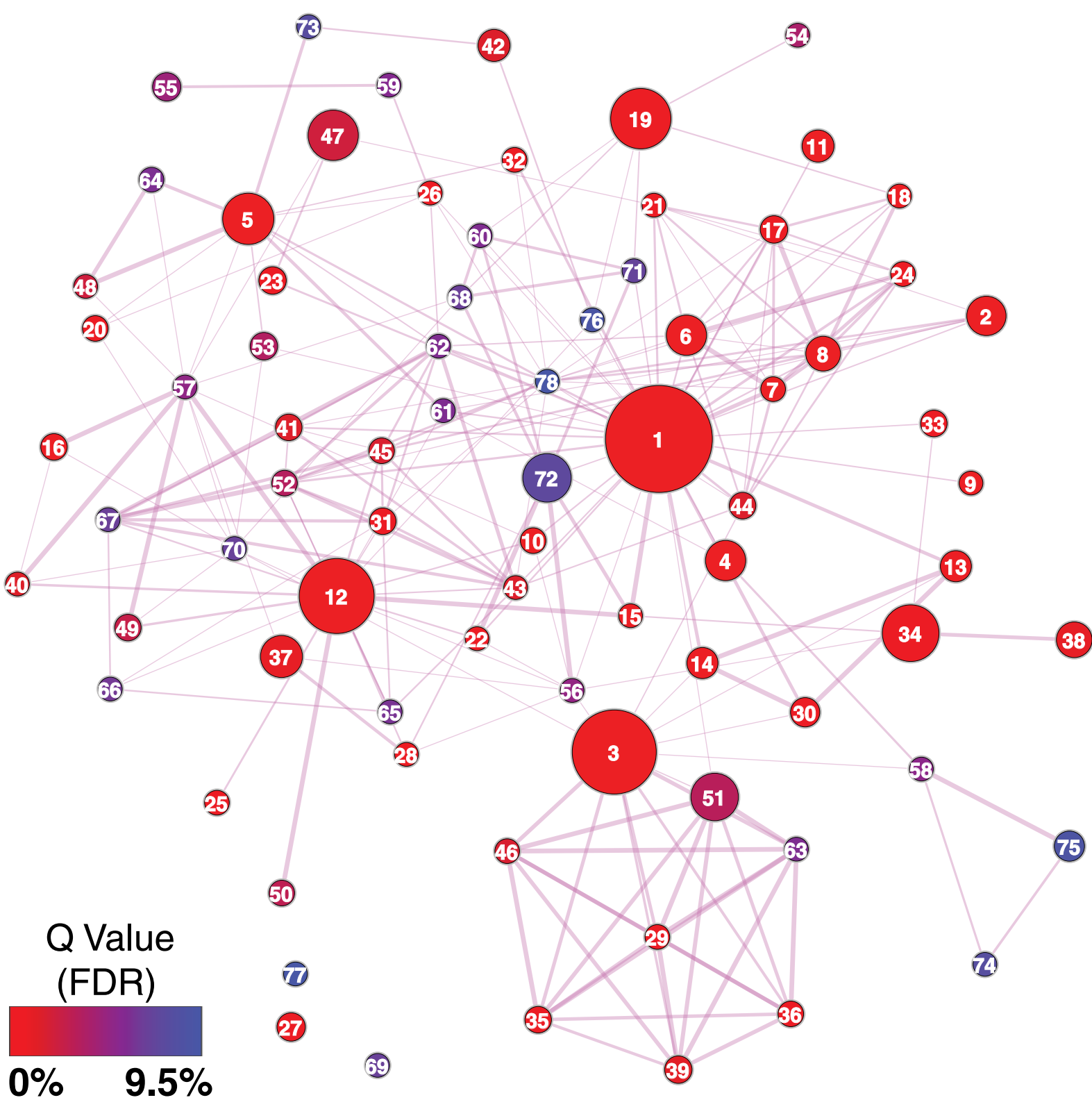

Supplement: Supplementary file 16 — Figure S9. GO Network Analysis for Differential Protein Abundance Between AD and AsymAD. Proteins with significant differences in abundance between asymptomatic AD and AD after cell type deconvolution were analyzed by gene ontology (GO) network analysis. A complete list of biological terms that correspond to each node in the network, along with the source for the term and the false discovery rate (FDR) Q value statistic, is given in Table S4. (PDF 244 kb) [file 13024_2018_282_MOESM16_ESM.pdf]

**A**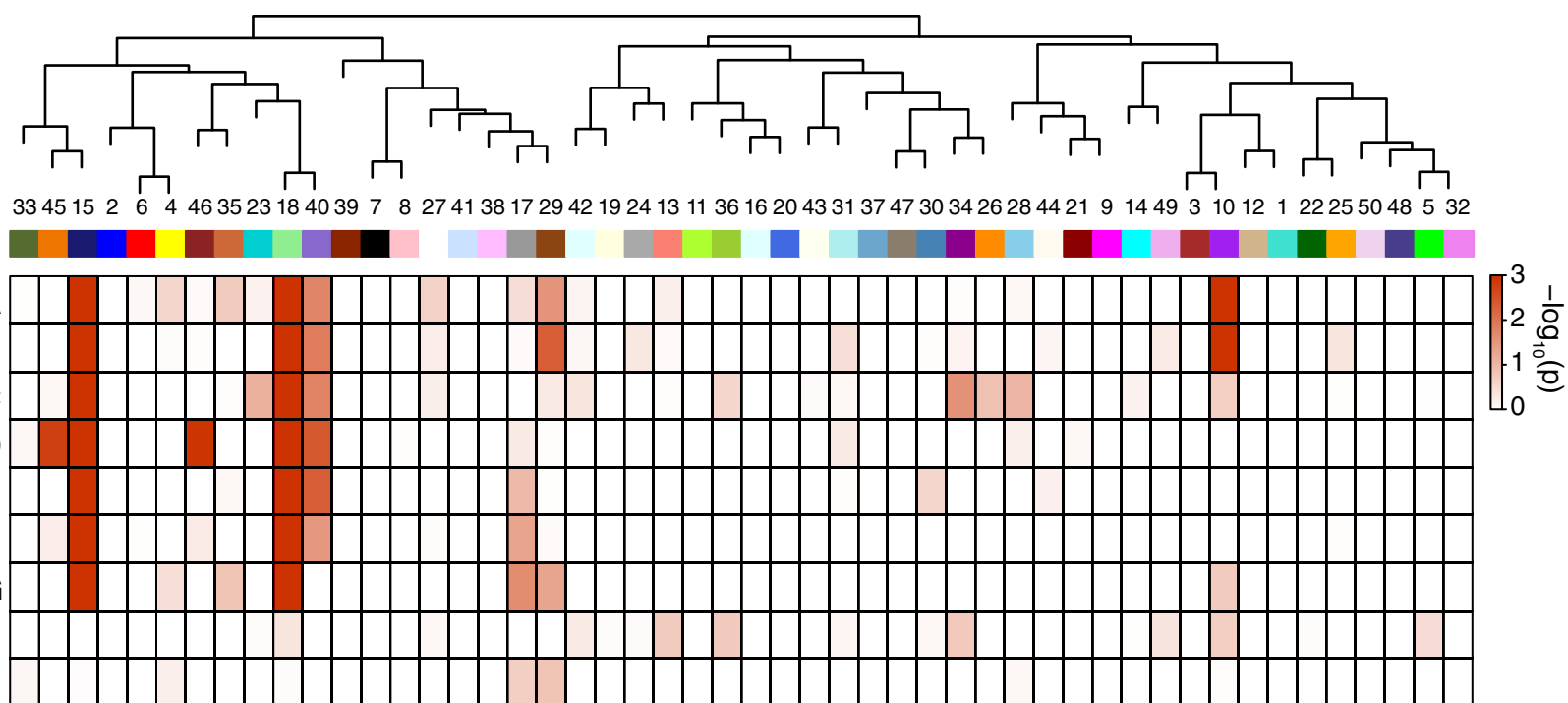**B**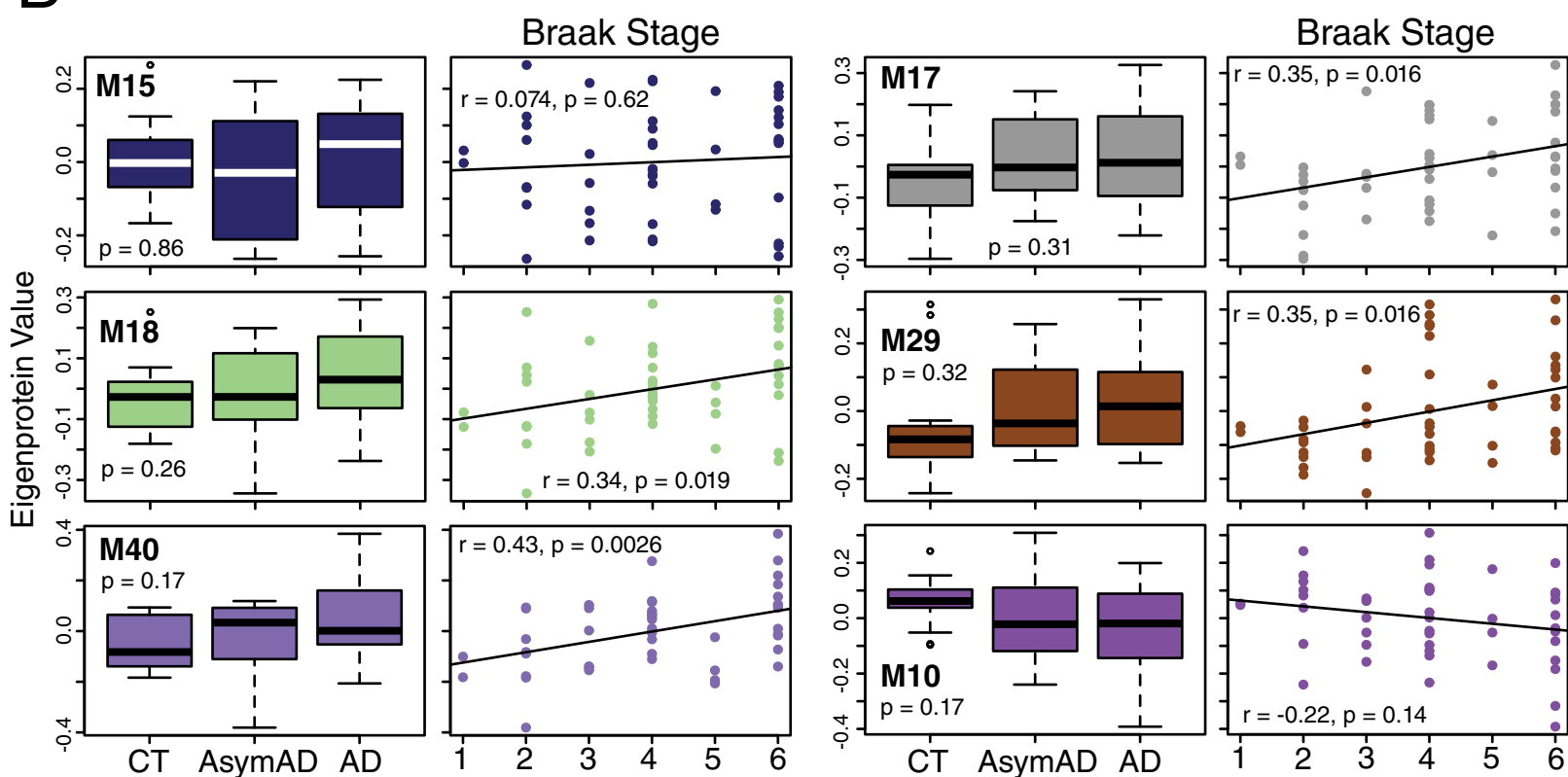

Supplement: Supplementary file 17 — Figure S10. RNA Binding Protein Enrichment in TMT Network Modules and Correlation with AD Pathology. (A, B) Overlap of different groups of RNA binding proteins within TMT network modules (A). Significance of overlap was calculated by Fisher exact test, and is shown by single color heat map of -log10 p value (increased red represents smaller p value and increased overlap). P values are corrected by Benjamini-Hochberg FDR. A, McKnight 570 refers to RNA binding proteins that are often found within RNA granules as described in [48]; B, Total Observed RNA binding refers to all RNA binding proteins commonly observed in our proteomic experiments; C, proteins that interact with the low complexity 2 (LC2) domain of the U1-70K small nuclear ribonucleoprotein 70 kDa (snRNP70) [54]; D, proteins that are homologous to U1-70K; E, proteins that interact with the LC1 or basic-acidic dipeptide (BAD) repeat domain of U1-70K [54]; F, low complexity arginine-serine (RS) repeat-containing proteins; G, proteins annotated as comprising the spliceosome complex in the Kyoto Encyclopedia of Genes and Genomes (KEGG); H, proteins annotated as involved in RNA translation by Gene Ontology (GO); I, proteins annotated in KEGG as belonging to the U1 spliceosome complex. (B) The six modules most enriched in RNA binding proteins (M15, M18, M40, M17, M29, and M10) were assessed for change by case group and correlation with tau tangle burden (Braak stage). Four out of the six modules significantly correlated with Braak stage. Correlation was performed by the bicorrelation function as implemented in R. CT, control; AsymAD, asymptomatic Alzheimer’s disease; AD, Alzheimer’s disease. (PDF 254 kb) [file 13024_2018_282_MOESM17_ESM.pdf]

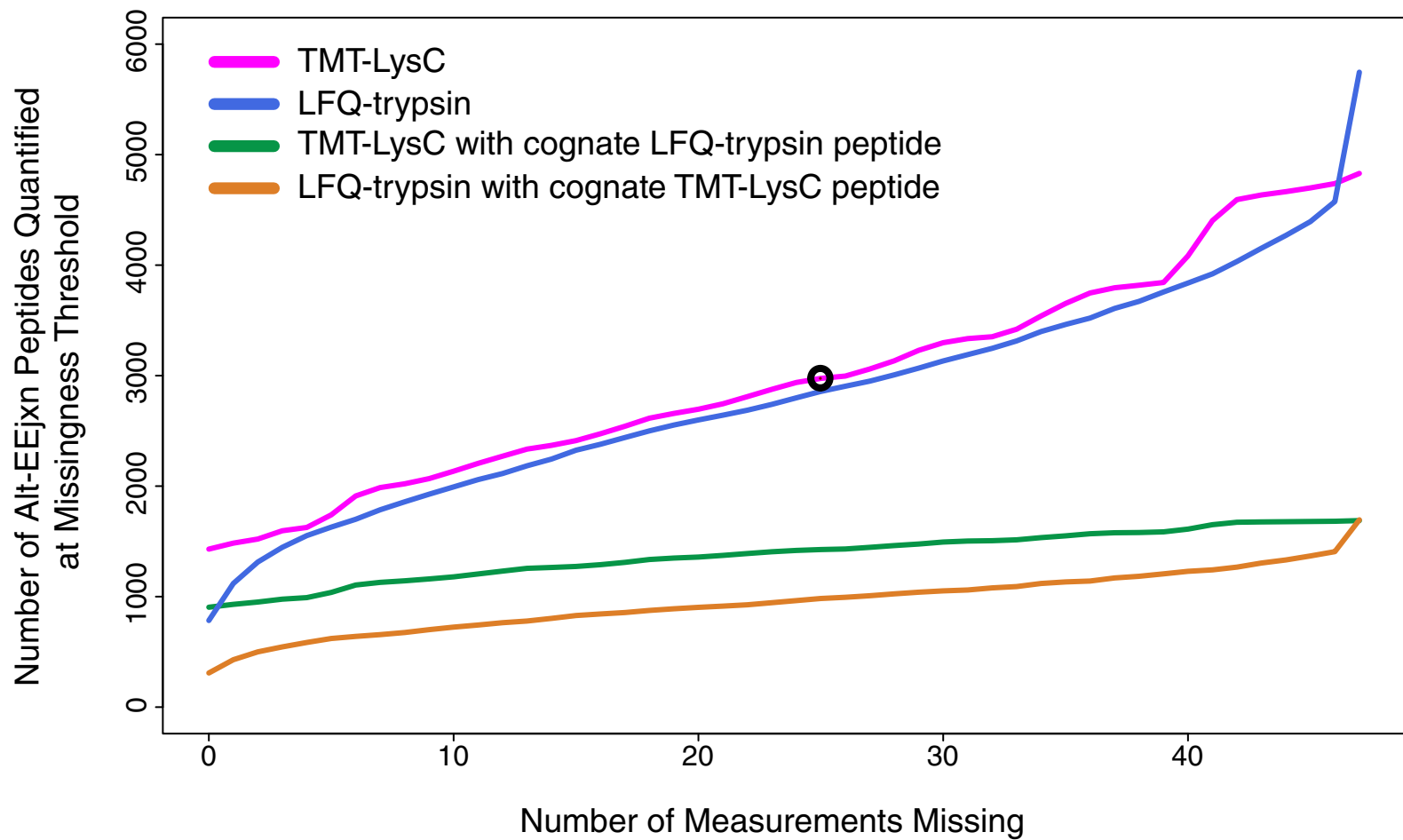

Supplement: Supplementary file 18 — Figure S11. Alternative Exon-Exon Junction Peptide Quantitation in TMT-LysC and LFQ-trypsin Analyses. The relationship between the number of quantifiable alternative exon-exon junction (alt-EEjxn) peptides at a given threshold of missing values in the 47 brain samples from the BLSA cohort for TMT-LysC and LFQ-trypsin analyses is shown, without regard to case group. Also shown is the number of alt-EEjxn peptides quantified by TMT-LysC that had a LFQ-trypsin cognate peptide, as well as the number of alt-EEjxn peptides quantified by LFQ-trypsin that had a cognate TMT-LysC peptide. The point at 23 samples represents the 50% missingness threshold. (PDF 89 kb) [file 13024_2018_282_MOESM18_ESM.pdf]

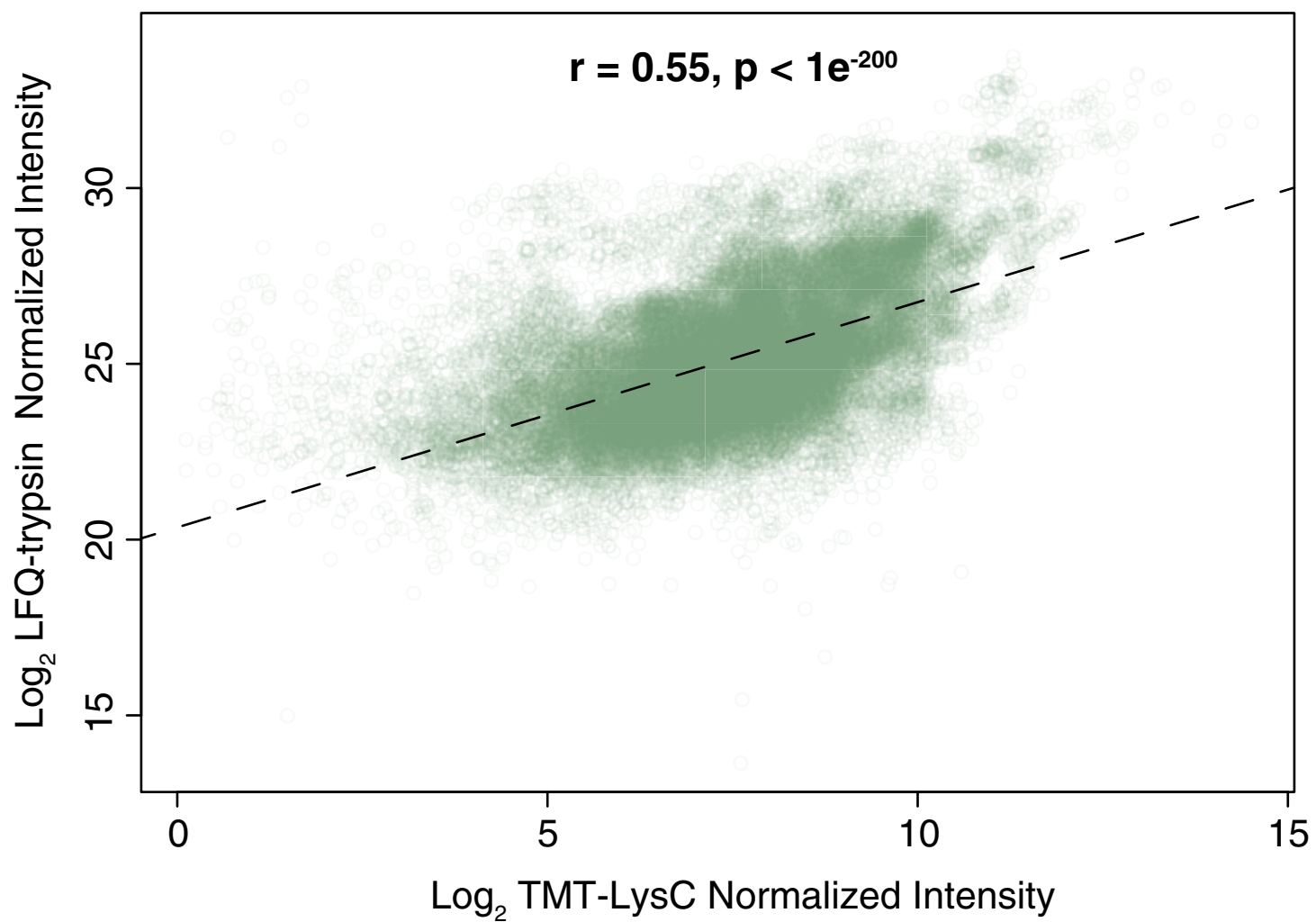

Supplement: Supplementary file 19 — Figure S12. Correlation Between Alternative Exon-Exon Junctions Quantified by TMT-LysC and LFQ-trypsin Analyses. Alternative exon-exon junctions (alt-EEjxns) that were identified and quantified in both TMT-LysC and LFQ-trypsin analyses (n = 1202 alt-EEjxns) and which had no missing values across the 47 BLSA cases were matched case-to-case, and the log(2) normalized intensity measurements for each alt-EEjxn were correlated between the two quantification approaches. Note that the peptide containing the alt-EEjxn is not necessarily identical between TMT-LysC and LFQ-trypsin analyses. When the correlation is restricted to identical alt-EEjxn peptides (n = 728), the strength of correlation increases only slightly (r = 0.6) (data not shown). (PDF 15000 kb) [file 13024_2018_282_MOESM19_ESM.pdf]

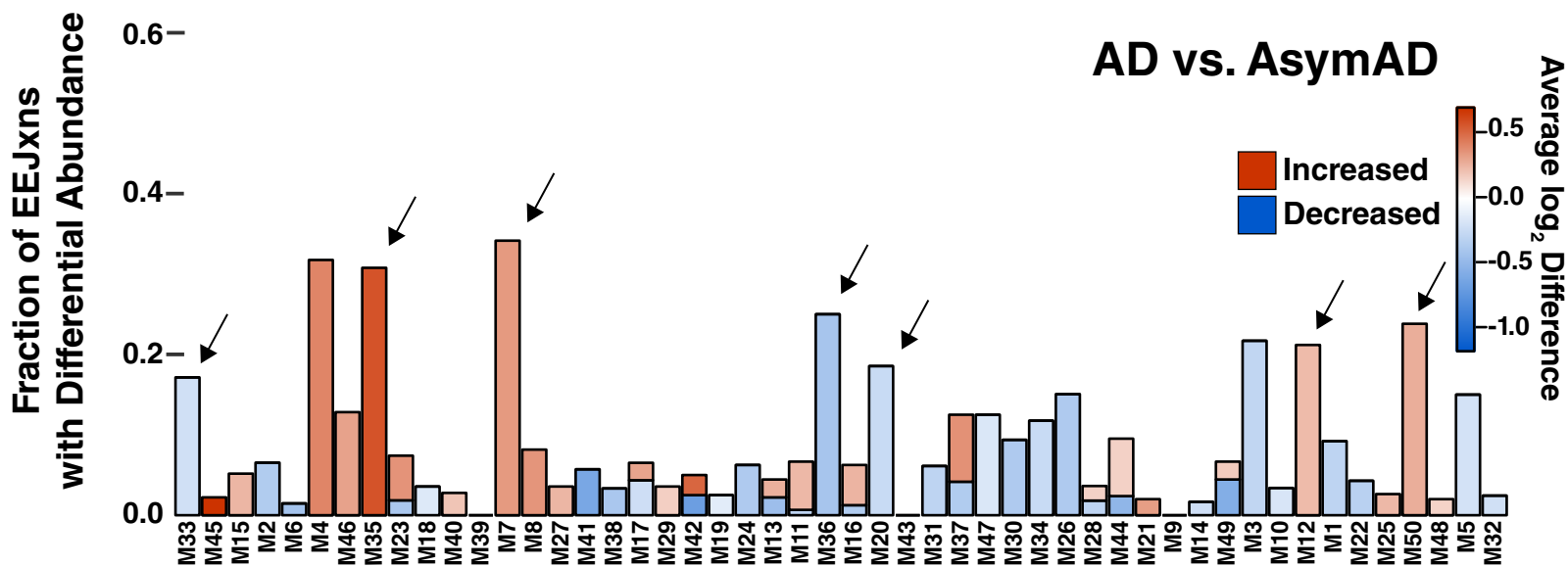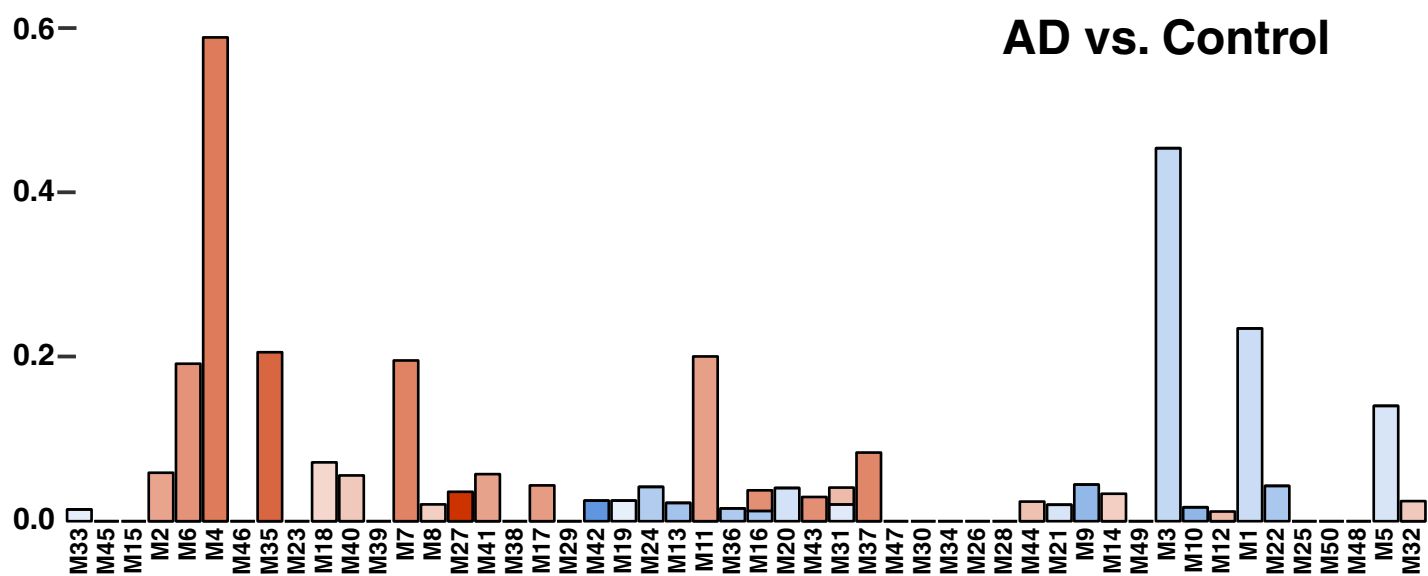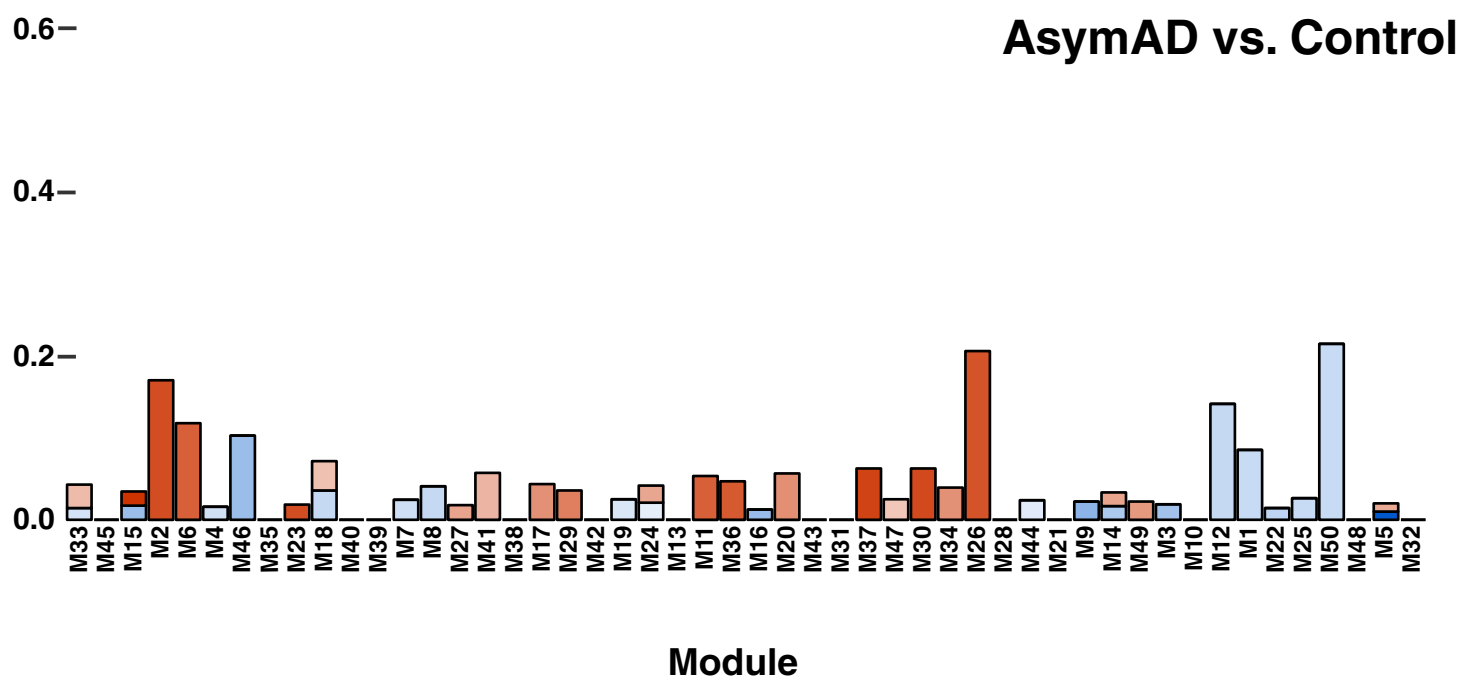

Supplement: Supplementary file 21 — Figure S14. Differential Abundance of Alternative Exon-Exon Junction Peptides by TMT Network Module. For case group comparisons AD vs. AsymAD (top), AD vs. control (middle), and AsymAD vs. control (bottom), the fraction of alternative exon-exon junction (alt-EEjxn) peptides within each network module that were significantly different between the two case groups was plotted by bar graph, with each bar color coded according to the average log2 difference of the alt-EEjxn peptides in each direction (increased or decreased). The arrows in the AD vs. AsymAD comparison highlight modules that showed an increase in the fraction of alt-EEjxns or an increase in the magnitude of differential abundance, or both, compared to AD vs. control. (PDF 718 kb) [file 13024_2018_282_MOESM21_ESM.pdf]

# M18 Lightgreen Module

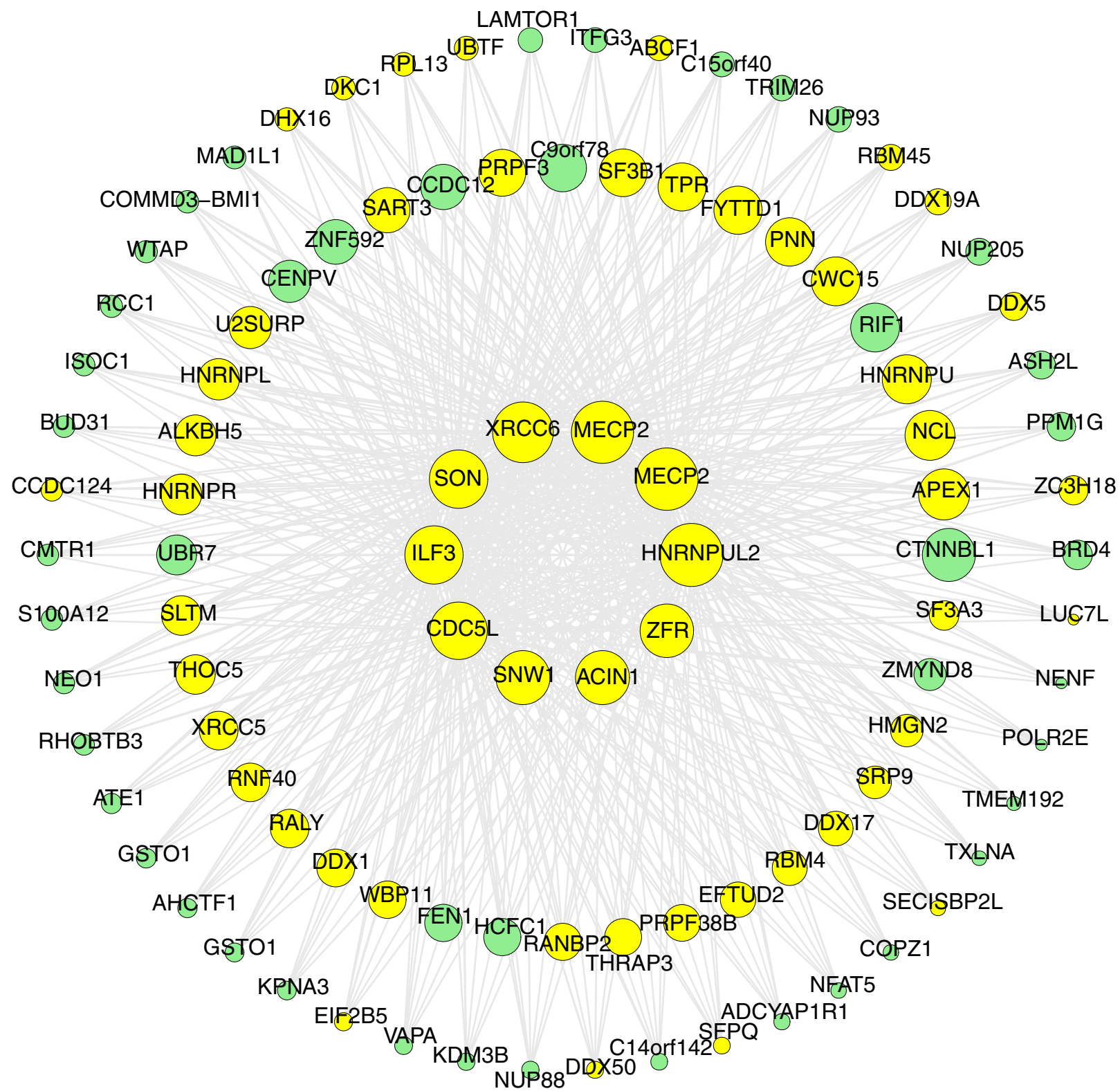

Supplement: Supplementary file 22 — Figure S15. Enrichment of RNA Binding Proteins in TMT Network Module 18. Graphical representation of the correlation relationships among proteins for lightgreen module M18, with proteins centrally located representing those most highly correlated with other proteins in the module. Proteins annotated as RNA binding proteins in geneontology.org are highlighted in yellow. (PDF 177 kb) [file 13024_2018_282_MOESM22_ESM.pdf]
