# Supplementary material for: Deep proteomic network analysis of Alzheimer’s disease brain reveals alterations in RNA binding proteins and RNA splicing associated with disease
Source: Mol Neurodegener. 2018 Oct 4;13:52. doi: 10.1186/s13024-018-0282-4 (PMC6172707; doi:10.1186/s13024-018-0282-4)
Supplement: Supplementary file 11 — Figure S4. TMT Network Modules Associated with Disease State or AD Pathology. (A-F) TMT network modules that were enriched in astrocyte or microglial proteins (A), neuronal proteins (B), ‘de nove’ post-translational protein folding machinery (C), mitochondrial proteins (D), nucleosomal proteins (E), or RNA-associated proteins (F), and which also changed with disease state or were correlated to AD pathology are shown, along with the top six hub proteins for each module. The full list of modules and pathological correlations for each module is provided in Supplementary Data. Eigenprotein differences by disease state were assessed by one-way ANOVA. (PDF 180 kb) [file 13024_2018_282_MOESM11_ESM.pdf]

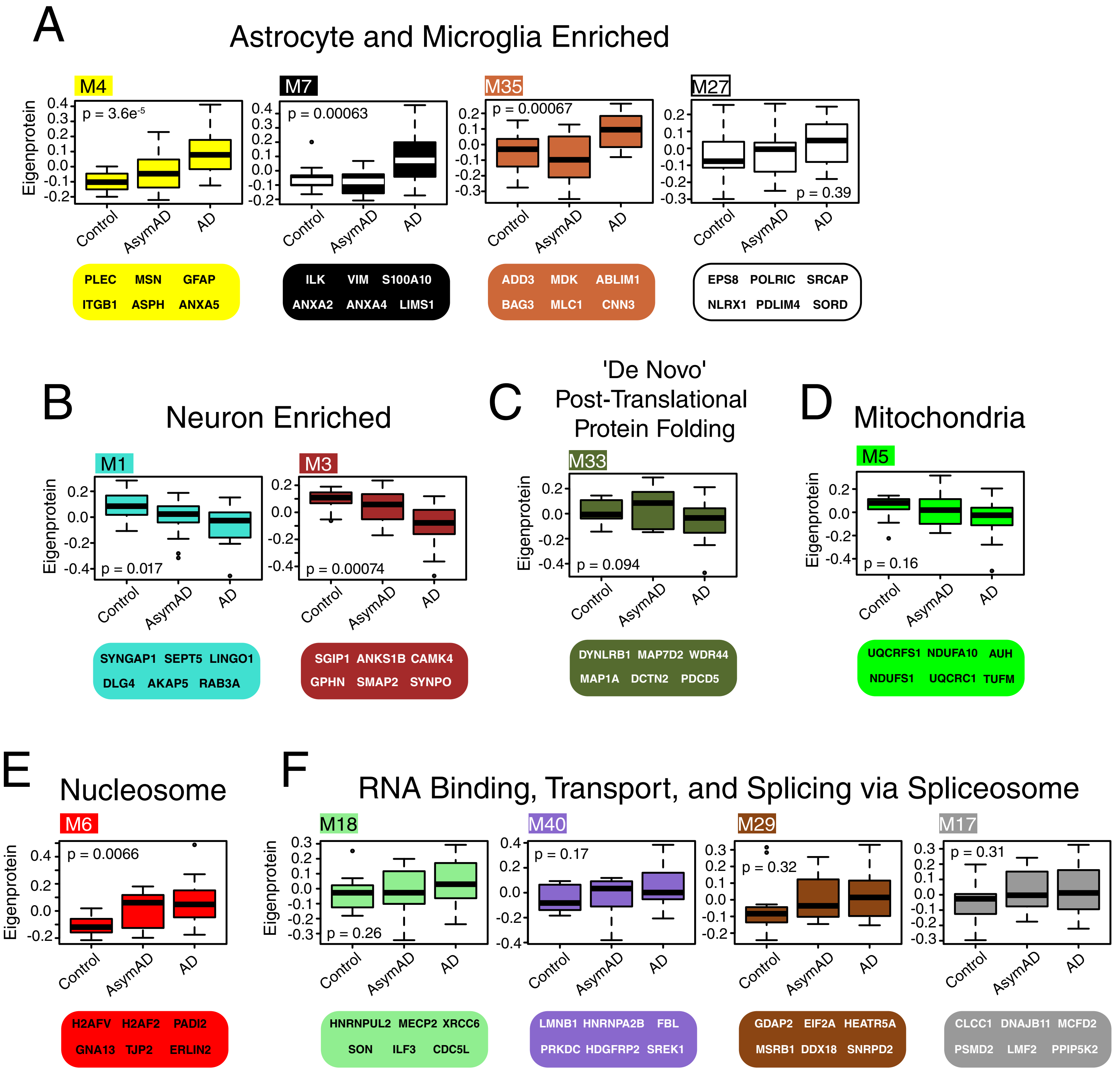
A
Astrocyte and Microglia Enriched
M4
p = 3.6e-5
M7
p = 0.00063
M35
p = 0.00067
M27
p = 0.39
Eigenprotein
Control
AsymAD
AD
PLEC MSN GFAP
ITGB1 ASPH ANXA5
ILK VIM S100A10
ANXA2 ANXA4 LIMS1
ADD3 MDK ABLIM1
BAG3 MLC1 CNN3
EPS8 POLRIC SRCAP
NLRX1 PDLIM4 SORD
B
Neuron Enriched
M1
p = 0.017
M3
p = 0.00074
SYNGAP1 SEPT5 LINGO1
DLG4 AKAP5 RAB3A
SGIP1 ANKS1B CAMK4
GPHN SMAP2 SYNPO
C
'De Novo'
Post-Translational
Protein Folding
M33
p = 0.094
DYNLRB1 MAP7D2 WDR44
MAP1A DCTN2 PDCD5
D
Mitochondria
M5
p = 0.16
UQCRFS1 NDUFA10 AUH
NDUFS1 UQCRC1 TUFM
E
Nucleosome
M6
p = 0.0066
H2AFV H2AF2 PADI2
GNA13 TJP2 ERLIN2
F
RNA Binding, Transport, and Splicing via Spliceosome
M18
p = 0.26
M40
p = 0.17
M29
p = 0.32
M17
p = 0.31
HNRNPUL2 MECP2 XRCC6
SON ILF3 CDC5L
LMNB1 HNRNPA2B FBL
PRKDC HDGFRP2 SREK1
GDAP2 EIF2A HEATR5A
MSRB1 DDX18 SNRPD2
CLCC1 DNAJB11 MCFD2
PSMD2 LMF2 PPIP5K2
