# Supplementary material for: Deep proteomic network analysis of Alzheimer’s disease brain reveals alterations in RNA binding proteins and RNA splicing associated with disease
Source: Mol Neurodegener. 2018 Oct 4;13:52. doi: 10.1186/s13024-018-0282-4 (PMC6172707; doi:10.1186/s13024-018-0282-4)
Supplement: Supplementary file 20 — Figure S13. GO Analysis of Alternative Exon-Exon Junction Peptides Unique to the RNAseq Database. Alternative exon-exon junction (alt-EEjxn) peptides that were identified by LFQ-trypsin or TMT-LysC approaches from the RNAseq data only were analyzed by gene ontology (GO), which showed that the alternatively spliced proteins identified by the two approaches in the RNAseq data were largely unique. (PDF 660 kb) [file 13024_2018_282_MOESM20_ESM.pdf]

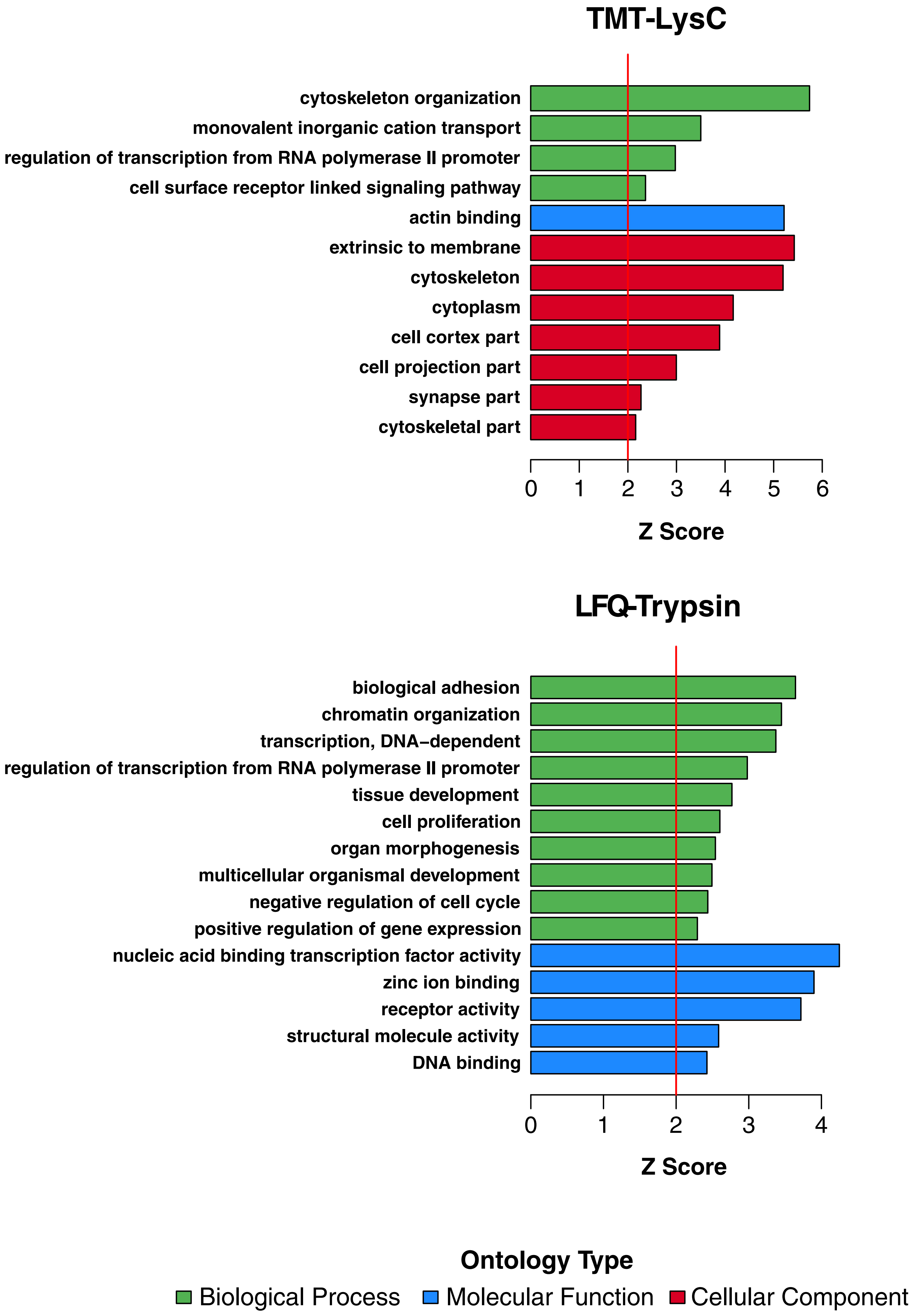

TMT-LysC
cytoskeleton organization
monovalent inorganic cation transport
regulation of transcription from RNA polymerase II promoter
cell surface receptor linked signaling pathway
actin binding
extrinsic to membrane
cytoskeleton
cytoplasm
cell cortex part
cell projection part
synapse part
cytoskeletal part
0
1
2
3
4
5
6
Z Score
LFQ-Trypsin
biological adhesion
chromatin organization
transcription, DNA–dependent
regulation of transcription from RNA polymerase II promoter
tissue development
cell proliferation
organ morphogenesis
multicellular organismal development
negative regulation of cell cycle
positive regulation of gene expression
nucleic acid binding transcription factor activity
zinc ion binding
receptor activity
structural molecule activity
DNA binding
0
1
2
3
4
Z Score
Ontology Type
Biological Process
Molecular Function
Cellular Component
